# Genome-wide determinants of mortality and motor progression in Parkinson’s disease

**DOI:** 10.1038/s41531-024-00729-8

**Published:** 2024-06-07

**Authors:** Manuela M. X. Tan, Michael A. Lawton, Miriam I. Pollard, Emmeline Brown, Raquel Real, Alejandro Martinez Carrasco, Samir Bekadar, Edwin Jabbari, Regina H. Reynolds, Hirotaka Iwaki, Cornelis Blauwendraat, Sofia Kanavou, Leon Hubbard, Naveed Malek, Katherine A. Grosset, Nin Bajaj, Roger A. Barker, David J. Burn, Catherine Bresner, Thomas Foltynie, Nicholas W. Wood, Caroline H. Williams-Gray, Ole A. Andreassen, Mathias Toft, Alexis Elbaz, Fanny Artaud, Alexis Brice, Jean-Christophe Corvol, Jan Aasly, Matthew J. Farrer, Michael A. Nalls, Andrew B. Singleton, Nigel M. Williams, Yoav Ben-Shlomo, John Hardy, Michele T. M. Hu, Donald G. Grosset, Maryam Shoai, Lasse Pihlstrøm, Huw R. Morris

**Affiliations:** 1 Department of Neurology, Oslo University Hospital, Oslo, Norway; 2 Department of Clinical and Movement Neurosciences, Queen Square Institute of Neurology, University College London, London, UK; 3 UCL Movement Disorders Centre, University College London, London, UK; 4 Population Health Sciences, Bristol Medical School, University of Bristol, Bristol, UK; 5 Aligning Science Across Parkinson’s (ASAP) Collaborative Research Network, Chevy Chase, MD 20815 USA; 6 Sorbonne University, Paris Brain Institute - ICM, Inserm, CNRS, Assistance Publique Hôpitaux de Paris, Departement of Neurology, Hôpital Pitié-Salpêtrière, Paris, France; 7 Genetics and Genomic Medicine, Great Ormond Street Institute of Child Health, University College London, London, UK; 8 Laboratory of Neurogenetics, National Institute on Aging, National Institutes of Health, Bethesda, MD USA; 9 Data Tecnica, Washington DC, USA; 10 Center for Alzheimer’s and Related Dementias, National Institutes of Health, Bethesda, MD USA; 11 Institute of Psychological Medicine and Clinical Neurosciences, MRC Centre for Neuropsychiatric Genetics and Genomics, Cardiff University, Cardiff, UK; 12 Department of Neurology, Institute of Neurological Sciences, Queen Elizabeth University Hospital, Glasgow, UK; 13 Clinical Neurosciences, University of Nottingham, Nottingham, UK; 14 John Van Geest Centre for Brain Repair, Department of Clinical Neurosciences, University of Cambridge, Cambridge, UK; 15 Cambridge Stem Cell Institute, University of Cambridge, Cambridge, UK; 16 Faculty of Medical Sciences, Newcastle University, Newcastle upon Tyne, UK; 17 NORMENT, Division of Mental Health and Addiction, Oslo University Hospital, Oslo, Norway; 18 Institute of Clinical Medicine, Faculty of Medicine, University of Oslo, Oslo, Norway; 19 Paris-Saclay University, UVSQ, Inserm, Gustave Roussy, “Exposome and Heredity” team, CESP, 94807 Villejuif, France; 20 Department of Neurology, St. Olavs Hospital, Trondheim, Norway; 21 Department of Neuromedicine and Movement Science (INB), Faculty of Medicine and Health Sciences, Norwegian University of Science and Technology (NTNU), Trondheim, Norway; 22 Department of Neurology, University of Florida, Gainesville, FL USA; 23 Department of Neurodegenerative Diseases, Queen Square Institute of Neurology, University College London, London, UK; 24 Reta Lila Weston Institute, UCL Queen Square Institute of Neurology, London, UK; 25 UK Dementia Research Institute, University College London, London, UK; 26 National Institute for Health Research (NIHR) University College London Hospitals Biomedical Research Centre, London, UK; 27 Institute for Advanced Study, The Hong Kong University of Science and Technology, Hong Kong SAR, China; 28 Nuffield Department of Clinical Neurosciences, Division of Clinical Neurology, University of Oxford, Oxford, UK; 29 Oxford Parkinson’s Disease Centre, University of Oxford, Oxford, UK; 30 Department of Clinical Neurology, Oxford University Hospitals NHS Foundation Trust, Oxford, UK; 31 School of Neuroscience and Psychology, University of Glasgow, Glasgow, UK

**Keywords:** Genome-wide association studies, Parkinson's disease

## Abstract

There are 90 independent genome-wide significant genetic risk variants for Parkinson’s disease (PD) but currently only five nominated loci for PD progression. The biology of PD progression is likely to be of central importance in defining mechanisms that can be used to develop new treatments. We studied 6766 PD patients, over 15,340 visits with a mean follow-up of between 4.2 and 15.7 years and carried out genome-wide survival studies for time to a motor progression endpoint, defined by reaching Hoehn and Yahr stage 3 or greater, and death (mortality). There was a robust effect of the **APOE** ε4 allele on mortality in PD. We also identified a locus within the **TBXAS1** gene encoding thromboxane A synthase 1 associated with mortality in PD. We also report 4 independent loci associated with motor progression in or near **MORN1**, **ASNS**, **PDE5A**, and **XPO1**. Only the non-Gaucher disease causing **GBA1** PD risk variant E326K, of the known PD risk variants, was associated with mortality in PD. Further work is needed to understand the links between these genomic variants and the underlying disease biology. However, these may represent new candidates for disease modification in PD.

## Introduction

Parkinson’s disease (PD) is a progressive neurodegenerative condition for which there are no drug treatments to stop or slow disease progression. Large-scale genome-wide case-control association studies (GWASs) of PD have identified 90 independent variants associated with disease risk^1^. However, it is also important to study the genetics and biology of disease progression. This will enable the development of potential disease-modifying treatments. There have now been a handful of GWAS that aim to identify genetic variants associated with progression in PD. These have nominated loci in *SLC44A1* (encoding choline transporter-like protein -1, involved in membrane synthesis) for progression to Hoehn and Yahr (H&Y) stage 3 or greater^2^, *APOE* for cognitive progression^3^, *LRP1B* (encoding a low-density lipoprotein receptor which is involved in amyloid precursor protein trafficking) for progression to dementia^4^, and *RIMS2* (encoding the RAB3 interacting RIMS2 protein, involved in neurotransmitter release) for progression to PD dementia^5^. In addition, many candidate gene studies have reported that variants in *GBA1*, *APOE*, and *MAPT*, are associated with the rate of PD motor and cognitive progression^6^.

PD progression may be determined by differential cellular susceptibility, related to mitochondrial function or proteostasis, differential cell-to-cell spread of pathology, or novel pathways and mechanisms. Risk factors determined from case-control studies indicate aetiological pathways and guide future preventive trials, but these may differ from risk factors that determine disease progression. Currently disease-modifying treatment trials focus on intervention in recently diagnosed patients, related to disease progression after diagnosis. Work on large-scale longitudinal cohorts over the last ten years has now enabled the collaborative study of large clinico-genetic datasets. Here we have carried out two progression GWASs: progression to mortality, and H&Y stage 3 or greater (H&Y3+). We have analysed data from 6766 PD patients with over 15,340 visits and mean follow-up ranging between 4.2 to 15.7 years.

## Results

Overall 6766 participants with PD were analysed with mean follow-up between 4.2 and 15.7 years (Table 1). We did not have data from regular follow-up visits for all studies as some studies only contributed mortality data. However, in the studies that had regular follow-up visit data available, over 15,340 visits were analysed (Table 1).

**Table 1:** Cohort demographics

Cohort	N PD patients	N male (%)	Age onset, years	Age diagnosis, years	Age entry, years	Disease duration at baseline (time from symptom onset to baseline), years	N clinical observations	Follow-up, years	N who died during follow-up, as a fraction of the total number of individuals included for mortality analysis (%)	N who met Hoehn and Yahr 3+ during follow-up, as a fraction of the total number of individuals included for Hoehn and Yahr analysis (%)*	Array
Tracking Parkinson’s	1782	1154 (64.8%)	64.4 (9.8)	66.2 (9.3)	67.5 (9.3)	3.2 (3.0)	7629	4.2 (1.7)	133/1779 (7.5%)	395/1641 (24.1%)	Illumina HumanCore Exome array with custom content (over 27,000 custom variants implicated in neurological and psychiatric disorders)
Oxford Discovery	842	544 (64.6%)	64.5 (9.6)	66.2 (9.5)	67.4 (9.4)	2.9 (1.8)	3181	4.7 (2.8)	128/762 (16.8%)	119/731 (16.3%)	Illumina HumanCore Exome-12 v1.1 or Illumina InfiniumCoreExome-24 v1.1
PPMI	403	263 (65.3%)	59.5 (10.0)	61.0 (9.7)	61.5 (9.7)	2.0 (1.9)	2200	6.0 (1.8)	18/403 (4.5%)	69/351 (19.7%)	Whole genome sequencing (see https://www.ppmi-info.org/). Only variants passing quality filters (PASS)
QSBB	308	187 (60.7%)	61.8 (10.1)	NA	NA	NA	NA	15.7 (7.7)	285/285 (100%)	NA	Illumina NeuroChip (Illumina Infinium HumanCore-24 array with approximately 180,000 custom variants implicated in neurological diseases
UKB - incident	1174	728 (62.0%)	NA	69.6 (5.5)	64.1 (5.2)	NA	NA	10.1 (2.1)	370/970 (38.1%)	NA	Affymetrix Applied Biosystems BiLEVE Axium Array and UK Biobank Axium Array
UKB - prevalent	839	526 (62.7%)	NA	57.4 (7.1)	62.8 (5.5)	5.5 (4.8)	NA	9.8 (2.5)	294/820 (35.9%)	NA	Affymetrix Applied Biosystems BiLEVE Axium Array and UK Biobank Axium Array
Calypso	186	125 (67.2%)	60.0 (10.0)	61.6 (9.8)	67.7 (9.5)	7.7 (5.3)	NA	9.3 (3.9)	121/180 (67.2%)	NA	Illumina BeadArray Human660-Quad
CamPaIGN	90	49 (54.4%)	67.9 (10.4)	70.0 (9.7)	70.3 (9.7)	2.5 (2.9)	NA	8.2 (4.3)	74/89 (83.1%)	NA	Illumina BeadArray Human660-Quad
Cambridge PD Research clinic	239	152 (63.6%)	58.6 (12.0)	60.4 (11.4)	65.1 (10.5)	6.8 (6.1)	NA	9.0 (5.0)	177/239 (74.1%)	NA	Illumina BeadArray Human660-Quad
DIGPD	376	227 (60.4%)	58.7 (10.0)	59.6 (9.9)	62.2 (9.9)	3.5 (1.6)	1940	9.0 (2.1)	67/337 (19.9%)	76/369 (20.6%)	Illumina Multi-Ethnic Genotyping Array (MEGA) array
Trondheim	192	109 (56.8%)	62.9 (9.7)	NA	NA	NA	390	15.7 (7.1)	189/189 (100%)	NA	Illumina NeuroChip (Illumina Infinium HumanCore-24 array with approximately 180,000 custom variants implicated in neurological diseases)
Oslo	335	226 (67.5%)	52.2 (10.2)	54.2 (10.2)	64.0 (9.2)	11.8 (6.3)	791	9.2 (3.6)	185/333 (55.6%)	94/239 (39.3%)	Illumina Infinium OmniExpress array

Means (SD) are shown unless otherwise indicated. Data shown are only in individuals who had both clinical and genetic data after quality control filters have been applied within each cohort. The number of individuals in each cohort with complete data for each outcome of interest (and covariates) may be less than the total number. Follow-up time is calculated as the time from study entry to last visit, death, or last known status date (censoring), whichever is the latest. For QSBB and Trondheim, we did not have data on age at study entry so follow-up is calculated as the time from onset to death.

*Excluding left-censored individuals, i.e. individuals who met Hoehn and Yahr stage 3 or greater at baseline.

*CamPaIGN* Cambridgeshire Parkinson’s Incidence from GP to Neurologist, *DIGPD* Drug interaction with genes in Parkinson’s Disease, *HY3* Hoehn and Yahr stage 3 or greater, *PPMI* Parkinson’s Progression Markers Initiative, *QSBB* Queen Square Brain Bank pathologically-confirmed PD cases, *UKB* UK Biobank.

### GWAS of mortality

One study was excluded from the meta-analysis of mortality because the study-specific genomic inflation factor was above 1.2 and one study was excluded because less than 20 individuals reached the mortality endpoint (Supplementary Table 1).

5744 patients were included in the meta-analysis of mortality. Of these, 1846 (32.1%) individuals had died with a median time to death of 10.6 years from PD onset. The Kaplan-Meier curve for mortality stratified by cohort is shown in Supplementary Fig. 1. 7,696,389 Single Nucleotide Polymorphisms (SNPs) were present in at least 1,000 individuals and 7,313,918 SNPs passed meta-analysis filtering for heterogeneity and MAF variability. The genomic inflation value of the meta-analysis after filtering was 1.04.

**Fig. 1:**
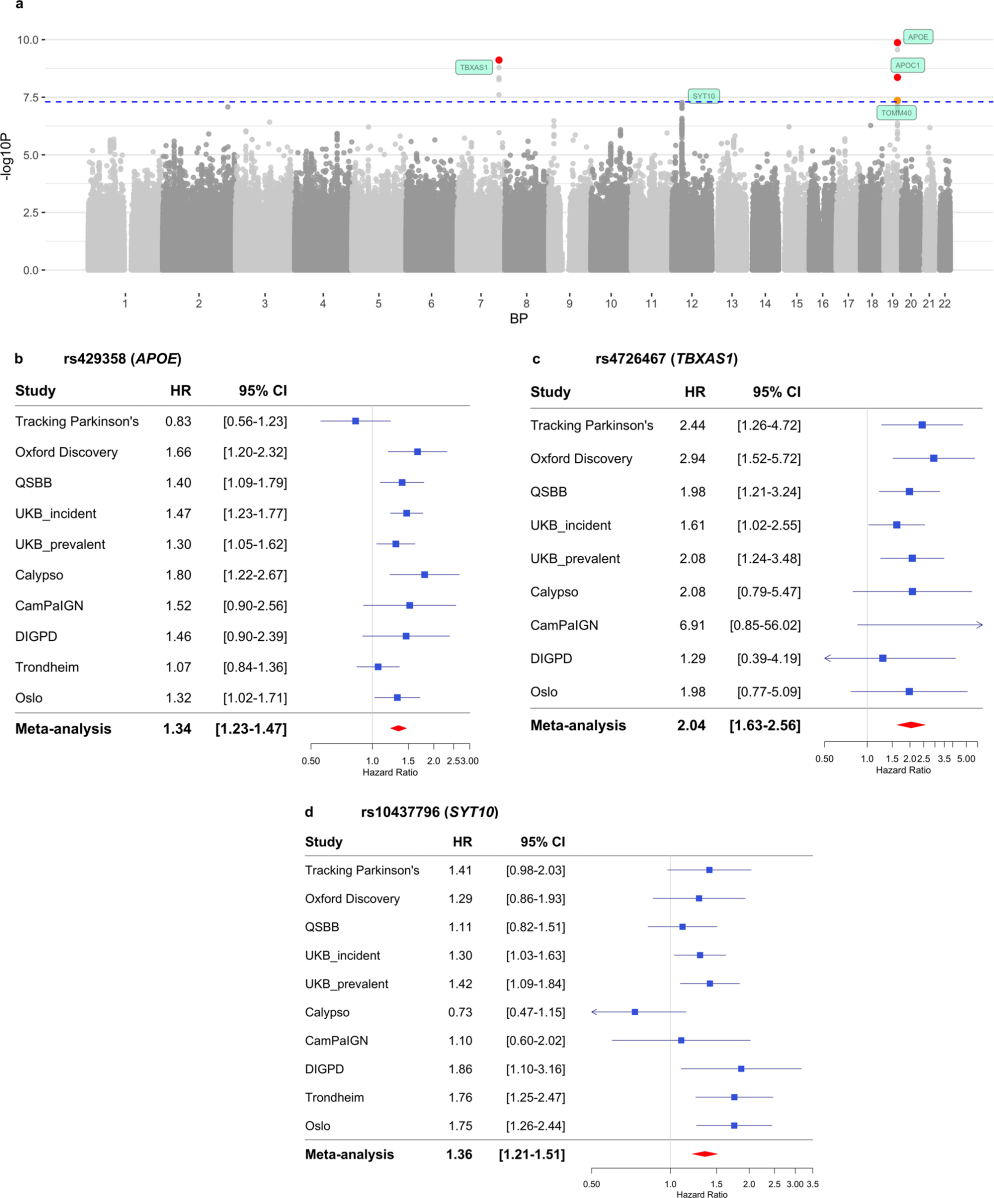
GWAS meta-analysis of mortality. **a** The Manhattan plot showing two GWAS significant loci after meta-analysis. The blue dashed line indicates the threshold for genome-wide significance, *p* = 5 × 10^−8^. SNPs highlighted in red have p-value < 5 × 10^−9^. SNPs highlighted in orange have *p*-value < 5 x 10^−8^. One nominal association in Chromosome 12 is also annotated with the nearest gene, *SYT10* (*p* = 5.3 × 10^−8^). **b** Forest plot for the top SNP rs429358 in Chromosome 19, in *APOE*. **c** Forest plot for the top SNP rs4726467 in Chromosome 7, in *TBXAS1*. **d** Forest plot for the top SNP rs10437796 in Chromosome 12, near *SYT10*. BP Base Pair, CI Confidence Interval, GWAS Genome Wide Association Study. HR Hazard Ratio. SNP Single Nucleotide Polymorphism.

Two loci passed genome-wide significance and were identified to determine mortality in PD (Fig. 1). The top SNP was rs429358 in Chromosome 19 (*p* = 1.4 x 10^−10^), which tags the *APOE* ε4 allele (Table 2). This locus included 12 SNPs in total in linkage disequilibrium (r^2^ ≥ 0.6) with the independent significant SNP. One other locus in Chromosome 7 in *TBXAS1* also reached significance (*p* = 7.7 x 10^−10^), and another locus in Chromosome 12 near *SYT10* was nominally associated (p = 5.3 x 10^−8^). Regional association plots are shown in Fig. 1 and Supplementary Figs. 2–4. The top ten independent SNPs identified from GCTA-COJO and their nearest genes are reported in Table 2.

**Table 2:** Top 10 independent SNPs from meta-analysis of progression to mortality

chr	bp	rsID	effect allele	non-effect allele	effect allele freq	nearest gene	distance to gene (BP)	beta	SE	Hazard Ratio	95% CI	*p*-value	*p*-value COJO
19	45411941	rs429358	C	T	0.160	*APOE*	0	0.295	0.046	1.342	1.23–1.47	1.35E−10	1.60E−10
7	139637422	rs4726467	T	C	0.021	*TBXAS1*	0	0.713	0.116	2.039	1.63–2.56	7.71E−10	8.49E−10
12	33635494	rs10437796	A	C	0.098	*SYT10*	42740	0.304	0.056	1.355	1.21–1.51	5.31E−08	5.76E−08
9	17616880	rs3808753	G	A	0.035	*SH3GL2*	0	0.448	0.088	1.564	1.32–1.86	3.34E−07	3.52E−07
3	113979619	rs142285045	A	C	0.013	*ZNF80*	23194	0.836	0.165	2.308	1.67–3.19	3.80E−07	4.06E−07
18	33965217	rs76125680	C	G	0.012	*FHOD3*	0	0.874	0.174	2.397	1.70–3.37	5.32E−07	5.65E−07
15	34896795	rs35294489	G	A	0.014	*GOLGA8B*	20959	0.757	0.152	2.131	1.58–2.87	6.07E−07	6.43E−07
5	55533638	rs28811891	T	C	0.026	*ANKRD55*	4452	0.544	0.109	1.722	1.39–2.13	6.21E−07	6.49E−07
21	31807104	rs145506557	C	A	0.016	*KRTAP13-4*	4028	0.896	0.180	2.451	1.72–3.49	6.59E−07	7.27E−07
10	98786658	rs17112311	A	G	0.049	*C10orf12*	41073	0.364	0.074	1.439	1.25–1.66	8.07E−07	8.31E−07

Independent SNPs identified with GCTA-COJO. Genome coordinates are in build hg19/GRCh37.

*BP* base pair, *chr* chromosome, *CI* Confidence Interval, freq frequency, *GCTA-COJO* Genome-wide Complex Trait Analysis conditional and joint analysis, *SE* Standard Error, *SNP* Single Nucleotide Polymorphism.

We performed MAGMA gene- and gene-set analysis within FUMA (Functional Mapping and Annotation of Genome-Wide Association Studies; https://fuma.ctglab.nl/)^7^ to aggregate variant data to the level of whole genes or groups of genes^8^. In the MAGMA gene-based test, *APOE* was significantly associated with mortality (*p* = 1.9 x 10^−10^), and *SYT10* was associated just below genome-wide significance (*p* = 3.6 x 10^−6^). No gene sets or tissues were significantly associated with mortality in the MAGMA analysis.

A locus in *TBXAS1* was also significantly associated with PD mortality, with the top SNP rs4726467 and 5 additional SNPs in linkage disequilibrium (r^2^ ≥ 0.6). This SNP is an expression quantitative trait locus (eQTL), with the effect (minor) allele decreasing expression of *TBXAS1* in the blood (eQTLGen; https://www.eqtlgen.org/)^9^ but not other tissues as reported in GTEx (https://gtexportal.org/). There was no evidence on GTEx that rs4726467 is a splicing Quantitative Trait Loci (sQTLs). Brain eQTL data at MetaBrain (https://www.metabrain.nl/)^10^ did not indicate this SNP was a significant cis-eQTL in any brain regions from European samples.

We also searched the public database LDproxy (https://ldlink.nci.nih.gov/) to look for coding variants that may be tagged by the top SNPs which could provide insight into the causal variants, genes, and their functions. We identified two coding variants in linkage disequilibrium (LD) within 500 kb of the top SNP in *TBXAS1*. One was a synonymous variant in *HIPK2*, rs34093649 (D’ = 0.24, R^2^ = 0.05, MAF 0.02) but this was not significant in the GWAS (*p* = 0.39). There was also one missense variant in *PARP12*, rs2286196, about 89 kb away from rs4726467 (D’ = 0.34, R^2^ = 0.01, MAF = 0.20), which was present in the GWAS meta-analysis with nominal significance (*p* = 0.03).

The top SNP in Chromosome 12, rs10437796, is not directly within *SYT10* but increases *SYT10* expression in the testis and decreases expression in the tibial nerve. The SNP also increases the expression of the long noncoding RNA (lncRNA) RP11-438D14.2 (ENSG00000259937) in the brain. This is a ‘sense intronic’ transcript to *SYT10*, a long non-coding transcript that is within an intron of a coding gene and does not overlap any exons. Brain cis-eQTL data from MetaBrain showed that the effect allele A of rs10437796 significantly increased the expression of *SYT10* in the cortex but not in other brain regions. This SNP was not an eQTL or sQTL for any genes in the blood in eQTLGen. There were no coding variants in LD with this SNP in LDproxy within a 500 kb window.

We also performed colocalization analysis to determine whether the association signals for PD mortality and gene expression are driven by a shared causal variant (see Methods; Supplementary Table 2). We used cis-eQTL data from PsychENCODE and eQTLGen to examine gene expression in the whole brain or blood, respectively. However, no PD mortality loci showed evidence of colocalization with eQTLs (PP.H4 < 0.75; Supplementary Table 2).

We checked the top SNPs and genes (+/− 1 Mb) from the mortality GWAS in the most recent longevity GWAS^11^ to see if these were influencing PD-specific or more general population-based mortality and survival. *APOE* and specifically the ε4 tagging SNP, rs429358, was the strongest signal for longevity (beta = −0.05, *p* = 9.6 x 10^−127^). The *TBXAS1* SNP, rs4726467, was not associated with longevity (*p* = 0.82). Other SNPs within or just outside the *TBXAS1* gene boundaries were also not associated with longevity at genome-wide significance. The nearest associated SNP was rs149577943 which lay approximately 1 Mb outside of *TBXAS1* (*p* = 6.1 x 10^-5^). The *SYT10* SNP, rs10437796, was nominally associated with reduced longevity (beta = −0.008, *p* = 0.003) in the longevity GWAS.

There was evidence of sexual dimorphism for the *APOE* ε4 effect on PD mortality, similar to what was observed in the longevity study by Timmers et al. ^11^. We analysed the effect of the *APOE* ε4 SNP rs429358 in men and women separately in each cohort, then meta-analysed the results with a random-effects meta-analysis. Differences in the effect size in men vs. women were tested using the formula: (β_men_ – β_women_)/sqrt(SE_men_2 + SE_women_2), where SE is the standard error of the effect estimate. This statistic follows the Z distribution. The effect of *APOE* ε4 on PD mortality was stronger in women than in men (beta_women_ = 0.54, SE_women_ = 0.08 vs beta_men_ = 0.23, SE_women_ = 0.05, p_diff_ = 9.72 x 10^-4^).

### GWAS of H&Y3+

3331 individuals across 5 cohorts were analysed for progression to H&Y3+ (Supplementary Fig. 5). 753 individuals (22.6%) met the outcome of H&Y3+, with a median time of 3.1 years. 6,549,622 SNPs passed filtering for heterogeneity and MAF variability. The genomic inflation factor of the meta-analysis was 1.03. The top ten independent SNPs from GCTA-COJO are reported in Table 3.

**Table 3:** Top 10 independent SNPs from meta-analysis of progression to Hoehn and Yahr stage 3 or greater

chr	bp	rsID	effect allele	non-effect allele	effect allele freq	nearest gene	distance to gene (BP)	beta	SE	Hazard Ratio	95% CI	*p*-value	*p*-value COJO
1	2315032	rs115217673	A	G	0.016	*MORN1*	0	1.016	0.172	2.762	1.97-3.87	3.09E−09	3.53E−09
7	97470925	rs145274312	A	G	0.011	*ASNS*	10504	1.872	0.317	6.503	3.49-12.11	3.49E−09	NA*
4	120566153	rs113120976	T	C	0.013	*PDE5A*	16172	1.581	0.273	4.859	2.85-8.30	6.95E−09	8.72E−09
2	61742356	rs141421624	G	A	0.017	*XPO1*	0	0.923	0.167	2.517	1.82-3.49	3.08E−08	3.36E−08
1	231267735	rs74796254	T	C	0.027	*LOC149373*	52110	0.693	0.131	2.000	1.55-2.58	1.17E−07	1.27E−07
1	247425485	rs72771919	G	A	0.021	*VN1R5*	5038	0.770	0.146	2.160	1.62-2.88	1.44E−07	1.55E−07
4	186697549	rs75614365	C	G	0.121	*SORBS2*	0	0.372	0.071	1.450	1.26-1.67	1.94E−07	2.13E−07
1	43105085	rs75140767	G	A	0.028	*PPIH*	18621	0.715	0.140	2.044	1.55-2.69	3.26E−07	3.49E−07
2	131557187	rs7566590	A	G	0.042	*AMER3*	31480	0.573	0.112	1.774	1.42-2.21	3.31E−07	3.58E−07
6	11284774	rs148949229	A	G	0.014	*NEDD9*	0	1.694	0.334	5.441	2.83-10.46	3.78E−07	4.72E−07
15	82198898	rs58698247	C	T	0.024	*MEX3B*	135221	0.692	0.136	1.997	1.53-2.61	3.94E−07	4.20E−07

Independent SNPs identified with GCTA-COJO. Genome coordinates are in build hg19/GRCh37.

*One SNP, rs145274312, was not included in the COJO analysis as it was not present in the AMP-PD reference dataset. This is likely because the minor allele frequency is close to 1% in the general population (0.97% in gnomAD non-Finnish European population), however in our PD datasets the allele frequency was >1%.

*BP* base pair, chr chromosome, *CI* Confidence Interval, freq frequency, *GCTA-COJO* Genome-wide Complex Trait Analysis conditional and joint analysis, *SE* Standard Error, *SNP* Single Nucleotide Polymorphism.

**Fig. 2:**
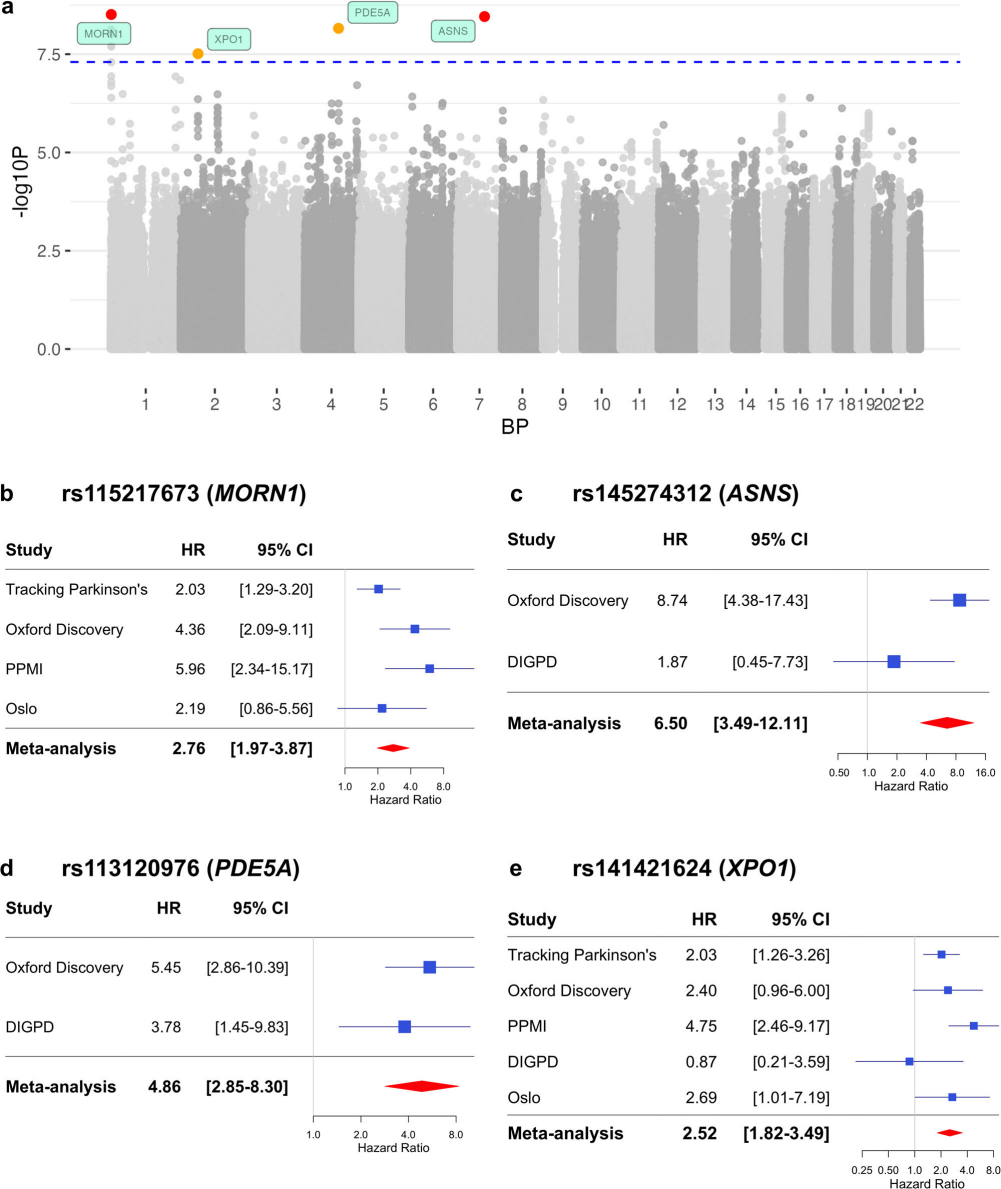
GWAS meta-analysis of progression to Hoehn and Yahr stage 3 or greater (H&Y3+). Note that the loci in the Manhattan plot and the forest plots have been labelled with the physically closest genes, though these may not necessarily be the causal genes. **a** The Manhattan plot showing four GWAS significant loci after meta-analysis. The blue dashed line indicates the threshold for genome-wide significance, *p* = 5 × 10^−8^. SNPs highlighted in red have *p*-value < 5 × 10^−9^. SNPs highlighted in orange have *p*-value < 5 × 10^−8^. **b** Forest plot for the top SNP rs115217673 in Chromosome 1, in *MORN1*. **c** Forest plot for the top SNP rs145274312 in Chromosome 7, near *ASNS*. **d** Forest plot for the top SNP rs113120976 in Chromosome 4, near *PDE5A*. **e** Forest plot for th**e** top SNP rs141421624 in Chromosome 2, in *XPO1*. BP Base Pair, CI Confidence Interval, GWAS Genome Wide Association Study. HR Hazard Ratio. SNP Single Nucleotide Polymorphism.

Four independent loci were significantly associated with progression to H&Y3+ (*p* < 5 x 10^−8^). Regional association plots for all GWAS significant loci are shown in Supplementary Figs. 6-9. The top locus was in Chromosome 1, with lead SNP rs115217673 within the *MORN1* gene (Fig. 2) with 11 SNPs in total in the locus (r^2^ ≥ 0.6 with the independent significant SNP). The top SNP was not a significant eQTL for any genes, according to GTEx, eQTLGen, or MetaBrain databases.

Published summary statistics from a previous progression GWAS by Iwaki et al.^2^ were downloaded from https://pdgenetics.shinyapps.io/pdprogmetagwasbrowser/ (accessed November 2019). Our top SNP in *MORN1* was not associated with progression to H&Y3+ in the Iwaki summary statistics (beta = 0.61, *p* = 0.02). Meta-analysis with the Iwaki summary statistics after excluding overlapping cohorts did not reveal any genome-wide significant loci (see Supplementary Materials).

The second most significant locus was in Chromosome 7, with top SNP rs145274312, nearest to the *ASNS* gene. Only one SNP was included in this locus. This SNP was also not identified as an eQTL for any genes in the eQTL databases. It is important to note that this locus only included one variant with low MAF (1.1%), a wide confidence interval, and without supporting variants in LD (see Supplementary Fig. 9 and LDproxy (https://ldlink.nih.gov). This may be due to the LD patterns in the region as there are no variants in high LD (r2) with the lead variant, however, this locus should be interpreted with caution.

The third most significant locus was in Chromosome 4, with top SNP rs113120976. This was closest to the protein-coding gene *PDE5A*, however it is also near a long non-coding RNA *LOC107986192* (NR_165235.1). The top SNP is a significant cis-eQTL in blood for *USP53* with the effect allele T decreasing gene expression (eQTLGen). However, the SNP was not an eQTL in other tissues as reported in GTEx and MetaBrain. This locus included only two variants (Supplementary Fig. 8) and the lead variant had a low MAF of 1.3% so should also be interpreted with caution.

Finally, there was a locus in Chromosome 2 which was significantly associated with progression to H&Y3+, with top SNP rs141421624. This locus included 9 SNPs spanning several genes: *XPO1, USP34, C2orf74*, and *KIAA1841*, although only the top SNP reached GWAS significance. The lead SNP is intronic in the *XPO1* gene. However, it is also a significant eQTL in blood for the genes *KIAA1841* and *AHSA2* (eQTLGen). It is also a significant eQTL in brain cortex for *C2orf74*, with the effect allele G increasing gene expression (MetaBrain).

In LDproxy (https://analysistools.cancer.gov/LDlink), there were also two missense variants within 500 kb in linkage disequilibrium with the lead SNP rs141421624. One of these is rs1729674, a missense variant in *C2orf74*, with a D’ of 1.0 R2 of 0.014, and MAF of 0.42. The other variant in LD with the lead SNP is rs76248080, a missense variant in the *CCT4*, D’ = 0.55. R^2^ = 0.014, MAF = 0.18. However, both of the missense variants were included in the GWAS meta-analysis and were not significantly associated with H&Y3+ (*p* > 0.05).

Colocalization analysis did not provide strong evidence for shared causal variants between PD H&Y3+ loci and gene expression, with no loci surpassing PP.H4 > 0.75 (Supplementary Table 3, Supplementary Fig. 10).

### Candidate variant analysis

We did not find that any of the 90 PD risk SNPs were associated with PD progression at genome-wide significance (Supplementary Table 4). Two SNPs, representing rare *LRRK2* (rs34637584) and *GBA1* (rs114138760) variants, were not present in our analysis as we filtered out variants with MAF < 1%. Of the 88 PD risk variants tested, we found that only one variant, rs35749011, near *KRTCAP2* and tagging the *GBA1* p.E326K variant, was significantly associated with mortality (*p* = 3.6 x 10^−4^) passing the analysis-wide significance threshold (p-value threshold 0.05/88 = 0.00057). There was also one other variant, rs62333164 in *CLCN3*, which was nominally associated with progression to H&Y3+ (*p* = 0.005) (Supplementary Fig. 11).

We also examined the association between the PD GRS and progression in each cohort, adjusting for sex, age at onset, and PC1-PC5. The random-effects meta-analysis across cohorts did not show an effect of the GRS on mortality (HR = 0.99 [95% CI 0.95 to 1.04], *p* = 0.74), or H&Y3+ (HR = 1.02 [95% CI 0.96 to 1.08], *p* = 0.60).

We also analysed candidate variants that have been previously reported for progression (Supplementary Table 5). One previous study found that rs2242367, within the *SLC2A13* gene and adjacent to the *LRRK2* gene and PD risk locus, was associated with survival in Progressive Supranuclear Palsy (PSP)^12^. In our candidate variant analysis, this SNP rs2242367 was nominally associated with more rapid progression to mortality in PD in a meta-analysis across all cohorts (HR = 1.13 [95% CI 1.04 to 1.21], *p* = 0.002) (Supplementary Table 5). When we looked at association of this SNP in the QSBB cohort alone, where there is pathological confirmation of PD, there was no association between the PSP mortality SNP and PD mortality (HR = 1.13, *p* = 0.22).

### Alzheimer’s Disease Genetic Risk Score (GRS)

To assess whether AD genetic risk outside of *APOE* influences mortality in PD, we created the AD GRS excluding the *APOE* region. In the random-effects meta-analysis, the AD GRS without *APOE* was only nominally associated with mortality (HR = 1.06 [95% CI 1.01 to 1.11], *p* = 0.03).

### Power calculations

The power to detect a signal in a survival GWAS depends on a number of factors, including effect size, allele frequency of the effect allele, and the proportion of individuals meeting the outcome of interest. Using the ‘survSNP’ package^13^, we estimate that this study had 92% power to detect a significant effect (*p* < 5 × 10^−8^) for our top *APOE* SNP rs429358 in the mortality GWAS, given an allele frequency of 16%, Hazard Ratio of 1.34, event/death rate of 32.1% and median time to death of 10.6 years. Supplementary Fig. 12 shows how power changes with different event rates and allele frequencies. Clearly, power for progression studies will increase with longer follow-up as more individuals meet the outcomes.

For the top *MORN1* SNP in the H&Y3 + GWAS, we had 87% power to detect a significant effect, given the allele frequency of 1.6%, Hazard Ratio of 2.76, event rate of 22.6%, and median time to event of 3.1 years. Supplementary Fig. 13 shows power at different event rates and allele frequencies.

## Discussion

We have conducted a large meta-analysis GWAS of progression to clinical milestones in PD. We have identified loci in or near *APOE, TBXAS1, MORN1, ASNS, PDE5A* and *XPO1* as relevant to survival and motor progression in PD. Using this single joint analysis instead of a two-stage replication approach has been shown to be more efficient and provides greater power^14^, and has been adopted by the most recent large-scale PD GWAS studies^1,15–17^. We found that the effects were largely consistent and replicated across cohorts, as illustrated in the forest plots and formal tests of heterogeneity.

The top hit for mortality was the *APOE* SNP rs429358 which tags the ε4 allele. *APOE* is the strongest genetic risk factor for AD^18–21^, and is also associated with cardiovascular disease^22^. In PD, *APOE* is associated with age at onset but this may be more generally related to aging, as the effect of *APOE* was similar in age of entry of controls^23^. Indeed, GWASs of longevity and survival in the general population have identified *APOE* as the strongest genetic factor, with the same ε4 (rs429358) allele associated with increased mortality^24^ and found less frequently in long-living individuals^25^. Thus, our finding that *APOE* is a risk factor for mortality in PD patients may not be specific to PD, as we have only examined all-cause mortality in this study. In addition, it can be difficult to precisely classify the cause of death^26^ as genetic variants could increase the vulnerability of PD patients to other causes of death, such as coronary heart disease. However, we also found that the *APOE* ε4 SNP rs429358 was nominally associated with progression to H&Y3+ (Supplementary Table 6). This could indicate that the *APOE* effect on mortality could be partly explained by motor progression and be PD-specific, since PD-related mortality could be due to falls and overall motor deterioration^27,28^.

We also analysed AD GRSs excluding *APOE*. There was no strong evidence that non-*APOE* AD genetic risk influences mortality in PD. We hypothesize that both dementia^4^ and mortality in PD are largely driven by amyloid-β pathology and plaque formation influenced by *APOE* ε4 genotype, although *APOE* may also contribute via other mechanisms such as immune responses^18^ and Lewy body pathology^29,30^.

*APOE* is also important for cognition and dementia in PD, and potentially motor progression^31^, but not PD risk^1,32^. In a separate GWAS study, we confirmed that *APOE* is a major risk factor for PD dementia^4^, and dementia in PD is predictive of later mortality^33^. Thus it is likely that *APOE* causes more rapid progression in PD as marked by both dementia and mortality, but we did not have the cause of death or cognition data in the majority of our cohorts to determine the extent to which PD dementia contributes to mortality.

The mortality GWAS also identified a locus in *TBXAS1* which encodes a platelet aggregator and vasoconstrictor. This could be due to the influence of genetic factors for all-cause mortality, although these variants in *TBXAS1* were not associated with survival/longevity in the general population^11^. We also identified a locus near *SYT10* which regulates calcium-dependent exocytosis. Syt10 is also important for secretion of insulin-like growth factor-1 which has been suggested to play a role in the development of PD^34^ and deficits in dopamine neuron firing accompanied by motor problems^35^.

In our H&Y3 + GWAS, we identified 4 novel loci associated with progression, near *MORN1, ASNS, XPO1*, and *PDE5A*, although the variants near *ASNS* and *PDE5A* were only covered in two cohorts. In addition, the *ASNS* and *PDE5A* loci included only single variants, and the variants had low MAF (~1%) and wide confidence intervals so should be interpreted with caution. There is a risk that these may be false positives and replication in other cohorts is needed. We have made our summary statistics available at https://tinyurl.com/PDprogressionv2 to encourage replication efforts and enable meta-analysis. Gene ontology analysis did not reveal any biological pathways, gene sets, or tissues that were enriched among the GWAS hits, and this is likely because we are still underpowered for this analysis. A more in-depth discussion of candidate genes is provided in the Supplementary Materials.

There did not appear to be overlap in the hits identified in the mortality and H&Y3+ GWASs (Supplementary Materials). This may be because there are differences in the genetic variation contributing to different forms of progression. For example, motor and cognitive progression in PD are moderately but not perfectly correlated (*r* = 0.35)^3^ and different PD subtypes show different rates of motor and cognitive impairment/progression^36,37^. Thus is it possible that the different findings from the two GWASs may reflect a divergence in the genetic underpinnings for different types of progression. Alternatively, it could reflect the incomplete sample overlap and different sample sizes between the two GWASs.

In line with previous PD progression GWASs, the majority of the 90 PD risk SNPs were not associated with PD progression^2,3,5,38^. Although *LRRK2* G2019S has been previously associated with slower progression^39^, this variant did not meet our allele frequency filter. Overall, the lack of association of risk variants with progression is an important finding in itself which has also been replicated in other PD progression GWASs^2,3,5,38^, and suggests that other factors and pathways may influence progression after disease onset, e.g. amyloid pathology appearing at later disease stages.

We showed that one variant, rs35749011, linked to *GBA1* p.E326K (also known as p.E365K) was associated with mortality. Interestingly this variant does not cause Gaucher disease or have a major effect on glucosylceramide levels suggesting a dissociation between glucosylceramide and the role of *GBA1* in PD progression. This is consistent with the recently reported trial data reporting a lack of effect of the glucosylceramide synthase inhibitor venglustat in modifying PD progression^40^, although we only examined common *GBA1* variants in this study and rarer variants may have a larger effect.

We were not able to replicate findings for other candidate variants nominated from previous PD progression GWASs. We examined results for rs382940 in *SLC44A1* for progression to H&Y3 + 2, however, this was not associated with progression in any of our results.

We also did not identify a mortality or motor progression effect for variants in *RIMS2*, *WWOX*, and *TMEM108* which have been reported for PD dementia^5^. The p-values for these variants were all > 0.3 in our GWASs (Supplementary Table 5) although we did not analyse cognitive impairment or dementia in this study. Overall, our study has identified novel associations and largely not replicated previous GWAS progression findings apart from *APOE*^2–5^. Part of this may be due to the different measures of PD progression (e.g. early and late-stage motor progression, cognitive progression, mortality, and dementia). It is likely that different forms of PD progression are influenced by different genetic variants and processes, although there may be some shared factors such as *APOE*. Furthermore, the heterogeneity of the cohorts (e.g. disease stage, inclusion criteria), in addition to the difficulty in measuring progression through clinical scales, may explain why we have not replicated previous findings even for the same outcome measures as previous studies, namely H&Y3+. We have shown replication of effects across our cohorts, however, replication in independent cohorts of all genetic loci reported in our study and previous studies is necessary.

We did not find evidence to support *APOE* ε2 and *MAPT* H1 haplotype as factors for mortality. We found some evidence suggesting the PSP mortality SNP, rs2242367^12^, was also associated with more rapid mortality in PD. This finding could indicate that there is some misclassification of PSP cases in our PD cohorts, as PSP can be frequently misdiagnosed as PD and we did not have pathological diagnosis data on the majority of cases. We found no association between this SNP rs2242367 and PD mortality in the QSBB cohort, where there is pathological confirmation of PD – thus we cannot rule out the possibility that there is ‘contamination’ of PSP cases in the other cohorts which do not have pathological diagnoses available. Alternatively, the regulatory effects of this SNP, which is separate from the *LRRK2* PD risk locus, may influence survival in both PD and PSP.

This study is one of the largest GWASs of PD progression and the first large-scale GWAS of PD mortality. However, larger sample sizes and longer follow-ups are needed to detect variants with smaller effects (e.g. HR < 1.2) or lower allele frequencies. Due to our limited sample size, we are still underpowered to detect some loci influencing disease progression, particularly in the H&Y3 + GWAS which had a smaller sample size. Further replication efforts are needed in independent cohorts from other parts of Europe and North America. In addition, given the low MAF of some of the top variants identified particularly in the H&Y3 + GWAS, rare variant and burden analysis in sequencing data would be useful to evaluate the impact of rare variants in candidate genes. This was not feasible in the current study as the majority of cohorts only had SNP array data.

We also removed SNPs with heterogeneous effects across cohorts, following previous studies^2^, and to ensure reproducible and robust results, with the aim to remove SNPs that may be false positive results. It is possible that some true progression SNPs have been excluded with this conservative approach.

A second key limitation is that our findings are derived only from individuals of European ancestry and may not extend to individuals from other populations. It has been shown that PD genetic risk factors can differ or have heterogeneous effects in different ancestries^15–17^, and this may also be the case with PD progression variants.

Thirdly, more data is needed on post-mortem pathological diagnosis to conduct cause-of-death analyses, as some of the mortality variants may relate to general population mortality rather than having a specific effect on PD progression. However, *APOE* is the only gene identified in our mortality GWAS that overlaps with general population longevity GWAS. Another limitation is the heterogeneity between cohorts and PD case selection. Our cohorts tend to be recruited from specialist clinics and groups of patients, and this may lead to a tendency to recruit more atypical patients, or rapidly progressing patients. More population-based studies are needed to improve generalisability of these results. Several of our cohorts are also non-incident, with a delay between symptom onset and study entry, and this means that we are not able to capture the most rapidly progressing patients.

In addition, we nominated genes from the top SNPs based on physical proximity and eQTL databases, however, additional fine-mapping and annotation are needed to prioritise causal variants and genes for each locus. More in-depth polygenic risk score analysis, including SNPs below genome-wide significance, could also be performed to examine the overlap between PD risk, progression, AD, and other phenotypes however we were not sufficiently powered to conduct in-depth permutation testing of p-value thresholds in this study. Finally, the interpretation of GWAS for neurological disease remains limited by the resolution of the effects of genomic variants on gene expression from bulk RNA sequencing studies. Rapidly increasing sample sizes, and the development of single-cell resources will enable a more direct interpretation of the relationship between genomic variants and disease biology.

One major challenge is that clinically, motor progression is heterogeneous and influenced by multiple factors, such as the presence and severity of non-motor symptoms, genetic variation, medication, and comorbidities such as Type 2 Diabetes. The aim of the current study was to identify genetic determinants and understand more about the biology of PD progression, thus we did not adjust for other factors that may influence progression as this may be overcorrection. It is possible that some of these factors may lie on the causal pathway between genotype and clinical progression as intermediary factors^41^. We also did not have available data in the current cohorts to include these in models. However future studies which aim to predict PD progression could adjust for risk and protective factors for more accurate prediction.

We conducted two large-scale GWASs of PD progression, including the first GWAS of mortality in PD. We identified six significant genome-wide signals, including *TBXAS1*. We also showed that the genetic factors influencing progression in PD are largely different to those influencing PD risk, emphasising the need for further studies of progression. This work will help us to better understand the biology of PD progression and develop new disease-modifying treatments.

## Methods

### Brief methods

We studied 11 cohorts from Europe and America, and included cohorts in each analysis who had sufficient data on the outcomes of interest (see below for further details). Genotyping, quality control, and imputation was performed in each cohort separately but following the same steps. Only variants with high imputation quality scores (INFO/R2 > 0.8) and minor allele frequency (MAF) > 1% were retained for analysis.

We assessed the following two clinical outcomes: mortality, and H&Y3+. H&Y stage 3 is an important clinical milestone and a commonly used outcome to measure motor progression, as it marks the onset of postural instability^42^, usually accompanied by falls. It is associated with a more rapid stage of motor progression and more severe disability^43–45^. Cohorts were excluded if less than 20 individuals met the outcome of interest during the follow-up period, or < 5% of the total cohort size. For the mortality GWAS, all the cohorts were analysed with the exception of the Parkinson’s Progression Markers Initiative (PPMI): Tracking Parkinson’s, Oxford Discovery, Queen Square Brain Bank (QSBB), CamPaIGN, Cambridge PD cohort, UK Biobank incident cases, UK Biobank prevalent cases, Drug Interaction With Genes in Parkinson’s disease (DIGPD), Trondheim, and Oslo. The PPMI cohort was excluded from the mortality GWAS as less than 20 deaths were recorded in the data download (14/08/2019). For the GWAS of H&Y3+, we analysed cohorts which had this data available: Tracking Parkinson’s, Oxford Discovery, PPMI, DIGPD, and Oslo.

Progression to the clinical milestones was analysed using Cox proportional hazard models. Progression to mortality was assessed from the starting timepoint of PD motor symptom onset, except in the UK Biobank cohorts where PD diagnosis was used (see below). Progression to H&Y3+ was assessed from the time of study entry (baseline visit). We used study entry for progression to H&Y3+ as this is the period where participants can be observed and measured, whereas this would not be possible if disease onset was used, particularly since several of our cohorts were not incident cohorts. Individuals who met the outcome of H&Y3+ at study entry were left censored and excluded from analyses. In each model, we adjusted for age at onset, sex, and the first five genetic principal components to adjust for population stratification. Meta-analysis was performed in METAL (version 2011-03-25)^46^, using an inverse variance weighted fixed effects model. GWASs with a genomic inflation factor above 1.2 were excluded from the meta-analysis. Only SNPs that were genotyped/imputed in > 1000 individuals across all cohorts were included in the final results. SNPs with heterogeneous effects across cohorts were also excluded (*p*-value < 0.05 for Cochran’s Q-test for heterogeneity, and/or I^2^ > 80). The null hypothesis was tested with the standard GWAS significance level of 5 x 10^-8^. Results were uploaded to Functional Mapping and Annotation of GWAS (FUMA; https://fuma.ctglab.nl/)^7^ to annotate, prioritise, and visualize GWAS results, and perform gene-set analysis with MAGMA. Genome-wide Complex Trait Analysis conditional and joint analysis (GCTA-COJO, version 1.94.1) was used to identify independently-associated SNPs^47^, using all individuals in the Accelerating Medicines Partnership Parkinson’s disease (AMP-PD) whole genome sequencing dataset as a reference sample (N = 9422, including PPMI). Fine-mapping of the top loci was performed with Probabilistic Annotation INtegraTOR (PAINTOR) (https://bogdan.dgsom.ucla.edu/pages/paintor/) following the recommended pipeline, to prioritize causal variants and integrate functional genomic data^48–50^. Forest plots for the top SNPs were generated in R v3.6 using the forestplot package, enabling us to determine consistency, and replication of survival risk alleles across cohorts. Further details are provided below.

We also performed candidate variant analysis of the 90 PD risk variants from the most recent PD case-control GWAS^1^, and the cumulative PD genetic risk score (GRS). The GRS was created using only the 90 independent genome-wide significant variants from Nalls et al. ^1^, without any further clumping and thresholding. We also examined associations for other candidate variants that have been reported in PD and Progressive Supranuclear Palsy (PSP) progression: *SLC44A1*^2^, *RIMS2*, *WWOX*, *TMEM108*^5^, APOE ε2 allele, MAPT H1 haplotype, and rs2242367 adjacent to the LRRK2 locus^12^. We analysed the Alzheimer’s disease (AD) GRS in relation to PD progression. 38 loci passing genome-wide significance from the latest AD GWAS were used to create the AD GRS^19^. The APOE region was excluded from the GRS (hg19/GRCh37 coordinates 19:40,000,000-50,000,000)^19^.

### Cohorts

We studied 12 cohorts from Europe and America: Tracking Parkinson’s^51^ (ClinicalTrials.gov identifier NCT02881099), Oxford Discovery^52^, Parkinson’s Progression Markers Initiative (PPMI; NCT01141023)^53^, Queen Square Brain Bank (QSBB) pathologically-confirmed PD cases, Calypso^41,54^, UK Biobank incident cases and UK Biobank prevalent cases (see below for details), Cambridgeshire Parkinson’s Incidence from GP to Neurologist (CamPaIGN)^55,56^, the Cambridge PD Research Clinic cohort^32,57^, Drug Interaction With Genes in Parkinson’s Disease (DIGPD; NCT01564992)^58^, the Trondheim Parkinson’s Disease study (Trondheim)^59^, and the Oslo Parkinson’s Disease study (Oslo)^60^. All participants provided written informed consent. A brief summary of key cohort characteristics and inclusion/exclusion criteria are provided below.

Cohorts were excluded if less than 20 individuals met the outcome of interest during the follow-up period, or < 5% of the total cohort size. Small numbers can produce unreliable effect size estimates and extremely wide confidence intervals. All cohorts were included for analysis of mortality, with the exception of the PPMI study as not enough patients met the outcome. For the analysis of other clinical outcomes, not all cohorts had available clinical assessments. For Hoehn and Yahr stage, we analysed data from Tracking Parkinson’s, Oxford Discovery, PPMI, DIGPD, and Oslo.

If data was available within a cohort, participants who were known to be re-diagnosed with a non-PD condition were excluded from analyses.

### Tracking Parkinson’s

Tracking Parkinson’s is a longitudinal, prospective, multi-centre observational study in the UK^51^. Patients were recruited at 72 secondary healthcare centres that are part of the UK National Health Service. Participants were required to have a clinical diagnosis of PD according to Queen Square Brain Bank criteria and supported by neuroimaging if the clinical diagnosis was uncertain. PD participants were also required to be aged between 18 and 90 years, and be diagnosed with PD within 3.5 years of recruitment. Both drug-naive and treated patients were eligible. Patients were assessed at 6-month intervals with a standardised battery of clinical assessments every 18 months. Participants were excluded if they had severe comorbid illness that interfered with participation in clinical visits, other degenerative forms of parkinsonism (e.g. progressive supranuclear parkinsonism), or parkinsonism due to significant cerebrovascular disease.

### Oxford Discovery

Oxford Discovery is a prospective, longitudinal study led by the Oxford Parkinson Disease Centre^52^. Participants with early idiopathic PD were recruited from neurology clinics across the Thames Valley area in the UK. Participants were required to be diagnosed with PD within the last 3 years according to the UK PD Brain Bank criteria by a neurologist or geriatrician with a PD special interest. Participants were excluded if they had non-idiopathic parkinsonism, dementia before PD by 1 year suggesting Dementia with Lewy Bodies, or cognitive impairment that prevented them from providing informed consent. Participants are assessed every 18 months.

### PPMI

The Parkinson’s Progression Markers Initiative (PPMI) study is a longitudinal, observational study of early PD patients at multiple sites across the US and Europe (https://www.ppmi-info.org/)^53^. PD participants were required to have two of the following symptoms: resting tremor, bradykinesia, and rigidity (must have either resting tremor or bradykinesia), or either asymmetric resting tremor or asymmetric bradykinesia. Participants were required to be diagnosed with PD less than 2 years before screening, aged 30 years or older at the time of PD diagnosis, Hoehn and Yahr stage I or II at baseline, confirmation of dopamine transporter deficit from dopamine transporter single photon emission computed tomography (DAT-SPECT) scan and not expected to require PD medication within at least 6 months from baseline. Participants were excluded if they were already taking a PD medication or had taken taken levodopa, dopamine agonists, MAO-B inhibitors or amantadine within 60 days of Baseline, or had ever taken levodopa or dopamine agonists before baseline for more than 60 days in total. Participants were assessed with the full battery of assessments every 12 months. PPMI data was downloaded on 14/08/2019.

### Queen Square Brain Bank

The Queen Square Brain Bank for Neurological Disorders (QSBB) is an archive of brains and tissue from individuals with neurodegenerative disease and neurologically normal controls. The QSBB is based at University College London in London. A request for clinical data and DNA from pathologically-confirmed PD patients was submitted in May 2018.

### Calypso

Calypso is a community-based prevalence study of PD in Cardiff, Wales. Patients were referred by neurologists, geriatricians, and specialty PD nurses, as well as by self referral^54^. Participants were either invited to a research clinic or could remotely complete questionnaires. Participants who attended the clinic were assessed according to the Queen Square Brain Bank diagnostic criteria for PD, whereas remote participants had their clinical notes reviewed to confirm their diagnosis.

### UK Biobank

PD cases were identified from UK Biobank from hospital episode statistics (HES) with an ICD10 code (G20 for PD) in either the primary or secondary position. PD patients were also identified from self-report and death records. UK Biobank data was downloaded on 13/06/2020 (application 46450). PD patients were classified as either prevalent, incident, or undefined, following the ‘Definitions of Parkinson’s Disease and the major causes of Parkinsonism: UK Biobank Phase 1 Outcomes Adjudication’ document (version 1.0, March 2018; http://biobank.ctsu.ox.ac.uk/showcase/showcase/docs/alg_outcome_pdp.pdf). Briefly, prevalent cases were defined as PD patients who had the first PD ICD code date prior to the baseline assessment, or self-reported PD at the baseline assessment. Incident cases were defined as patients with PD detected by HES with the PD ICD code date after the date of baseline assessment. Patients with PD coded in any position in the death register records, but did not have PD in the HES records at any point were also defined as incident cases but these patients were excluded from our analysis. Patients who did not self-report PD at baseline but at a later follow-up visit, and who did not have PD in any HES records or death register records were classified as ‘undefined’. These patients were also excluded from analysis. In our study, we analysed prevalent and incident PD cases separately.

The date of PD diagnosis was defined according to UK Biobank guidelines, using the earliest date of the PD code from HES or self-report. This date of diagnosis was used as a proxy for PD onset in analysis.

Version 2 of the UK Biobank genotype data was used. Quality control and imputation as described below was performed only in the subset of PD cases in the UK Biobank, rather than existing data on the whole cohort.

### CamPaIGN

The Cambridgeshire Parkinson’s Incidence from GP to Neurologist (CamPaIGN) study is an observational, longitudinal study of incident PD patients in the county of Cambridgeshire, UK^55,56^. The study is an unbiased and population-representative incident PD cohort. New cases of parkinsonism between 2000 and 2002 in Cambridgeshire were referred to the study through multiple sources (general practitioners, neurologists, geriatricians, PD specialist nurses, and hospital discharge coding departments). Cases with suspected onset of parkinsonism prior to the study were excluded. For PD participants, the UK Parkinson’s Disease Brain Bank criteria were used to confirm the diagnosis. Patients were followed up annually and assessed with a standardized battery of demographic, disease history, and neurological assessments.

### Cambridge PD research clinic

PD patients were recruited from the PD Research Clinic at the Cambridge Centre for Brain Repair^32,57^. Participants were required to meet the UK Parkinson’s Disease Society Brain Bank diagnostic criteria. Participants completed a comprehensive battery of clinical and neuropsychological tests, the same as used in the CamPaIGN study, on at least one occasion.

### DIGPD

The Drug Interaction With Genes in Parkinson’s disease (DIGPD) study is a longitudinal cohort study in France^58^. Patients with PD meeting the UK Parkinson’s Disease Society Brain Bank criteria were consecutively recruited from 2009 to 2013 in 4 French university hospitals and 4 general hospitals. PD participants were required to have a disease duration of 5 years or less at recruitment. Patients were followed up longitudinally and assessed with standardised clinical assessments and questionnaires every year for 5 years.

### Trondheim

The Trondheim PD cohort is a prospective longitudinal study of PD patients at the Department of Neurology at St. Olav’s Hospital in Trondheim, Norway^59^. Patients were followed from 1997 onwards, with some having been followed since 1980. Sequential new referrals, over the age of 22 years, were referred to the study. The majority of the participants (80%) resided within 50 miles of Trondheim. PD participants were required to have at least two of the three cardinal signs (resting tremor, bradykinesia, and rigidity), improvement through adequate dopaminergic therapy, and the absence of atypical features or other causes of parkinsonism. Participants with atypical disease presentation were excluded, mainly after autopsy. Other than this, there were no other exclusion criteria other than age. Death records were linked to the Norwegian Cause of Death registry and the Cancer Registry of Norway.

### Oslo

The Oslo PD cohort is a cohort of patients recruited from a movement disorders unit at Oslo University Hospital^60^. Participants were required to have a clinical diagnosis of PD by a neurologist. As a large proportion of referrals to the movement disorders unit are for evaluation for advanced treatment options, such as Deep Brain Stimulation, the patients in this study tend to have an earlier age at onset, severe motor fluctuations, good levodopa response and better cognitive function perhaps than other PD cohorts^60^. Clinical data was collected by assessing patients in the outpatient clinic or from retrospective medical records. Participants were assessed when possible during their regular outpatient clinic appointments, so the mean time between assessments in this dataset was 1.7 years. Death records were obtained from the Norwegian National Registry.

### Genotyping quality control and imputation

Genotyping, quality control, and imputation were performed in each cohort separately but following the same steps. Standard quality control procedures were performed in PLINK v1.9 to remove low-quality variants, samples, related individuals, and ancestry outliers. Briefly, individuals with low overall genotyping rates (<98%), related individuals (Identity-By-Descent PIHAT > 0.1), and heterozygosity outliers (>2 standard deviations away from the mean) were removed. Individuals whose clinically reported biological sex did not match the genetically determined sex were also removed.

To remove ancestry outliers, Principal Components Analysis (PCA) was conducted on a linkage-disequilibrium (LD) pruned set of variants (removing SNPs with an r^2^ > 0.05 in a 50 kb sliding window shifting 5 SNPs at a time) after merging with European (CEU) samples from the HapMap 3 reference panel. Individuals who were more than 6 standard deviations away from the mean of any of the first 10 principal components were removed.

Variants were removed if they had a low genotyping rate (< 99%), Hardy-Weinberg Equilibrium p-value < 1 x 10^-5^, or minor allele frequency < 1%.

Following quality control, genotypes from each cohort were imputed separately using the Michigan Imputation Server. All cohorts were imputed to the Haplotype Reference Consortium panel (r1.1). Only variants with high imputation quality scores (INFO/R2 > 0.8) were retained for analysis, and imputation dosages were converted into hard call genotypes.

Related and duplicated individuals across cohorts were identified by merging individual-level genotype data. One individual from each pair of related individuals was removed (PIHAT > 0.1).

### Statistical analysis

We assessed the following clinical outcomes: mortality, and Hoehn and Yahr stage 3 or greater (when postural instability is present).

The time to event was taken as the first visit where the outcome was met. Individuals who were missing data at all timepoints for the assessment were excluded (e.g. if Hoehn and Yahr stage data was missing at all visits, that patient was excluded from the analysis of progression to Hoehn and Yahr stage 3+).

Progression to each clinical milestone from PD onset was assessed using Cox proportional hazard models, adjusting for age at onset, gender, and the first 5 genetic principal components to adjust for population stratification. For mortality, PD onset was used as the starting timepoint. For Hoehn and Yahr stage 3 or greater, the starting timepoint was set as study entry/baseline visit. We report the proportional hazards assumption p-values for each of the top 10 SNPs in each cohort in Supplementary Tables 7 and 8. Analysis was performed in R using the *survival* package.

### Meta-analysis and visualization

Meta-analysis was performed in METAL, using an inverse variance weighted fixed effects model. GWASs with a genomic inflation factor above 1.2 were excluded from the meta-analysis. Genomic control correction was used to adjust the overall alpha error. After meta-analysis, only SNPs that were present in > 1000 individuals were included in the final results. SNPs with heterogeneous effects across cohorts were also excluded (*p*-value < 0.05 for Cochran’s Q-test for heterogeneity, and/or I squared > 80). Variants with MAF variability greater than 15% across the cohorts were also excluded. The null hypothesis was tested with the standard GWAS significance level of 5 x 10^-8^.

Results were uploaded to Functional Mapping and Annotation of GWAS (FUMA; https://fuma.ctglab.nl/)^7^ to annotate, prioritise, and visualize GWAS results. Standard settings were used in FUMA, with the exception of a higher maximum p-value of to identify lead SNPs (5 x 10^-5^) so that we could report nominal associations. eQTL gene mapping using all tissue types was also used, in addition to positional mapping. Gene and gene-set analysis was performed with MAGMA within FUMA. Forest plots were generated in R v3.6 using the *forestplot* package.

### GCTA-COJO

Genome-wide Complex Trait Analysis conditional and joint analysis (GCTA-COJO version 1.94.1,https://yanglab.westlake.edu.cn/software/gcta/#COJO) was used to identify if there were multiple independent SNPs within the same locus^47,61^. It performs a stepwise model selection procedure to select independently associated SNPs^47,61^.

GCTA-COJO requires a reference sample to estimate LD correlations between SNPs. For our reference sample, we used whole genome sequencing data from the Accelerating Medicines Partnership Parkinson’s disease (AMP-PD), including both PD cases and healthy controls. Standard quality control filters were applied to the AMP-PD data, as described previously^4^ and outlined here. Samples were removed if they had a call rate < 98%, excess heterozygosity (> 2 standard deviations from the mean heterozygosity rate), mismatching clinical sex and genetically determined sex from X chromosome heterogeneity, or if they were from related individuals (Identity-By-Descent PIHAT > 0.125). Variants were excluded if they had missingness > 5%, minor allele frequency < 1%, or Hardy-Weinberg Equilibrium *p*-value < 1 x 10^−5^. To remove ancestry outliers, PCA was conducted on a LD-pruned set of variants after merging with CEU + TSI samples from the HapMap 3 reference panel. Individuals who were more than 6 standard deviations away from the mean of any of the first 10 principal components were removed. After all quality control filters had been applied, 9422 individuals were remaining in the AMP-PD dataset.

AMP-PD data was in genome build hg38 and lifted over to hg19/GRCh37 genome build using liftOver (RRID:SCR_018160; https://genome.sph.umich.edu/wiki/LiftOver) to match the build of the GWAS summary statistics.

### Colocalization

We performed colocalization analyses using *Coloc* version 5.1 (https://chr1swallace.github.io/coloc/index.html)^62^. We also used the package *colochelpR* to help prepare datasets for use in *coloc* (https://github.com/RHReynolds/colochelpR, 10.5281/zenodo.5011869)^63^. We used cis-eQTLs in blood from eQTLGen (https://www.eqtlgen.org/cis-eqtls.html)^9^, and cis-eQTLs in the brain from PsychENCODE (http://resource.psychencode.org/)^64^. Both datasets were downloaded on 22/03/2022.

We followed the same method as in Krohn et al. ^65,66^. For colocalization analysis (https://github.com/RHReynolds/RBD-GWAS-analysis/). For each locus, we examined all genes with 1 Mb of a significant locus in the PD mortality GWAS (p < 5 x 10^−8^). Coloc was run using default priors. These are the prior probabilities that any random SNP in the region is associated with trait 1 or trait 2, p_1_ = 10^-4^ and p_2_ = 10^-4^. We used a threshold of p_12_ = 5 x 10^−6^ for the p_12_ prior, which is the probability that a SNP in the region is associated with both traits. Loci with a posterior probability of hypothesis 4 (PP.H4) ≥ 0.75 were considered colocalized due to a single shared causal variant, rather than two distinct causal variants (PP.H3).

### Fine-mapping with Probabilistic Annotation INtegraTOR (PAINTOR)

The top 10 independent variants from each GWAS (Table 2 and Table 3) were selected for statistical fine-mapping with PAINTOR v3.0^48–50^. We followed the recommended pipeline at the PAINTOR v3.0 wiki (https://github.com/gkichaev/PAINTOR_V3.0/wiki) and which has been used in other GWASs^67^. Firstly, a region of 50 kb around the most significant SNP was selected (+/− 25 kb). Z-scores were calculated from the GWAS summary statistics beta effect size and p-value:

$${\rm{Z}}={\rm{sign}}({\rm{Effect\; Size}})\times {\Phi }^{-1}({\rm{p}}/2)$$

where Φ^−1^ is the inverse cumulative distribution function of the normal distribution. Z-scores were calculated in R using the *zsc* function from the *dotgen* package. Secondly, linkage disequilibrium was computed from the 1000 Genomes (Phase 3) reference data for each of the loci. Thirdly, an annotation matrix was created for each locus using all the annotations in the annotation library provided by PAINTOR. Finally, PAINTOR was run on all loci together, on each annotation independently. This was done using the default settings in PAINTOR, which performs approximate inference and enumeration under the assumption of 2 causal variants per locus. The fine-mapped variants with posterior probability > 0.9 are reported inSupplementary Tables 9 and 10. However, it is important to consider that statistical fine-mapping methods are limited and may not necessarily identify causal SNPs in all GWAS loci; functional validation is required to confirm candidate variants.

### PD risk SNPs and GRS

We also performed candidate variant analysis of the 90 PD risk SNPs from case-control GWAS^1^, and the PD genetic risk score (GRS). The GRS is a cumulative risk score for each individual created from the sum of the genome-wide significant PD risk alleles weighted by effect size. The GRS was created in PLINK v1.9 using the 90 independent genome-wide significant variants from Nalls et al. ^1^. The standardised risk score was analysed in each cohort for each outcome using Cox proportional hazard models, adjusting for age at onset, sex, and the PC1-PC5. We created and tested the GRS in each cohort separately and then meta-analysed results using random-effects meta-analysis in R using the package *meta*.

### Candidate variant analysis

We also examined associations for other candidate variants that have been implicated in PD progression. Previous large-scale genome-wide association studies have identified variants in *SLC44A1* for progression to Hoehn and Yahr stage 3 or greater^2^, and variants in *RIMS2*, *WWOX*, and *TMEM108* for progression to dementia^5^. We also examined results for rs7412 tagging the *APOE* ε2 allele, rs8070723 tagging the *MAPT* H1 haplotype, and rs2242367 adjacent to the *LRRK2* locus, which was associated with survival in Progressive Supranuclear Palsy (PSP)^12^. We extracted the results for these 7 SNPs from each of our progression GWAS meta-analysis results. We applied Bonferroni correction to adjust for multiple testing for the number of variants tested, with p-value threshold *p* = 0.05/7 = 0.007.

### Longevity GWASs

To help clarify whether our mortality GWAS results were specific to PD mortality or more general mortality/survival, we searched the most recent longevity GWAS^11^. and the GWAS Catalog (https://www.ebi.ac.uk/gwas/). Summary statistics from the Timmers et al. ^11^. Longevity GWAS were downloaded from https://datashare.ed.ac.uk/handle/10283/3599. We searched these summary statistics for the top SNPs and genes (+/− 1 Mb) from our PD mortality GWAS results.

### Ethics

Tracking Parkinson’s: The West of Scotland Research Ethics Service (WoSRES) Research Ethics Committee gave ethical approval for this study (ref 11/AL/0163). Oxford Discovery: NRES Committee, South Central Oxford A Research Ethics Committee gave ethical approval for this study (ref 16/SC/0108). CamPaIGN: Cambridge Research Ethics Committee gave ethical approval for this study. Cambridge PD Research Clinic: Cambridge Research Ethics Committee gave ethical approval for this study. PPMI: The Research Subjects Review Board at the University of Rochester approved the PPMI study protocol. UK Biobank: UK Biobank has approval from the North West Multi-centre Research Ethics Committee (MREC) as a Research Tissue Bank (RTB). QSBB: The London Central Research Ethics Committee gave ethical approval for this research tissue bank (ref 18/LO/0721). DIGPD: The Ethical Committee Ile-De-France VI gave ethical approval for this study (ID project: 2009-A00109-48). Calypso: Wales Research Ethics Committee 3 gave ethical approval for this study. Trondheim: the Ethics Committee of Central Norway gave ethical approval for this study (ref 2011/1137). Oslo: the Regional Committee for Medical Research Ethics in South-East Norway gave ethical approval for this study.

### Supplementary information

Supplementary Materials

## Data Availability

GWAS summary statistics are available for download at: https://tinyurl.com/PDprogressionv2 (10.5281/zenodo.8017385). Tracking Parkinson’s data is available through the Tracking Parkinson’s portal: https://www.trackingparkinsons.org.uk/about-1/data/. The Oxford Parkinson’s Disease Centre Discovery Cohort data (10.1016/j.parkreldis.2013.09.025) are available on request (Michele Hu, michele.hu@ndcn.ox.ac.uk). The Cambridgeshire Parkinson’s Incidence from GP to Neurologist (CamPaIGN) (https://www.thebarkerwilliamsgraylab.co.uk/parkinsons-disease/current-studies-pd/) data and the Cambridge clinic data are available on request (Caroline Williams-Gray/ Roger Barker; chm27@cam.ac.uk). Parkinson’s Progression Markers Initiative (PPMI) data was accessed from the PPMI platform: https://www.ppmi-info.org/access-data-specimens/download-data. Queen Square Brain Bank for Neurological Disorders (QSBB) data are available upon request (qsbbmtas@ucl.ac.uk). UK Biobank data were accessed through the UK Biobank: https://www.ukbiobank.ac.uk/enable-your-research/apply-for-access. Drug Interaction With Genes in Parkinson’s Disease (DIGPD) data (https://clinicaltrials.gov/ct2/show/NCT01564992) are available upon request (Jean-Christophe Corvol, jean-christophe.corvol@aphp.fr). Calypso data are available on request to the study team (Huw Morris, h.morris@ucl.ac.uk). The Trondheim Parkinson’s Disease Study (Trondheim) data are available on request (10.14802/jmd.21029). The Oslo Parkinson’s Disease data are available on request (Lasse Pihlstrom/ Mathias Toft, lasse.pihlstrom@medisin.uio.no).

## Code availability

All code for our analyses is publicly available at https://github.com/manuelatan/PD-survival-GWAS (10.5281/zenodo.7923843).
