# Supplementary material for: Genome-wide determinants of mortality and motor progression in Parkinson’s disease
Source: NPJ Parkinsons Dis. 2024 Jun 7;10:113. doi: 10.1038/s41531-024-00729-8 (PMC11161485; doi:10.1038/s41531-024-00729-8)
Supplement: Supplementary file 1 — Supplementary Materials [file 41531_2024_729_MOESM1_ESM.pdf]

Genome-wide determinants of mortality and motor progression in Parkinson's disease: Supplementary Materials

Table of Contents

SUPPLEMENTARY METHODS **2**
PD risk SNPs *2*
Meta-analysis with Iwaki et al. (2019) H&Y3+ GWAS *2*
Slope-Hunter for collider bias *2*
Mortality vs H&Y3+ *3*
Progression to H&Y4+ and H&Y5 ..... *3*
SUPPLEMENTARY FIGURES **5**
Supplementary Figure 1. ..... *5*
Supplementary Figure 2. ..... *6*
Supplementary Figure 3. ..... *7*
Supplementary Figure 4. ..... *8*
Supplementary Figure 5. ..... *9*
Supplementary Figure 6. ..... *10*
Supplementary Figure 7. ..... *11*
Supplementary Figure 8. ..... *12*
Supplementary Figure 9. ..... *13*
Supplementary Figure 10. ..... *14*
Supplementary Figure 11. ..... *15*
Supplementary Figure 12. ..... *16*
Supplementary Figure 13. ..... *17*
Supplementary Figure 14. ..... *18*
Supplementary Figure 15. ..... *19*
SUPPLEMENTARY TABLES **20**
Supplementary Table 1. ..... *20*
Supplementary Table 2. ..... *21*
Supplementary Table 3. ..... *38*
Supplementary Table 4. ..... *56*
Supplementary Table 5. ..... *59*
Supplementary Table 6. ..... *60*
Supplementary Table 7. ..... *61*
Supplementary Table 8. ..... *62*
Supplementary Table 9. ..... *63*
Supplementary Table 10. ..... *64*
Supplementary Table 11. ..... *65*
Supplementary Table 12. ..... *66*
Supplementary Table 13. ..... *67*
SUPPLEMENTARY DISCUSSION **68**
Potential causal genes *68*
SUPPLEMENTARY NOTES **70**
Software *70*
Public databases *70*
DIGPD study group *71*
AMP PD acknowledgements *72*
AMP PD Cohort Acknowledgements *72*
SUPPLEMENTARY REFERENCES **74**

SUPPLEMENTARY METHODS

PD risk SNPs

As a sensitivity analysis for the candidate variant analysis, we also looked at other variants surrounding the top 90 PD risk SNPs[1], which could potentially contribute via different mechanisms than the lead PD risk SNP. To do this, we conducted an additional sensitivity analysis where we looked at the results for the variants in the progression GWAS which were +/- 1Mb away from the 88 lead PD risk SNPs. We used the Benjamini-Hochberg, or False Discovery Rate (FDR), correction for multiple testing since the variants are in linkage disequilibrium and therefore not independent. Two loci passed the multiple testing threshold with FDR-adjusted p-value < 0.05. The first locus was within *SH3GL2,* with lead SNP rs12554329 associated with more rapid progression to PD mortality, p-value = 1.5×10^{-6}, and within 1Mb of two PD risk loci, rs10756907 and rs13294100. The second locus was within *SAMD4A* with lead SNP rs73273366, which was also associated with more rapid progression, p-value = 7.4×10^{-7}. This locus was near the PD risk locus rs11158026 which is within *GCH1* but spans several other candidate genes including *SAMD4A*. We also performed the same analysis with the H&Y3+ GWAS but there were no loci that passed correction for multiple testing.

Meta-analysis with Iwaki et al. (2019) H&Y3+ GWAS

We performed a meta-analysis of our H&Y3+ summary statistics with the Iwaki et al.[2] H&Y3+ GWAS. There were some overlapping cohorts so we excluded these cohorts from our summary statistics: PPMI, DIGPD and Oslo. There were only 431,601 SNPs in the Iwaki summary statistics and 344,656 that were genotyped in more than 1000 individuals (likely due to the minor allele frequency filter > 5% and heterogeneity filtering), so there were only a small number of SNPs overlapping between both datasets.

After filtering for SNPs genotyped in > 1000 individuals, the top SNP was rs75614365 in *SORBS2*, p-value = 2.12×10^{-8}, however this variant was not present in the Iwaki summary statistics. It was only genotyped in Tracking Parkinson's and Oxford with a total sample size of 2,372. When we filtered for SNPs present in our data and the Iwaki summary statistics, only 300,498 SNPs were present. None of these reached genome-wide significance. The top SNP was rs115217673, which is the same as in our main H&Y3+ meta-analysis (in *MORN1*) but with larger p-value, 2.72×10^{-6}.

Slope-Hunter for collider bias

We performed a sensitivity analysis to assess the impact of collider bias. This occurs when there is selection or conditioning of analysis on a subset of individuals. In a progression GWAS, this occurs because the analysis is just performed in individuals with disease, in our case PD. This can lead to biased estimates. Recently there have been several new methods proposed to estimate and adjust for collider bias, although these need to be more thoroughly validated as they are based on assumptions which cannot

usually be verified from the available data[3]. We used the Slope-Hunter method proposed by Mahmoud et al.[4]. This method aims to identify a set of SNPs which only affect disease risk and not progression, and use this set of SNPs to estimate a ‘correction factor’ which other variant estimates are then adjusted by. In our sensitivity analysis for the mortality GWAS, the adjustment factor estimated using the Slope-Hunter method was 0.320 (95% CI 0.071 to 0.568, based on a standard error of 0.127 using 100 bootstrap samples). The positive adjustment factor suggests that there are common causes of PD risk and PD mortality with discordant net directions of effect. The top 10 SNPs from the SlopeHunter method are reported in Supplementary Table 11. Random pruning is performed to avoid bias from the winner’s curse, so the selected SNPs are not exactly the same as the progression GWAS hits. Some effect estimates were adjusted up and others were adjusted down however they were overall very similar.

Interestingly, we note that the variant rs3574901, tagging *GBA1* p.E326K, was no longer significantly associated with mortality after adjustment with the SlopeHunter method. The variant was nominally associated with mortality in unadjusted analysis (beta = 0.40, SE = 0.11, p = 3.6 x 10^{-4}) however in the adjusted estimates the effect was no longer significant (beta = 0.16, SE = 0.15, p = 0.29). This is an interesting finding which suggests that collider bias may be influencing the results of *GBA1* variants on progression, however this needs to be investigated in other studies.

We also looked at the top 10 mortality SNPs and whether they were associated with age at onset. These results are shown in Supplementary Table 12. Overall the results for the top mortality SNPs were similar even when age at onset was not included as a covariate in the Cox proportional hazards models, although the p-values were slightly larger. The top 10 SNPs were also not associated with age at onset directly in linear models, adjusting for sex and PC1-PC5.

Mortality vs H&Y3+

To investigate whether the genetic loci underlying progression to mortality and H&Y3+ were similar, we performed a sensitivity analysis by conducting the GWASs using just the four cohorts that had both mortality and Hoehn and Yahr data available. We found that the top signals were not shared between the two GWASs (Supplementary Figure 14). Though underpowered, this analysis suggests that the genetic signals underlying progression to mortality and H&Y3+ are largely distinct, although further analysis in larger samples is needed. We also looked at the top mortality SNPs and checked their p-values in the H&Y3+ GWAS, and none were significant except for the *APOE* variant (p = 0.037) (Supplementary Table 6).

Progression to H&Y4+ and H&Y5

As a sensitivity analysis, we analysed the top SNPs from the H&Y3+ GWAS and looked at their associations with progression to H&Y stage 4 or greater, and H&Y stage 5. The ability to analyse H&Y stage 4 (severely disabling disease) and greater is limited by

subject drop out as at this point many subjects will not attend for follow-up research visits. We assessed the top 4 SNPs in the motor progression GWAS against time to H&Y4+ and H&Y5 in each cohort individually and then performed a random-effects meta-analysis. Some of the individual cohort estimates were very imprecise (very large estimates with very wide confidence intervals and large standard error). With rarer variants the Cox proportional hazard models in the individual cohort can be unreliable, and also the random-effects meta-analysis across cohorts were not able to obtain stable results because of the large variance between studies. The results are reported in Supplementary Table 13.

SUPPLEMENTARY FIGURES

Supplementary Figure 1. Kaplan-Meier curve for mortality stratified by cohort. Only cohorts included in the mortality GWAS are shown.

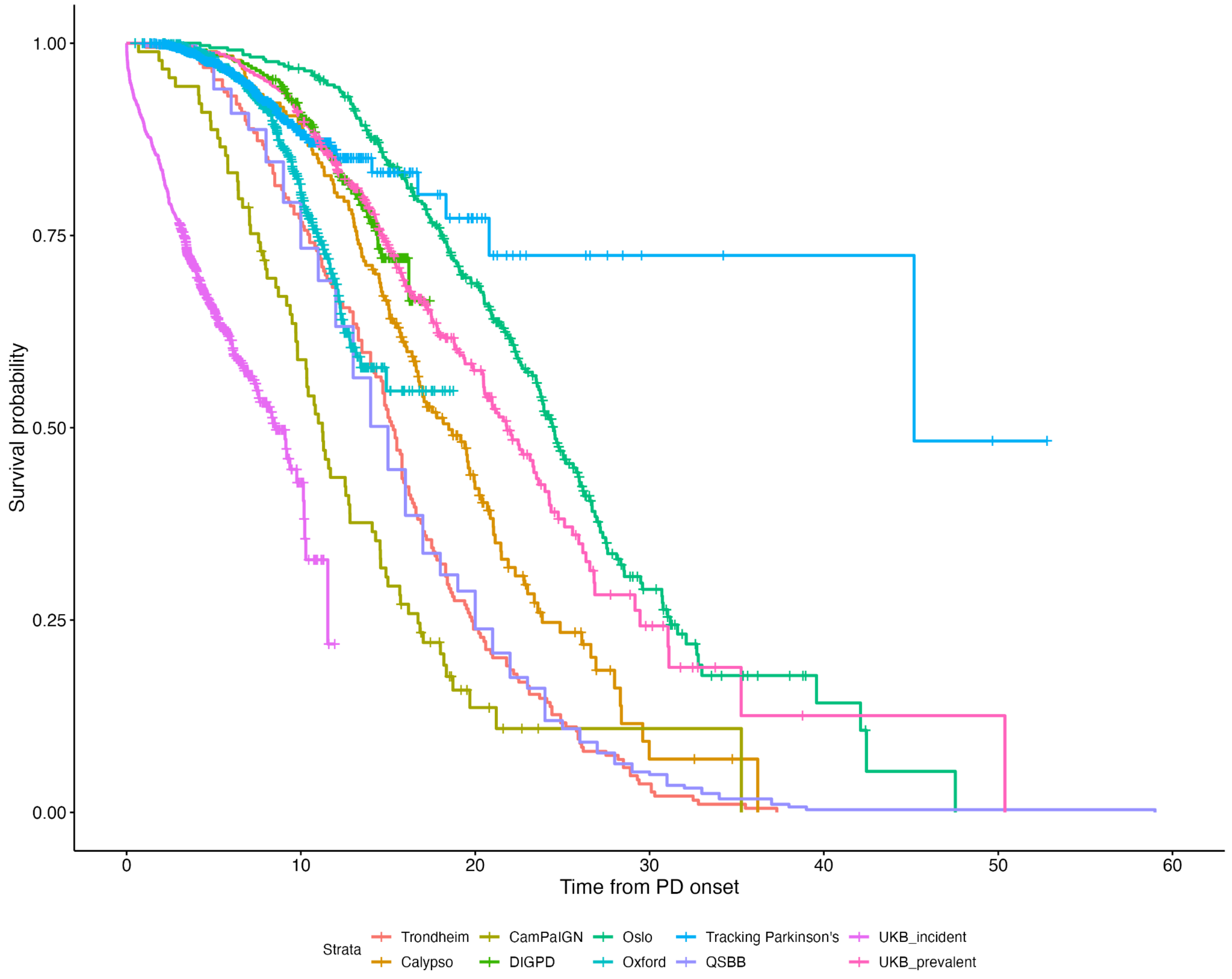

Time from PD onset is shown in years. Vertical lines indicate censoring.

Supplementary Figure 2. LocusZoom plot for rs429358 in mortality GWAS in Chromosome 19.

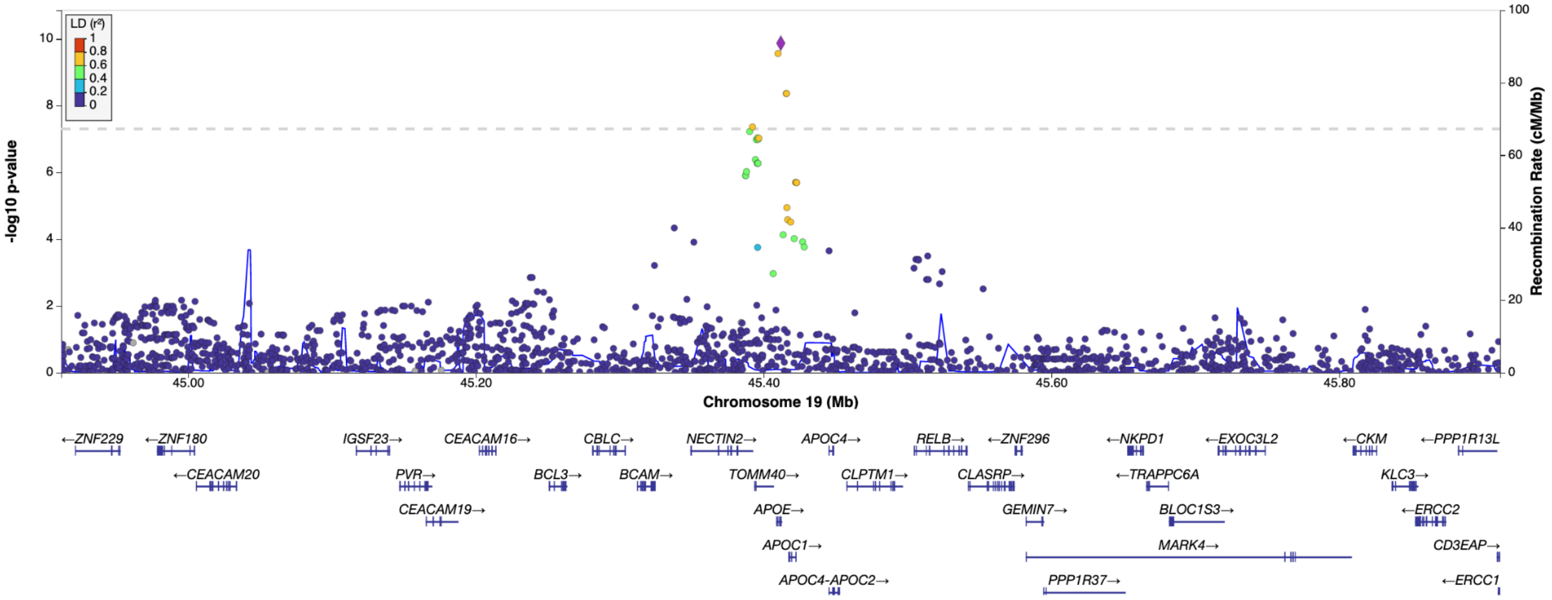

Data is shown in hg37 build. The purple diamond shows the most significantly associated Single Nucleotide Polymorphism (SNP) in the locus. SNPs in linkage disequilibrium (LD) with the index SNP are shown in coloured circles according to the r^2, as shown in the legend on the top left. The recombination rate is shown as the blue line with the axis on the right y axis. Genes are shown in the bottom panel with the positions of exons displayed as bars, and transcribed strand indicated with an arrow.

Supplementary Figure 3. LocusZoom plot for rs4726467 in mortality GWAS in Chromosome 7.

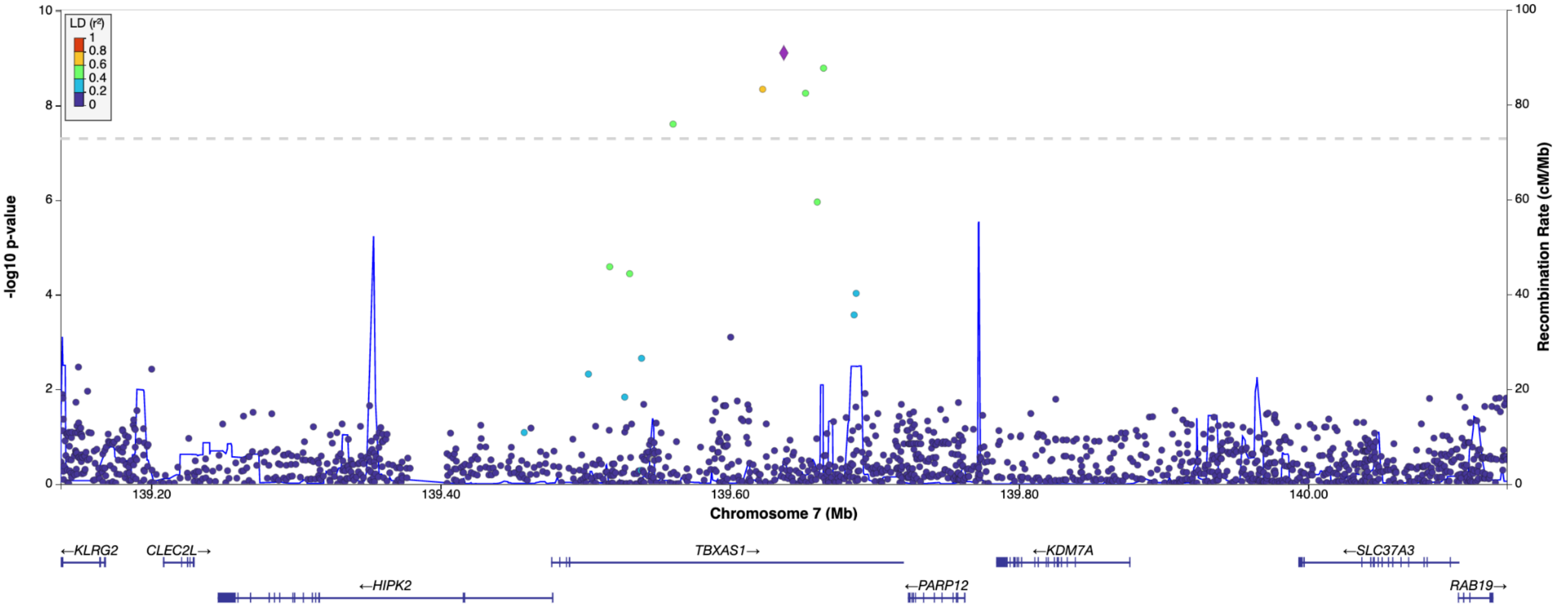

Data is shown in hg37 build. The purple diamond shows the most significantly associated Single Nucleotide Polymorphism (SNP) in the locus. SNPs in linkage disequilibrium (LD) with the index SNP are shown in coloured circles according to the r^2, as shown in the legend on the top left. The recombination rate is shown as the blue line with the axis on the right y axis. Genes are shown in the bottom panel with the positions of exons displayed as bars, and transcribed strand indicated with an arrow.

Supplementary Figure 4. LocusZoom plot for rs10437796 in mortality GWAS in Chromosome 12.

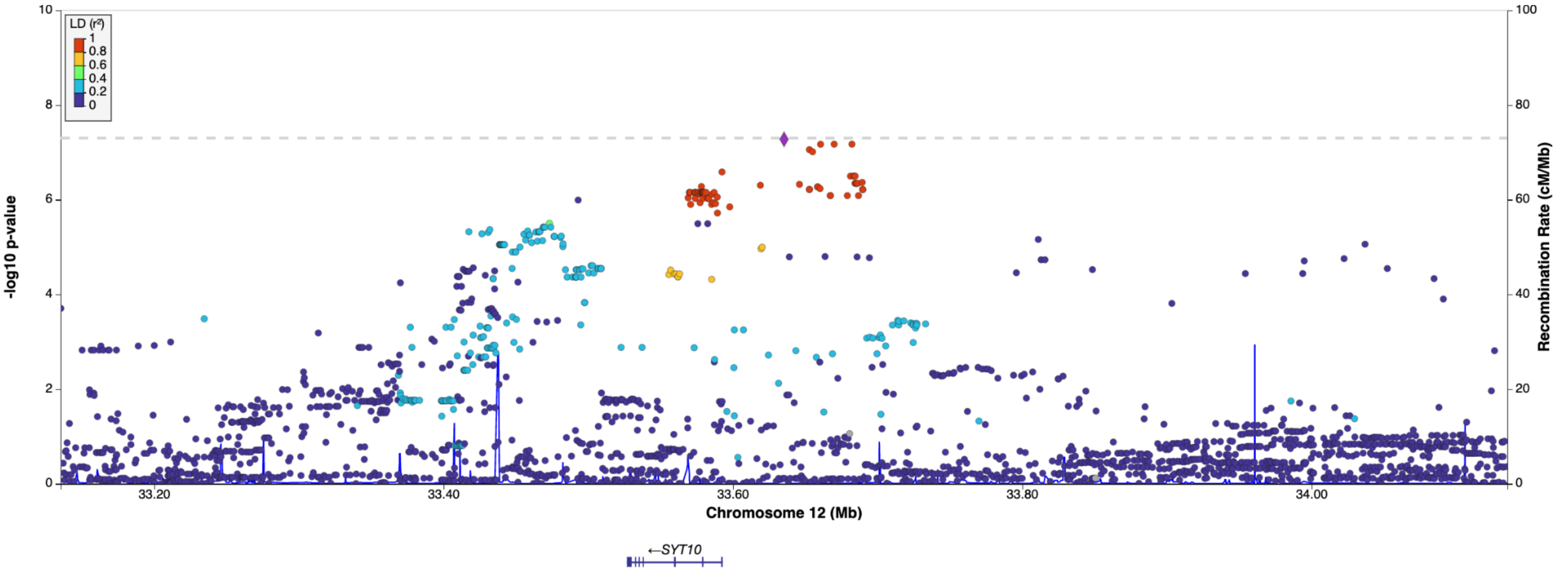

Data is shown in hg37 build. The purple diamond shows the most significantly associated Single Nucleotide Polymorphism (SNP) in the locus. SNPs in linkage disequilibrium (LD) with the index SNP are shown in coloured circles according to the r^2, as shown in the legend on the top left. The recombination rate is shown as the blue line with the axis on the right y axis. Genes are shown in the bottom panel with the positions of exons displayed as bars, and transcribed strand indicated with an arrow.

Supplementary Figure 5. Kaplan-Meier curve for H&Y3 stratified by cohort. Only cohorts included in the H&Y3 GWAS are shown.

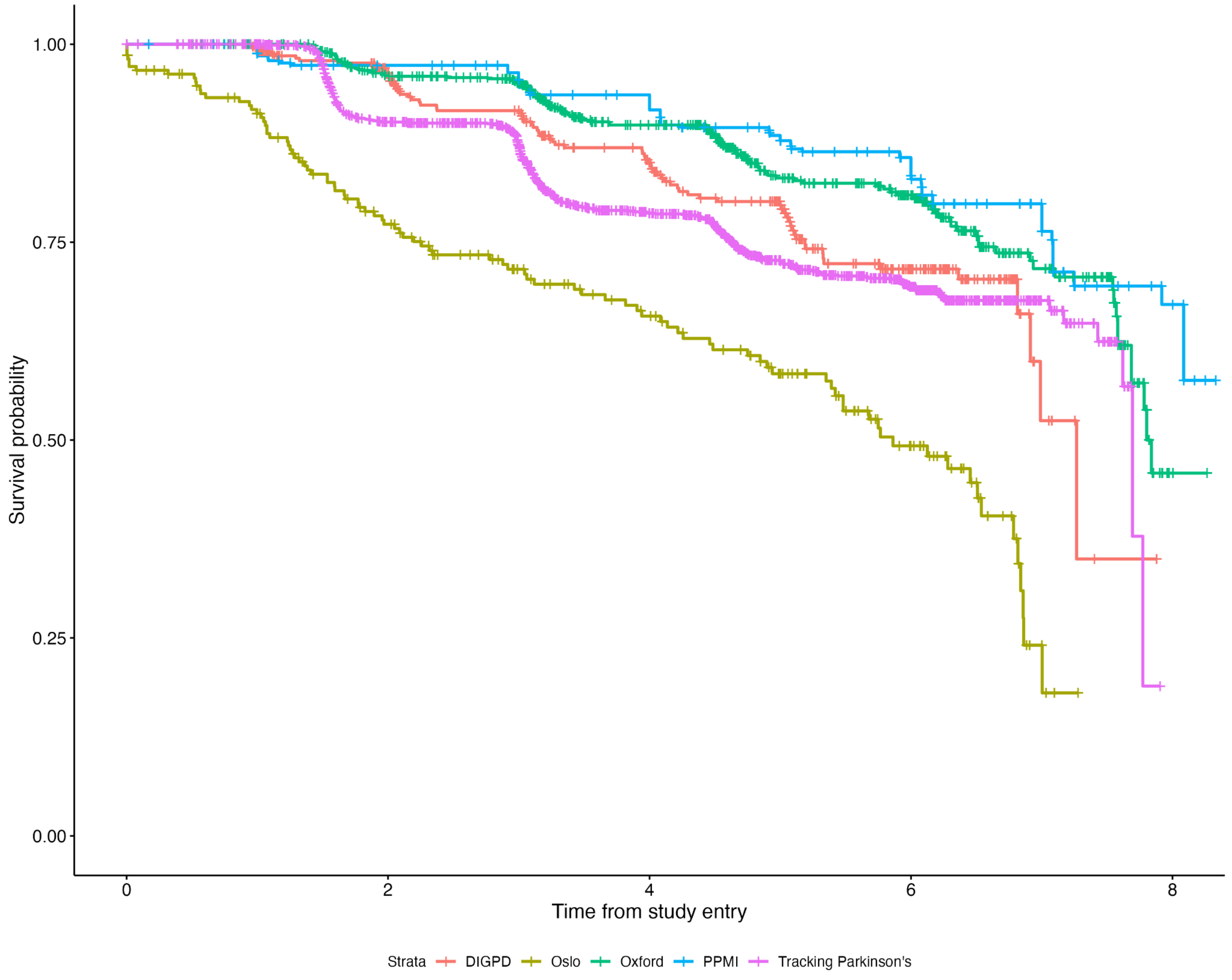

Time from study entry is shown in years. Vertical lines indicate censoring.

Supplementary Figure 6. LocusZoom plot for rs115217673 in H&Y3+ GWAS in Chromosome 1.

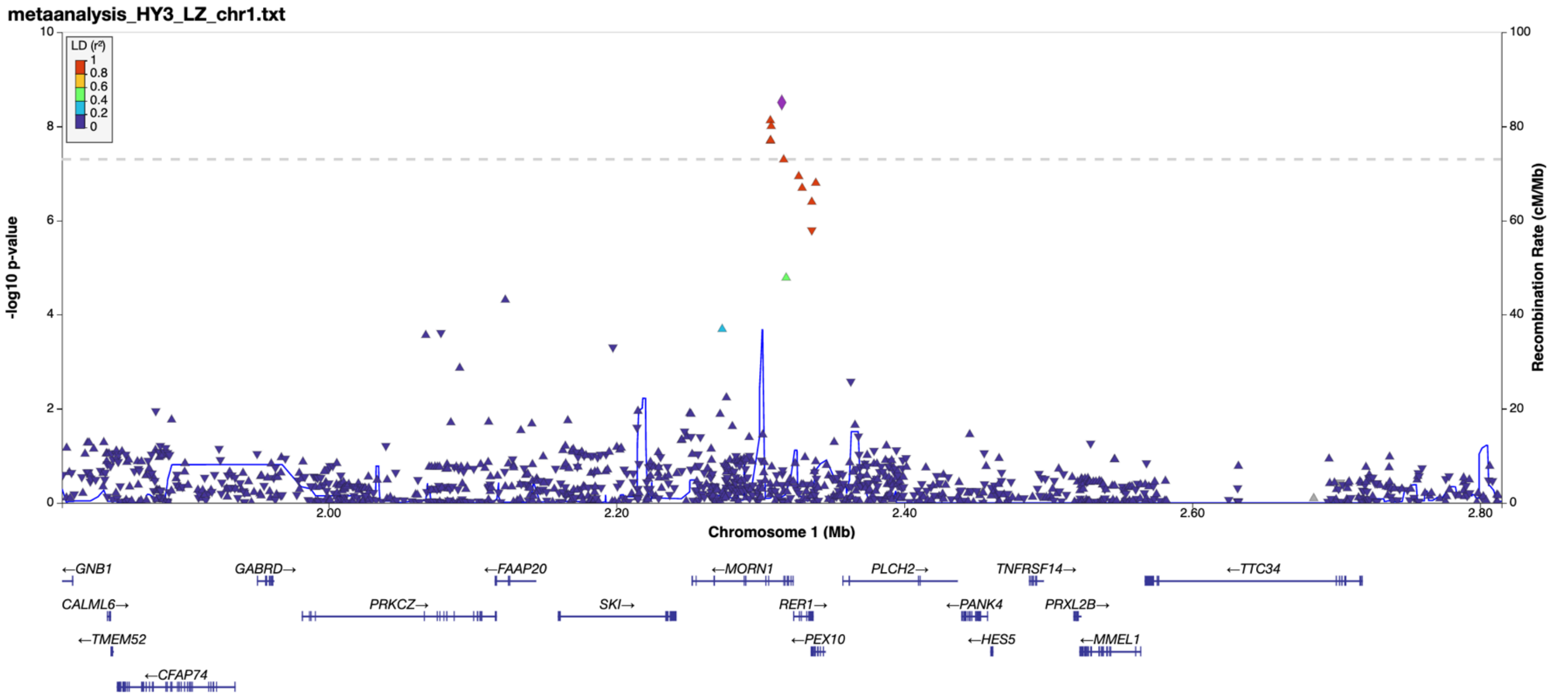

Data is shown in hg37 build. The purple diamond shows the most significantly associated Single Nucleotide Polymorphism (SNP) in the locus. SNPs in linkage disequilibrium (LD) with the index SNP are shown in coloured circles/triangles according to the r^2, as shown in the legend on the top left. The recombination rate is shown as the blue line with the axis on the right y axis. Genes are shown in the bottom panel with the positions of exons displayed as bars, and transcribed strand indicated with an arrow.

Supplementary Figure 7. LocusZoom plot for rs141421624 in H&Y3+ GWAS in Chromosome 2.

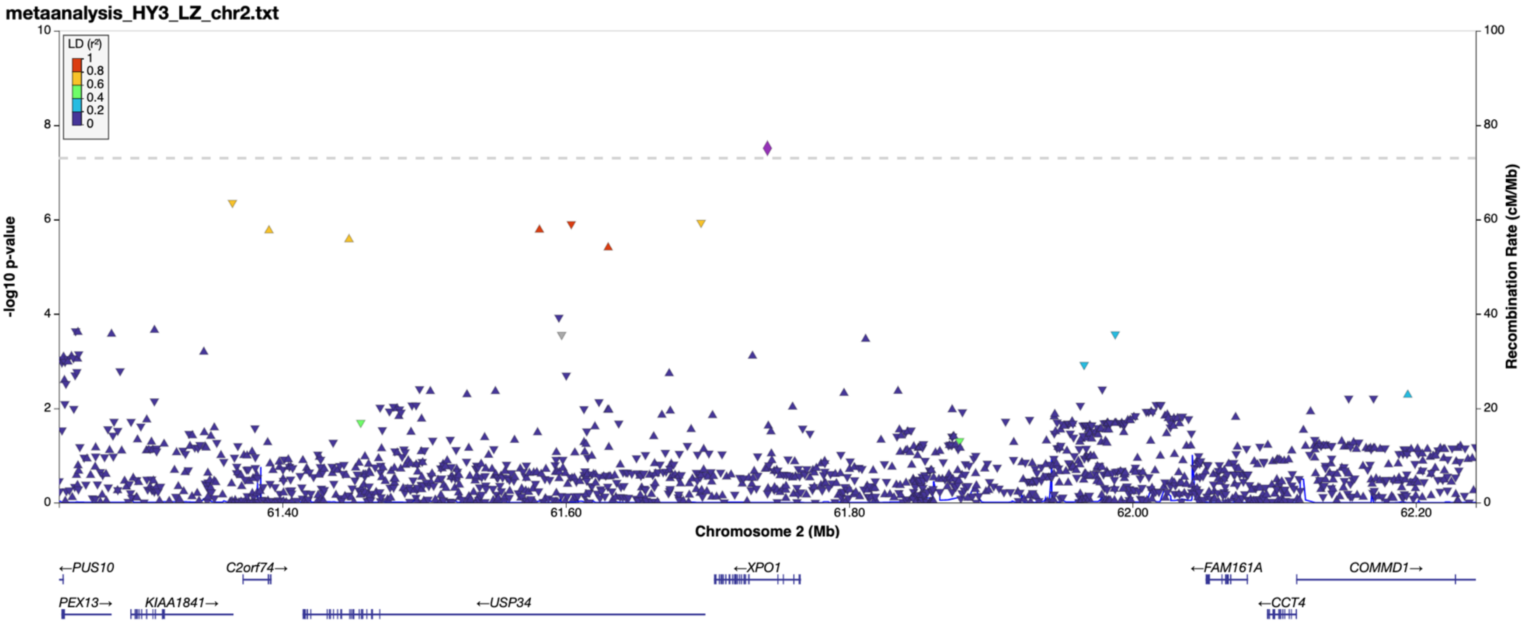

Data is shown in hg37 build. The purple diamond shows the most significantly associated Single Nucleotide Polymorphism (SNP) in the locus. SNPs in linkage disequilibrium (LD) with the index SNP are shown in coloured circles/triangles according to the r^2, as shown in the legend on the top left. The recombination rate is shown as the blue line with the axis on the right y axis. Genes are shown in the bottom panel with the positions of exons displayed as bars, and transcribed strand indicated with an arrow.

Supplementary Figure 8. LocusZoom plot for rs113120976 in H&Y3+ GWAS in Chromosome 4.

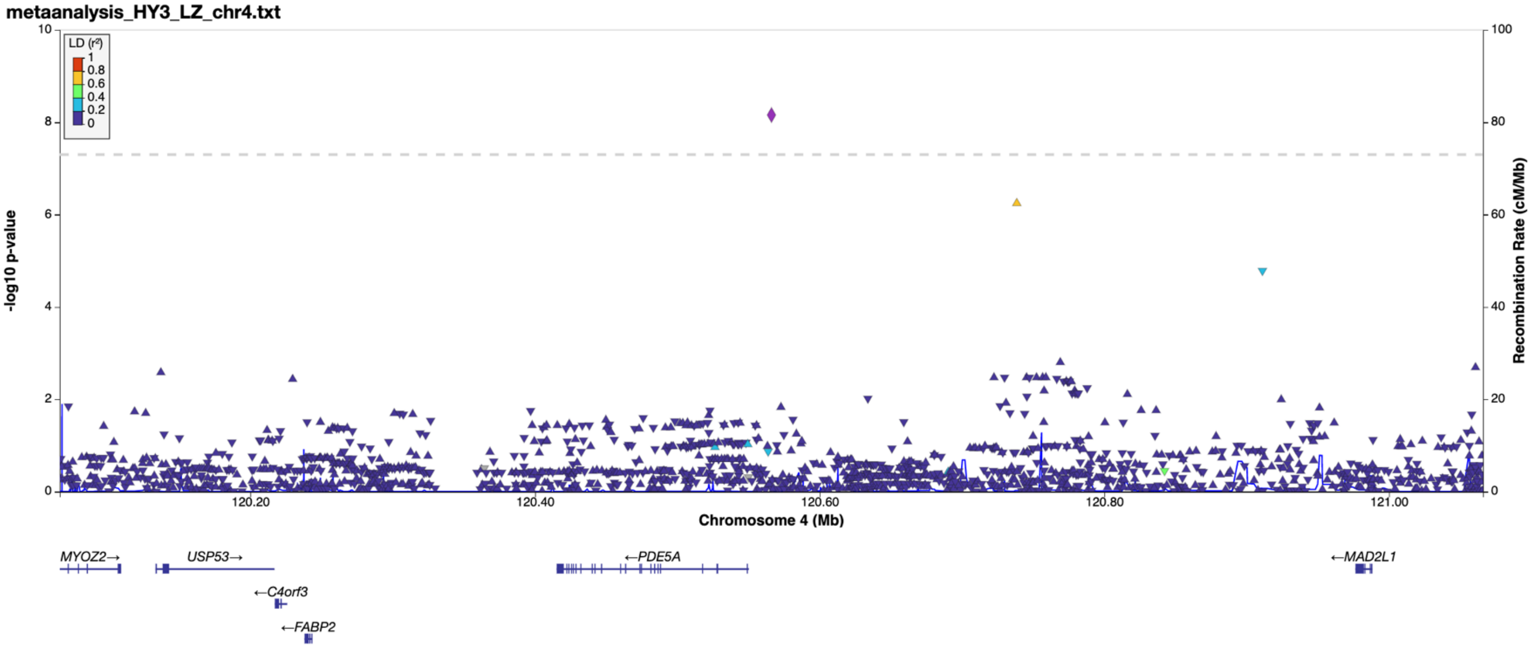

Data is shown in hg37 build. The purple diamond shows the most significantly associated Single Nucleotide Polymorphism (SNP) in the locus. SNPs in linkage disequilibrium (LD) with the index SNP are shown in coloured circles/triangles according to the r^2, as shown in the legend on the top left. The recombination rate is shown as the blue line with the axis on the right y axis. Genes are shown in the bottom panel with the positions of exons displayed as bars, and transcribed strand indicated with an arrow.

Supplementary Figure 9. LocusZoom plot for rs145274312 in H&Y3+ GWAS in Chromosome 7.

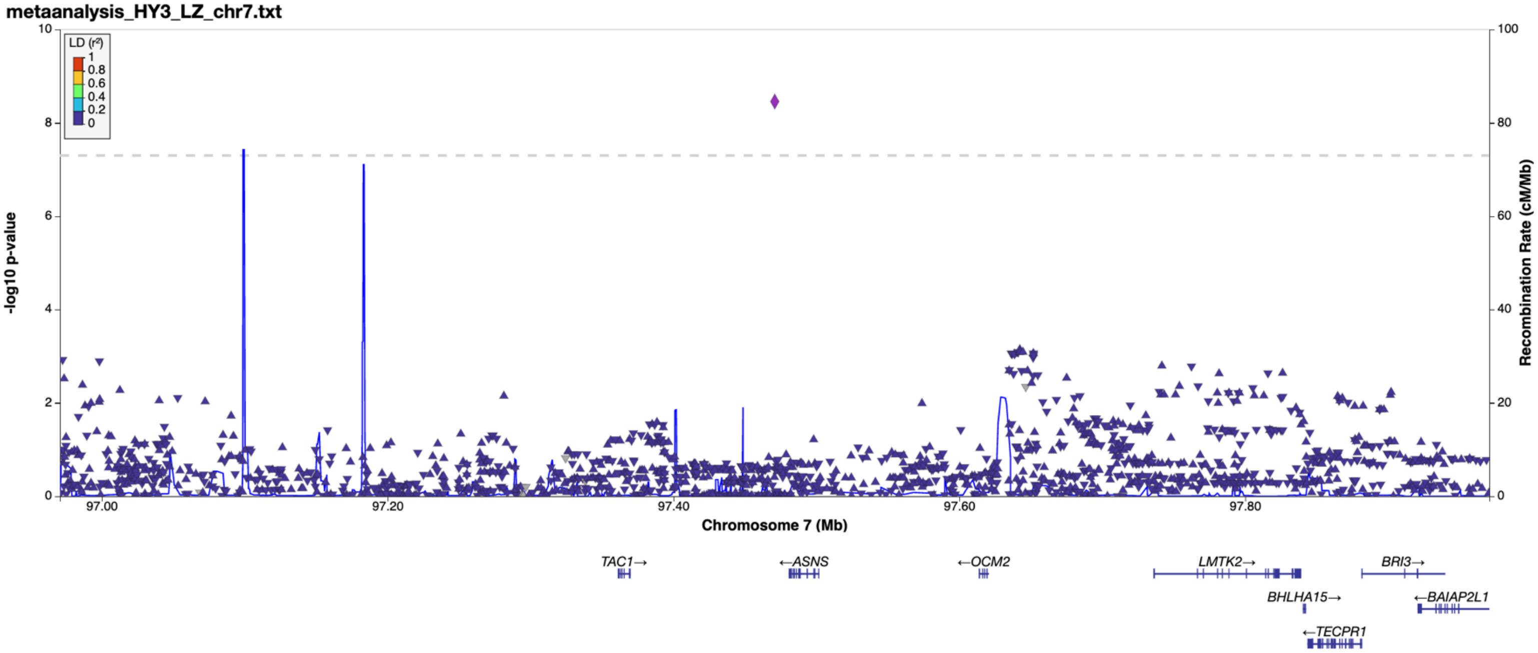

Data is shown in hg37 build. The purple diamond shows the most significantly associated Single Nucleotide Polymorphism (SNP) in the locus. SNPs in linkage disequilibrium (LD) with the index SNP are shown in coloured circles/triangles according to the r^2, as shown in the legend on the top left. The recombination rate is shown as the blue line with the axis on the right y axis. Genes are shown in the bottom panel with the positions of exons displayed as bars, and transcribed strand indicated with an arrow.

Supplementary Figure 10. Regional association plot for eQTL (derived from PsychENCODE) and PD H&Y3 GWAS colocalizations in the region surrounding *PUS10*.

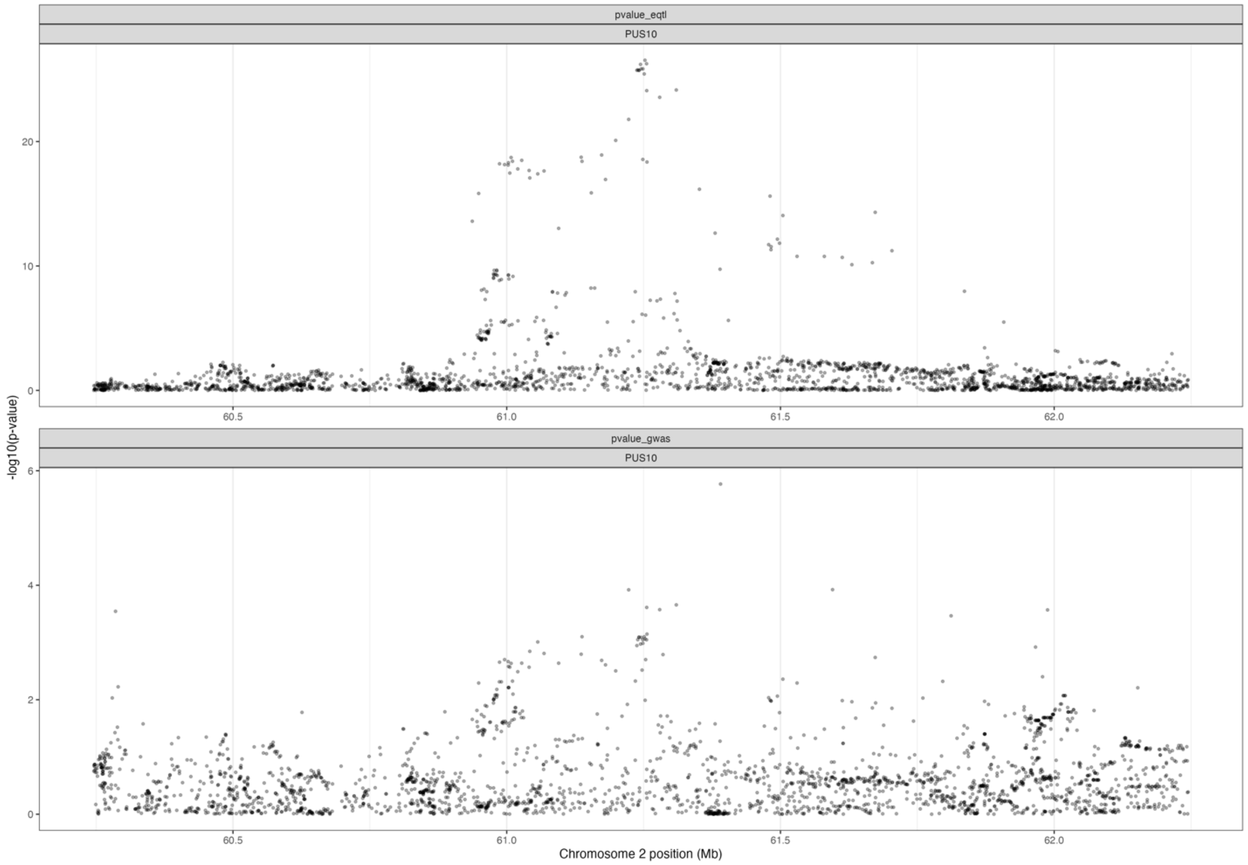

Regional association plot for eQTL and PD H&Y3 GWAS colocalization in the region surrounding *PUS10*. The regional association plot for eQTL are shown in the upper pane, and the PD H&Y3 GWAS association signals are shown in the lower pane. eQTLs were derived from the PsychENCODE, where adult brain tissue from 1,387 individuals was analysed. The PP.H4 (the posterior probability that there is an association in the region with trait 1 and trait 2, with one shared SNP) for *PUS10* was 0.698. The x-axis denotes chromosomal position in hg19, and the y-axis indicates association p-values on a -log10 scale.

Supplementary Figure 11. Heatmap showing the PD GWAS risk loci from Nalls et al. (2019) that were associated ($p < 0.05$) with mortality, or H&Y stage 3+. Only variants with at least one association $p < 0.05$ are shown in the heatmap.

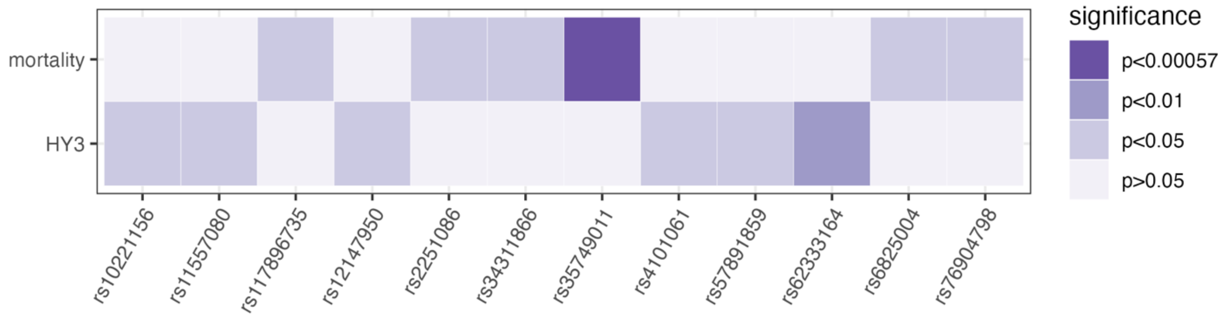

Supplementary Figure 12. Power calculations for mortality GWAS with N = 5,744 at different effect sizes, allele frequencies, and event rates (shown in faceted plots with grey bars). Calculated using the R package 'survSNP'.

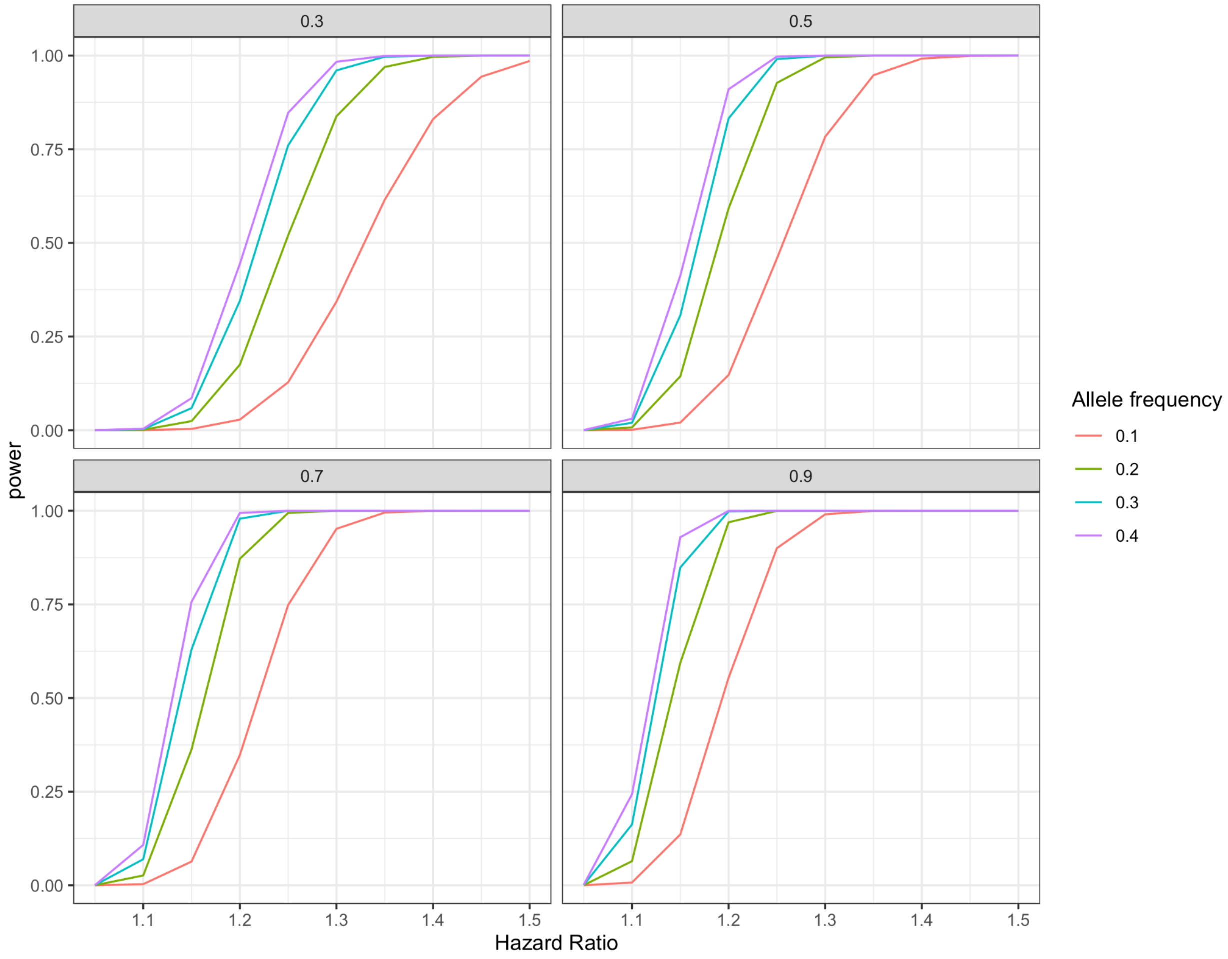

Supplementary Figure 13. Power calculations for H&Y3+ GWAS with N = 3,331 at different effect sizes, allele frequencies, and event rates (shown in faceted plots with grey bars). Calculated using the R package ‘survSNP’.

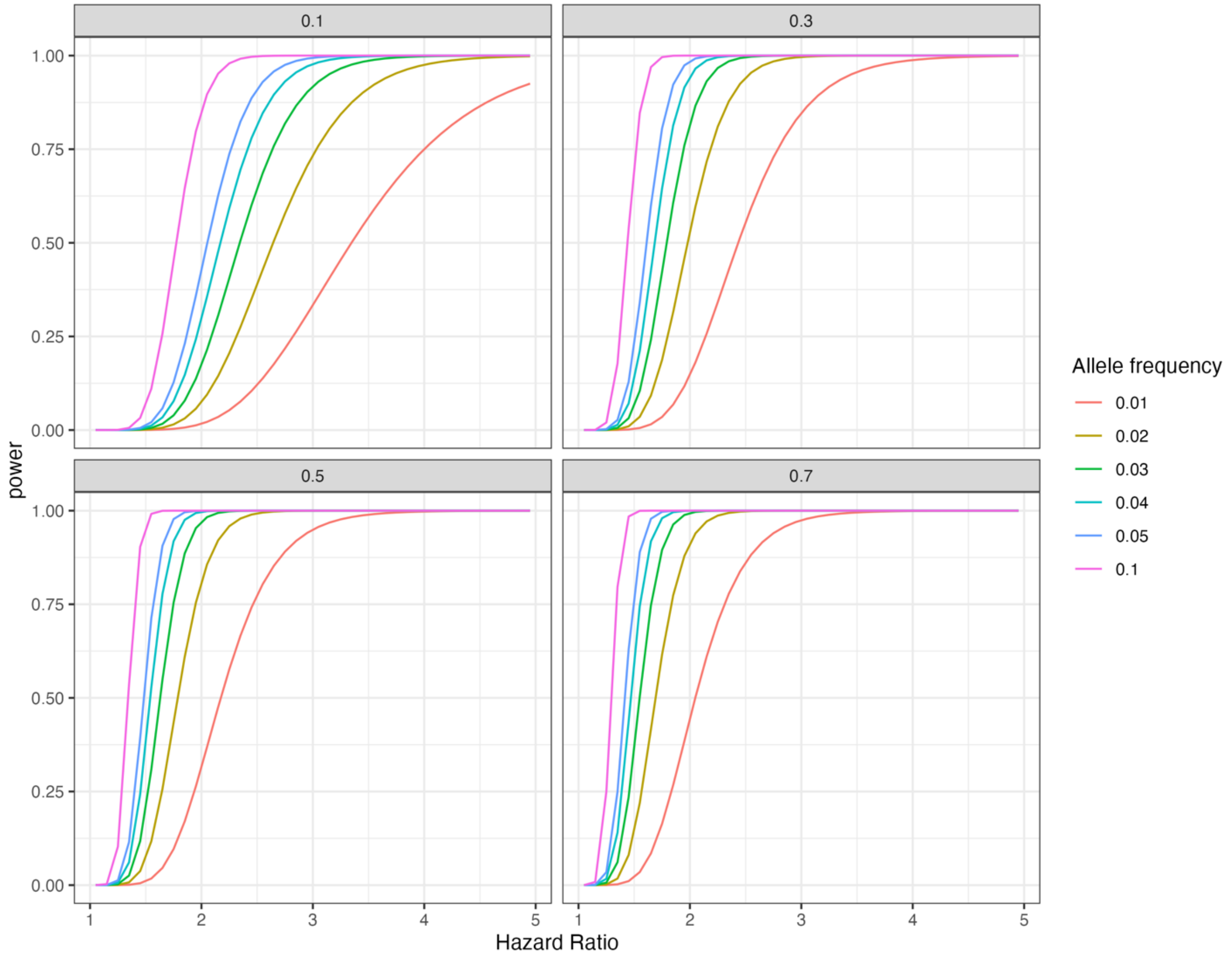

Supplementary Figure 14. Miami plot of mortality GWAS (top) and Hoehn and Yahr stage 3+ GWAS (bottom) in only cohorts that have both outcomes.

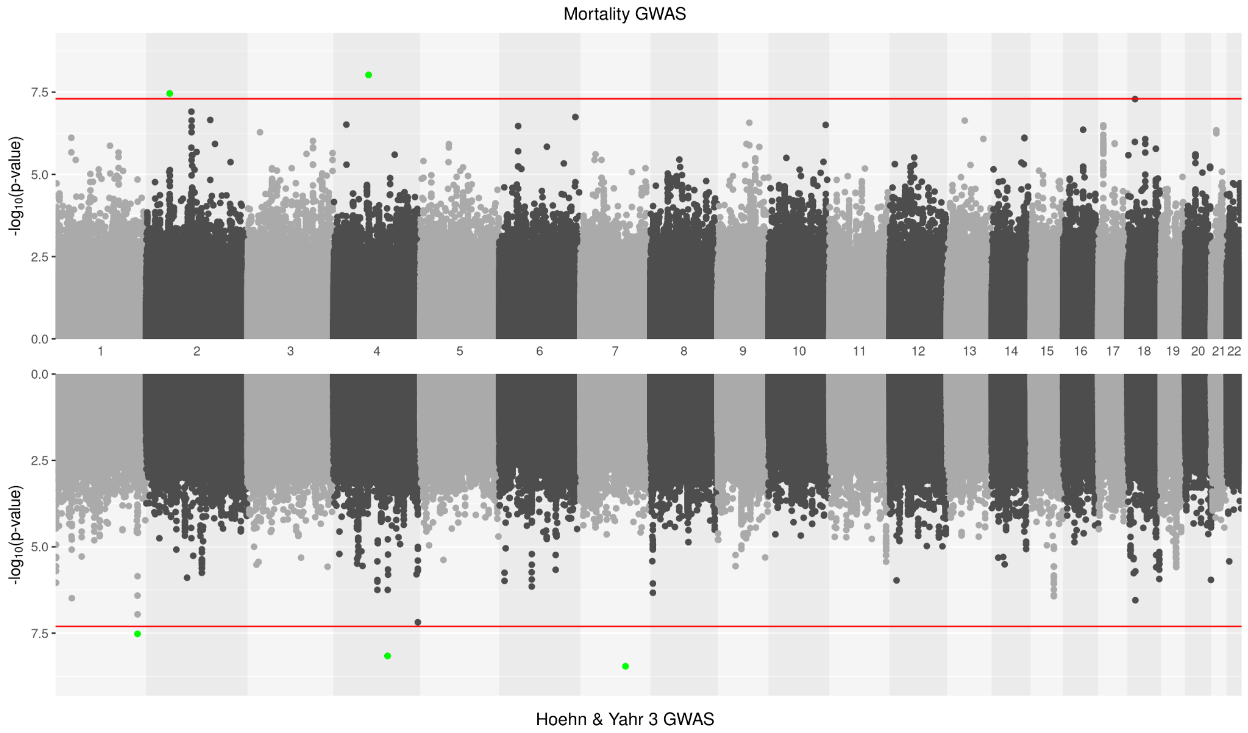

The GWASs were performed only in the four cohorts that had both outcomes available: Tracking Parkinson's, Oxford Discovery, DIGPD, and Oslo. The red line indicates the -log10 *p* value genome-wide significant threshold of $p = 5 \times 10^{-8}$. The green dots indicate variants passing genome-wide significance. Overall there does not appear to be shared signals between the mortality GWAS and Hoehn and Yahr stage 3+ GWAS, as the green highlighted signals are not shared/present in both GWASs.

GWAS = Genome Wide Association Study.

Supplementary Figure 15. Flowchart showing the fine-mapping pipeline in PAINTOR.

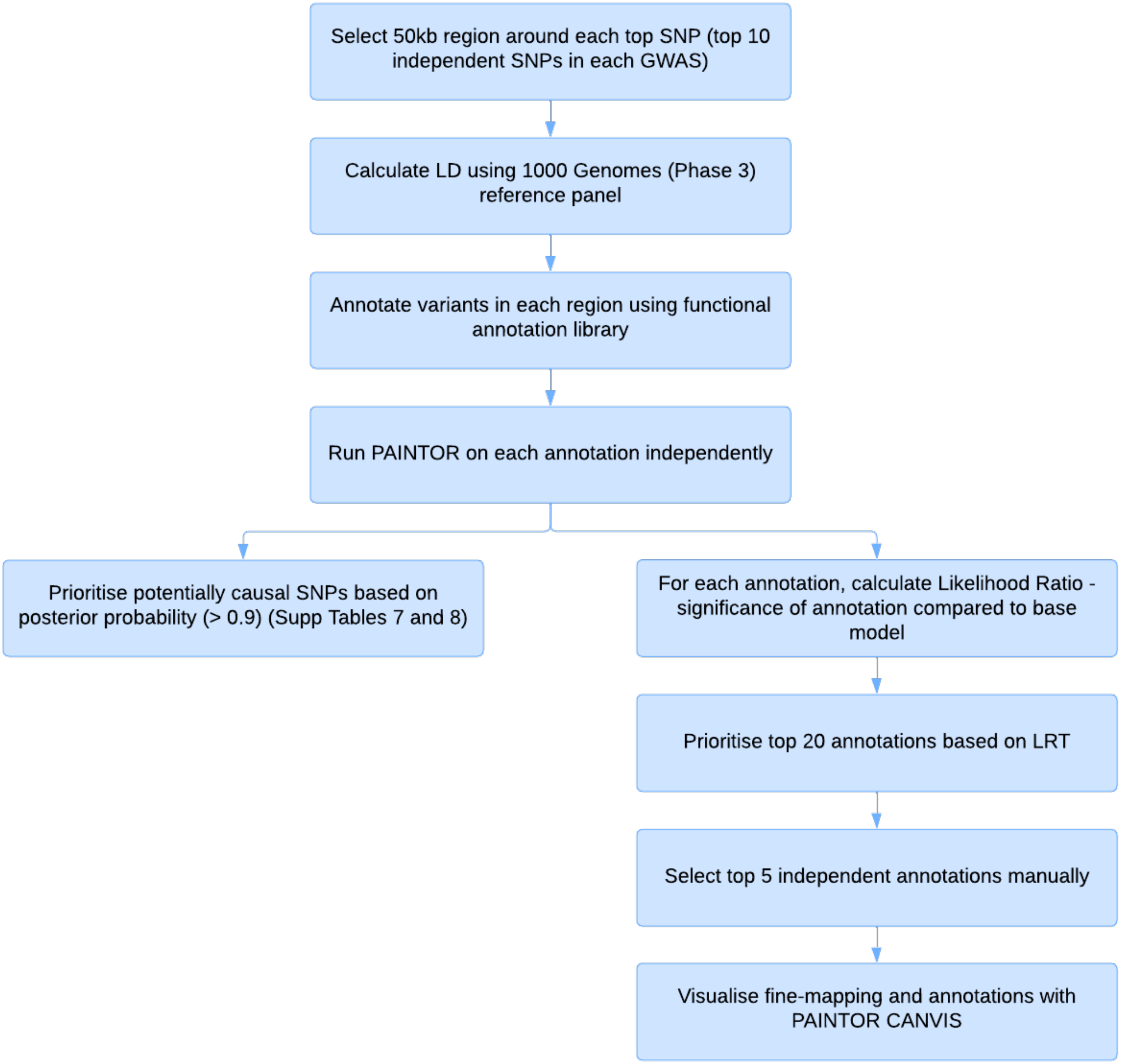

SUPPLEMENTARY TABLES

Supplementary Table 1. Study-specific lambdas (genomic inflation factor) for each GWAS

Cohort	Mortality	HY3
Tracking Parkinson's	1.02	1.01
Oxford Discovery	1.03	0.99
PPMI	NA	0.99
QSBB	1.15	NA
UKB - incident	1.03	NA
UKB - prevalent	0.97	NA
Calypso	1.10	NA
CamPaIGN	1.08	NA
Cambridge PD Research Clinic	1.27	NA
DIGPD	0.95	1.00
Trondheim	1.04	NA
Oslo	1.12	1.06
Meta-analysis (after filters)	1.04	1.03

Each GWAS was adjusted for age at onset, gender, and PC1-PC5 (see Methods). NAs indicate that cohort was not included for that GWAS, either because not enough individuals met the outcome or because relevant clinical data was not available (see Methods). Cohorts with genomic inflation factor of > 1.2 in a GWAS were excluded from meta-analysis of that outcome (highlighted in red).

CamPaiGN = Cambridgeshire Parkinson's Incidence from GP to Neurologist; DIGPD = Drug Interaction With Genes in Parkinson's Disease; HY3 = Hoehn and Yahr stage 3 or greater. PPMI = Parkinson's Progression Markers Initiative; QSBB = Queen Square Brain Bank pathologically-confirmed PD cases; UKB = UK Biobank

Supplementary Table 2. Full colocalisation results for PD mortality and eQTLs in eQTLGen and PsychENCODE.

prior_ type	GWAS_1	dataset	gene_2	hgnc_symbol	nsnps	PP.H0.abf	PP.H1.abf	PP.H2.abf	PP.H3.abf	PP.H4.abf	sum_PPH 3_PPH4	ratio_PH4_ PPH3	p12
liberal	PD_mortality	psychencode	ENSG00000104859	CLASRP	3296	5.28E-05	4.71E-01	1.20E-05	0.1062839	0.4226319	0.5289158	3.9764450	1.00E-05
robust	PD_mortality	psychencode	ENSG00000104859	CLASRP	3296	6.70E-05	5.97E-01	1.52E-05	0.1347610	0.2679349	0.4026959	1.9882225	5.00E-06
liberal	PD_mortality	psychencode	ENSG00000064393	HIPK2	2574	3.15E-03	6.89E-01	6.38E-04	0.1392676	0.1683492	0.3076169	1.2088182	1.00E-05
liberal	PD_mortality	psychencode	ENSG00000257435		4037	2.28E-03	5.92E-01	1.01E-03	0.2619352	0.1428419	0.4047771	0.5453331	1.00E-05
liberal	PD_mortality	psychencode	ENSG00000241661	PPP1R2P6	2433	2.88E-03	6.24E-01	1.28E-03	0.2776446	0.0940457	0.3716903	0.3387270	1.00E-05
robust	PD_mortality	psychencode	ENSG00000064393	HIPK2	2574	3.44E-03	7.52E-01	6.97E-04	0.1520679	0.0919112	0.2439791	0.6044091	5.00E-06
liberal	PD_mortality	psychencode	ENSG00000271537		2940	3.34E-03	7.44E-01	7.27E-04	0.1614372	0.0909891	0.2524263	0.5636193	1.00E-05
liberal	PD_mortality	psychencode	ENSG00000267114		3305	7.06E-05	6.30E-01	3.14E-05	0.2795213	0.0906603	0.3701816	0.3243414	1.00E-05
liberal	PD_mortality	psychencode	ENSG00000012061	ERCC1	3679	7.05E-05	6.29E-01	3.20E-05	0.2857667	0.0851910	0.3709578	0.2981138	1.00E-05
liberal	PD_mortality	psychencode	ENSG00000103200		2483	3.55E-03	7.69E-01	6.62E-04	0.1431077	0.0839033	0.2270110	0.5862944	1.00E-05
robust	PD_mortality	psychencode	ENSG00000257435		4037	2.46E-03	6.37E-01	1.09E-03	0.2820817	0.0769142	0.3589959	0.2726665	5.00E-06
liberal	PD_mortality	psychencode	ENSG00000270512		2422	3.18E-03	6.87E-01	1.08E-03	0.2332989	0.0750511	0.3083500	0.3216948	1.00E-05
liberal	PD_mortality	psychencode	ENSG00000087470	DNM1L	4155	2.79E-03	7.24E-01	7.81E-04	0.2025122	0.0694637	0.2719759	0.3430097	1.00E-05
liberal	PD_mortality	psychencode	ENSG00000240889	NDUFB2-AS1	2329	3.54E-03	7.62E-01	7.66E-04	0.1648099	0.0688845	0.2336944	0.4179633	1.00E-05
liberal	PD_mortality	psychencode	ENSG00000242675	RPS16P9	3743	7.97E-05	7.11E-01	2.47E-05	0.2200182	0.0687333	0.2887515	0.3123983	1.00E-05
liberal	PD_mortality	psychencode	ENSG00000253183		2800	3.54E-03	7.73E-01	7.09E-04	0.1545282	0.0679881	0.2225163	0.4399721	1.00E-05
liberal	PD_mortality	psychencode	ENSG00000059378	PARP12	2358	3.39E-03	7.40E-01	8.68E-04	0.1892351	0.0662562	0.2554913	0.3501263	1.00E-05
liberal	PD_mortality	psychencode	ENSG00000007047	MARK4	3336	8.58E-05	7.65E-01	1.92E-05	0.1715442	0.0635789	0.2351230	0.3706268	1.00E-05
liberal	PD_mortality	psychencode	ENSG00000229677		2970	3.42E-03	7.62E-01	7.69E-04	0.1715159	0.0620017	0.2335176	0.3614921	1.00E-05
liberal	PD_mortality	psychencode	ENSG00000240173	RN7SL771P	2357	3.57E-03	7.69E-01	7.66E-04	0.1650231	0.0613466	0.2263697	0.3717456	1.00E-05
liberal	PD_mortality	psychencode	ENSG00000271404		2864	3.50E-03	7.78E-01	7.16E-04	0.1592179	0.0586972	0.2179152	0.3686596	1.00E-05
liberal	PD_mortality	psychencode	ENSG00000146963	C7orf55-LUC7L2	2868	3.50E-03	7.72E-01	7.51E-04	0.1657247	0.0584782	0.2242029	0.3528635	1.00E-05
liberal	PD_mortality	psychencode	ENSG00000273391		2849	3.54E-03	7.81E-01	7.10E-04	0.1564343	0.0578420	0.2142763	0.3697527	1.00E-05
liberal	PD_mortality	psychencode	ENSG00000176182	MYPOP	3631	7.37E-05	6.52E-01	3.30E-05	0.2917257	0.0564488	0.3481746	0.1934996	1.00E-05
liberal	PD_mortality	psychencode	ENSG00000059377	TBXAS1	2577	3.52E-03	7.68E-01	7.86E-04	0.1717176	0.0556299	0.2273475	0.3239613	1.00E-05
liberal	PD_mortality	psychencode	ENSG00000236279	CLEC2L	2810	3.21E-03	7.02E-01	1.10E-03	0.2409026	0.0527959	0.2936984	0.2191586	1.00E-05
liberal	PD_mortality	psychencode	ENSG00000239254		2958	3.60E-03	8.01E-01	6.45E-04	0.1434352	0.0517783	0.1952135	0.3609874	1.00E-05
liberal	PD_mortality	psychencode	ENSG00000157741	UBN2	2866	3.57E-03	7.94E-01	6.78E-04	0.1506067	0.0514063	0.2020130	0.3413280	1.00E-05
liberal	PD_mortality	psychencode	ENSG00000159885	ZNF222	3417	8.25E-05	7.36E-01	2.39E-05	0.2128929	0.0506354	0.2635283	0.2378442	1.00E-05
liberal	PD_mortality	psychencode	ENSG00000228360		2864	3.52E-03	7.82E-01	7.33E-04	0.1629685	0.0505334	0.2135019	0.3100808	1.00E-05
liberal	PD_mortality	psychencode	ENSG00000146858	ZC3HAV1L	2965	3.52E-03	7.85E-01	7.21E-04	0.1607314	0.0503386	0.2110700	0.3131844	1.00E-05
liberal	PD_mortality	psychencode	ENSG00000146955	RAB19	2495	3.77E-03	8.17E-01	5.98E-04	0.1293450	0.0495833	0.1789284	0.3833418	1.00E-05

robust	PD_mortality	psychencode	ENSG00000241661	PPP1R2P6	2433	3.03E-03	6.55E-01	1.35E-03	0.2913444	0.0493431	0.3406876	0.1693635	5.00E-06
liberal	PD_mortality	psychencode	ENSG00000104881	PPP1R13L	3673	7.47E-05	6.66E-01	3.20E-05	0.2854679	0.0485583	0.3340262	0.1701007	1.00E-05
liberal	PD_mortality	psychencode	ENSG00000105948	TTC26	2969	3.32E-03	7.41E-01	9.29E-04	0.2069821	0.0482485	0.2552306	0.2331044	1.00E-05
robust	PD_mortality	psychencode	ENSG00000271537		2940	3.50E-03	7.79E-01	7.61E-04	0.1691318	0.0476630	0.2167948	0.2818096	5.00E-06
robust	PD_mortality	psychencode	ENSG00000267114		3305	7.40E-05	6.60E-01	3.28E-05	0.2927937	0.0474826	0.3402762	0.1621707	5.00E-06
liberal	PD_mortality	psychencode	ENSG00000254270	ERHP1	2823	3.50E-03	7.65E-01	8.37E-04	0.1828693	0.0473752	0.2302445	0.2590660	1.00E-05
liberal	PD_mortality	psychencode	ENSG00000157764	BRAF	2352	3.50E-03	7.52E-01	9.18E-04	0.1968230	0.0471145	0.2439375	0.2393750	1.00E-05
liberal	PD_mortality	psychencode	ENSG00000257316		4035	2.89E-03	7.49E-01	7.73E-04	0.2007236	0.0463033	0.2470269	0.2306817	1.00E-05
liberal	PD_mortality	psychencode	ENSG00000216588	IGSF23	3426	8.63E-05	7.70E-01	2.06E-05	0.1837740	0.0461781	0.2299521	0.2512765	1.00E-05
robust	PD_mortality	psychencode	ENSG00000012061	ERCC1	3679	7.36E-05	6.57E-01	3.35E-05	0.2984807	0.0444906	0.3429713	0.1490569	5.00E-06
robust	PD_mortality	psychencode	ENSG00000103200		2483	3.71E-03	8.02E-01	6.91E-04	0.1493742	0.0437886	0.1931629	0.2931472	5.00E-06
liberal	PD_mortality	psychencode	ENSG00000142273	CBLC	3387	7.60E-05	6.78E-01	3.13E-05	0.2791203	0.0428036	0.3219239	0.1533517	1.00E-05
liberal	PD_mortality	psychencode	ENSG00000159917	ZNF235	3527	6.79E-05	6.05E-01	3.95E-05	0.3525874	0.0420107	0.3945981	0.1191498	1.00E-05
liberal	PD_mortality	psychencode	ENSG00000241226	RN7SL836P	3769	7.33E-05	6.54E-01	3.43E-05	0.3057450	0.0405628	0.3463078	0.1326687	1.00E-05
liberal	PD_mortality	psychencode	ENSG00000256614		3028	3.10E-03	8.03E-01	5.93E-04	0.1539017	0.0390398	0.1929415	0.2536672	1.00E-05
robust	PD_mortality	psychencode	ENSG00000270512		2422	3.30E-03	7.14E-01	1.12E-03	0.2423949	0.0389886	0.2813835	0.1608474	5.00E-06
liberal	PD_mortality	psychencode	ENSG00000104941	RSPH6A	3746	8.13E-05	7.25E-01	2.65E-05	0.2362123	0.0389226	0.2751350	0.1647781	1.00E-05
liberal	PD_mortality	psychencode	ENSG00000267144		3417	8.79E-05	7.84E-01	2.00E-05	0.1781057	0.0378861	0.2159918	0.2127167	1.00E-05
liberal	PD_mortality	psychencode	ENSG00000267680	ZNF224	3452	8.24E-05	7.35E-01	2.54E-05	0.2267476	0.0377430	0.2644907	0.1664540	1.00E-05
liberal	PD_mortality	psychencode	ENSG00000270807		3948	2.99E-03	7.77E-01	7.01E-04	0.1820072	0.0370589	0.2190662	0.2036124	1.00E-05
robust	PD_mortality	psychencode	ENSG00000087470	DNM1L	4155	2.89E-03	7.51E-01	8.09E-04	0.2097989	0.0359815	0.2457805	0.1715049	5.00E-06
liberal	PD_mortality	psychencode	ENSG00000244743		4130	2.87E-03	7.46E-01	8.27E-04	0.2145739	0.0359021	0.2504760	0.1673179	1.00E-05
robust	PD_mortality	psychencode	ENSG00000240889	NDUFB2-AS1	2329	3.67E-03	7.89E-01	7.93E-04	0.1706888	0.0356708	0.2063596	0.2089817	5.00E-06
robust	PD_mortality	psychencode	ENSG00000242675	RPS16P9	3743	8.26E-05	7.36E-01	2.56E-05	0.2278486	0.0355898	0.2634383	0.1561991	5.00E-06
liberal	PD_mortality	psychencode	ENSG00000104856	RELB	3256	8.93E-05	7.96E-01	1.89E-05	0.1682908	0.0353860	0.2036769	0.2102670	1.00E-05
robust	PD_mortality	psychencode	ENSG00000253183		2800	3.67E-03	8.00E-01	7.34E-04	0.1599661	0.0351903	0.1951564	0.2199860	5.00E-06
liberal	PD_mortality	psychencode	ENSG00000185800	DMWD	3727	7.31E-05	6.52E-01	3.51E-05	0.3134229	0.0345039	0.3479268	0.1100875	1.00E-05
robust	PD_mortality	psychencode	ENSG00000059378	PARP12	2358	3.51E-03	7.66E-01	8.98E-04	0.1957189	0.0342632	0.2299821	0.1750632	5.00E-06
liberal	PD_mortality	psychencode	ENSG00000139131	YARS2	4163	2.93E-03	7.61E-01	7.82E-04	0.2028063	0.0329623	0.2357686	0.1625308	1.00E-05
robust	PD_mortality	psychencode	ENSG00000007047	MARK4	3336	8.86E-05	7.90E-01	1.99E-05	0.1771765	0.0328332	0.2100097	0.1853134	5.00E-06
robust	PD_mortality	psychencode	ENSG00000229677		2970	3.53E-03	7.87E-01	7.94E-04	0.1770031	0.0319926	0.2089958	0.1807461	5.00E-06

liberal	PD_mortality	psychencode	ENSG00000270679		3547	8.35E-05	7.45E-01	2.51E-05	0.2233927	0.0318996	0.2552923	0.1427959	1.00E-05
liberal	PD_mortality	psychencode	ENSG00000267348		3378	8.77E-05	7.82E-01	2.09E-05	0.1863274	0.0318523	0.2181797	0.1709481	1.00E-05
robust	PD_mortality	psychencode	ENSG00000240173	RN7SL771P	2357	3.68E-03	7.94E-01	7.90E-04	0.1702450	0.0316439	0.2018890	0.1858728	5.00E-06
liberal	PD_mortality	psychencode	ENSG00000267173		3480	8.92E-05	7.96E-01	1.94E-05	0.1725408	0.0316093	0.2041501	0.1831992	1.00E-05
liberal	PD_mortality	psychencode	ENSG00000125753	VASP	3729	8.17E-05	7.28E-01	2.69E-05	0.2400149	0.0315657	0.2715806	0.1315156	1.00E-05
liberal	PD_mortality	psychencode	ENSG00000007255	TRAPPC6A	3489	7.25E-05	6.47E-01	3.61E-05	0.3217212	0.0315455	0.3532667	0.0980523	1.00E-05
liberal	PD_mortality	psychencode	ENSG00000266921		3387	8.34E-05	7.44E-01	2.52E-05	0.2249156	0.0311191	0.2560347	0.1383592	1.00E-05
liberal	PD_mortality	psychencode	ENSG00000170608	FOXA3	3675	8.40E-05	7.49E-01	2.46E-05	0.2194315	0.0310167	0.2504482	0.1413504	1.00E-05
robust	PD_mortality	psychencode	ENSG00000271404		2864	3.60E-03	8.01E-01	7.38E-04	0.1640320	0.0302360	0.1942680	0.1843298	5.00E-06
liberal	PD_mortality	psychencode	ENSG00000187244	BCAM	3339	8.66E-05	7.72E-01	2.22E-05	0.1976900	0.0302038	0.2278938	0.1527837	1.00E-05
robust	PD_mortality	psychencode	ENSG00000146963	C7orf55-LUC7L2	2868	3.60E-03	7.95E-01	7.74E-04	0.1707163	0.0301198	0.2008360	0.1764318	5.00E-06
robust	PD_mortality	psychencode	ENSG00000273391		2849	3.65E-03	8.05E-01	7.31E-04	0.1610932	0.0297823	0.1908756	0.1848763	5.00E-06
liberal	PD_mortality	psychencode	ENSG00000255652		3056	2.79E-03	7.23E-01	9.38E-04	0.2434276	0.0295494	0.2729770	0.1213890	1.00E-05
robust	PD_mortality	psychencode	ENSG00000176182	MYPOP	3631	7.58E-05	6.71E-01	3.40E-05	0.3001987	0.0290442	0.3292428	0.0967498	5.00E-06
liberal	PD_mortality	psychencode	ENSG00000267346	EIF5AP3	3455	8.62E-05	7.68E-01	2.27E-05	0.2024186	0.0290281	0.2314466	0.1434063	1.00E-05
liberal	PD_mortality	psychencode	ENSG00000130203	APOE	3323	9.01E-05	8.03E-01	1.88E-05	0.1675427	0.0288744	0.1964171	0.1723408	1.00E-05
liberal	PD_mortality	psychencode	ENSG00000267545		3494	8.74E-05	7.79E-01	2.15E-05	0.1918428	0.0287814	0.2206242	0.1500259	1.00E-05
robust	PD_mortality	psychencode	ENSG00000059377	TBXAS1	2577	3.62E-03	7.90E-01	8.09E-04	0.1766306	0.0286107	0.2052413	0.1619807	5.00E-06
liberal	PD_mortality	psychencode	ENSG00000267188		3408	8.44E-05	7.53E-01	2.45E-05	0.2184139	0.0285881	0.2470021	0.1308896	1.00E-05
liberal	PD_mortality	psychencode	ENSG00000266958		3500	8.38E-05	7.47E-01	2.51E-05	0.2239439	0.0284674	0.2524113	0.1271184	1.00E-05
liberal	PD_mortality	psychencode	ENSG00000230666	CEACAM22P	3417	8.35E-05	7.45E-01	2.54E-05	0.2266284	0.0283393	0.2549677	0.1250476	1.00E-05
liberal	PD_mortality	psychencode	ENSG00000267590	NDUFA3P1	3539	8.60E-05	7.67E-01	2.30E-05	0.2051865	0.0281646	0.2333511	0.1372635	1.00E-05
liberal	PD_mortality	psychencode	ENSG00000167380	ZNF226	3446	2.02E-05	1.80E-01	8.87E-05	0.7914004	0.0280923	0.8194926	0.0354969	1.00E-05
liberal	PD_mortality	psychencode	ENSG00000131115	ZNF227	3482	8.69E-05	7.75E-01	2.21E-05	0.1967812	0.0280862	0.2248674	0.1427280	1.00E-05
liberal	PD_mortality	psychencode	ENSG00000269148		3744	8.21E-05	7.32E-01	2.69E-05	0.2395878	0.0280027	0.2675905	0.1168788	1.00E-05
liberal	PD_mortality	psychencode	ENSG00000214855	APOC1P1	3362	4.47E-05	3.98E-01	6.44E-05	0.5742495	0.0273266	0.6015760	0.0475866	1.00E-05
liberal	PD_mortality	psychencode	ENSG00000267757	C19orf83	3763	8.47E-05	7.56E-01	2.43E-05	0.2167322	0.0272997	0.2440318	0.1259604	1.00E-05
liberal	PD_mortality	psychencode	ENSG00000267037		3457	8.59E-05	7.66E-01	2.31E-05	0.2064192	0.0272042	0.2336234	0.1317909	1.00E-05
liberal	PD_mortality	psychencode	ENSG00000256294	ZNF225	3450	8.43E-05	7.52E-01	2.47E-05	0.2206282	0.0271976	0.2478259	0.1232735	1.00E-05
liberal	PD_mortality	psychencode	ENSG00000265842	RN7SL53P	3443	8.48E-05	7.56E-01	2.43E-05	0.2167559	0.0271579	0.2439138	0.1252926	1.00E-05
liberal	PD_mortality	psychencode	ENSG00000259605		3689	8.54E-05	7.61E-01	2.37E-05	0.2113307	0.0271209	0.2384516	0.1283338	1.00E-05
robust	PD_mortality	psychencode	ENSG00000236279	CLEC2L	2810	3.30E-03	7.21E-01	1.13E-03	0.2474343	0.0271137	0.2745480	0.1095793	5.00E-06
liberal	PD_mortality	psychencode	ENSG00000267282		3311	8.38E-05	7.47E-01	2.53E-05	0.2260092	0.0268181	0.2528273	0.1186591	1.00E-05
liberal	PD_mortality	psychencode	ENSG00000177045	SIX5	3693	8.41E-05	7.50E-01	2.50E-05	0.2227560	0.0268068	0.2495628	0.1203413	1.00E-05

liberal	PD_mortality	psychencode	ENSG00000267045		3577	8.26E-05	7.37E-01	2.65E-05	0.2366102	0.0266157	0.2632258	0.1124874	1.00E-05
robust	PD_mortality	psychencode	ENSG00000239254		2958	3.70E-03	8.22E-01	6.62E-04	0.1472473	0.0265772	0.1738246	0.1804937	5.00E-06
robust	PD_mortality	psychencode	ENSG00000157741	UBN2	2866	3.67E-03	8.15E-01	6.96E-04	0.1545799	0.0263812	0.1809611	0.1706640	5.00E-06
liberal	PD_mortality	psychencode	ENSG00000268434		3719	8.43E-05	7.52E-01	2.49E-05	0.2217920	0.0262009	0.2479929	0.1181329	1.00E-05
liberal	PD_mortality	psychencode	ENSG00000125740	FOSB	3720	8.65E-05	7.71E-01	2.27E-05	0.2026241	0.0261021	0.2287262	0.1288205	1.00E-05
robust	PD_mortality	psychencode	ENSG00000159885	ZNF222	3417	8.47E-05	7.55E-01	2.45E-05	0.2184229	0.0259753	0.2443982	0.1189221	5.00E-06
liberal	PD_mortality	psychencode	ENSG00000267044		3462	8.72E-05	7.78E-01	2.20E-05	0.1959133	0.0259662	0.2218795	0.1325390	1.00E-05
liberal	PD_mortality	psychencode	ENSG00000245482		3388	2.39E-03	6.21E-01	1.34E-03	0.3491484	0.0259358	0.3750841	0.0742830	1.00E-05
robust	PD_mortality	psychencode	ENSG00000228360		2864	3.61E-03	8.03E-01	7.52E-04	0.1671929	0.0259217	0.1931145	0.1550404	5.00E-06
liberal	PD_mortality	eQTLGen	ENSG00000167384	ZNF180	5445	3.19E-05	4.21E-01	4.20E-05	0.5532884	0.0258634	0.5791518	0.0467449	1.00E-05
liberal	PD_mortality	psychencode	ENSG00000177464	GPR4	3763	8.56E-05	7.63E-01	2.36E-05	0.2108005	0.0258313	0.2366318	0.1225389	1.00E-05
robust	PD_mortality	psychencode	ENSG00000146858	ZC3HAV1L	2965	3.61E-03	8.05E-01	7.39E-04	0.1648814	0.0258191	0.1907005	0.1565922	5.00E-06
liberal	PD_mortality	psychencode	ENSG00000069399	BCL3	3410	7.89E-05	7.04E-01	3.04E-05	0.2706562	0.0256639	0.2963201	0.0948211	1.00E-05
liberal	PD_mortality	psychencode	ENSG00000130201	EXOC3L2	3543	8.54E-05	7.61E-01	2.39E-05	0.2130907	0.0255151	0.2386058	0.1197382	1.00E-05
liberal	PD_mortality	psychencode	ENSG00000267163		3453	7.94E-05	7.08E-01	2.99E-05	0.2664028	0.0255088	0.2919116	0.0957527	1.00E-05
liberal	PD_mortality	psychencode	ENSG00000104884	ERCC2	3699	8.55E-05	7.62E-01	2.38E-05	0.2121148	0.0254622	0.2375770	0.1200398	1.00E-05
robust	PD_mortality	psychencode	ENSG00000146955	RAB19	2495	3.87E-03	8.37E-01	6.13E-04	0.1326332	0.0254219	0.1580552	0.1916709	5.00E-06
liberal	PD_mortality	psychencode	ENSG00000057294	PKP2	4046	2.43E-03	6.30E-01	1.31E-03	0.3410271	0.0253124	0.3663395	0.0742241	1.00E-05
liberal	PD_mortality	psychencode	ENSG00000130204	TOMM40	3313	7.81E-05	6.96E-01	3.12E-05	0.2782434	0.0251898	0.3034332	0.0905313	1.00E-05
liberal	PD_mortality	psychencode	ENSG00000267395		3689	8.57E-05	7.64E-01	2.36E-05	0.2107726	0.0251353	0.2359079	0.1192533	1.00E-05
liberal	PD_mortality	psychencode	ENSG00000104879	CKM	3741	7.62E-05	6.80E-01	3.31E-05	0.2951245	0.0251204	0.3202449	0.0851180	1.00E-05
liberal	PD_mortality	psychencode	ENSG00000177051	FBXO46	3709	6.95E-05	6.20E-01	3.98E-05	0.3548043	0.0250874	0.3798917	0.0707077	1.00E-05
robust	PD_mortality	psychencode	ENSG00000104881	PPP1R13L	3673	7.65E-05	6.82E-01	3.28E-05	0.2925713	0.0248833	0.3174546	0.0850504	5.00E-06
liberal	PD_mortality	psychencode	ENSG00000267467	APOC4	3336	8.23E-05	7.34E-01	2.70E-05	0.2409704	0.0248190	0.2657894	0.1029962	1.00E-05
robust	PD_mortality	psychencode	ENSG00000105948	TTC26	2969	3.41E-03	7.59E-01	9.52E-04	0.2120989	0.0247206	0.2368194	0.1165522	5.00E-06
liberal	PD_mortality	psychencode	ENSG00000104853	CLPTM1	3304	7.26E-05	6.47E-01	3.68E-05	0.3280802	0.0247179	0.3527981	0.0753411	1.00E-05
liberal	PD_mortality	psychencode	ENSG00000104866	PPP1R37	3385	7.69E-05	6.85E-01	3.25E-05	0.2897808	0.0246572	0.3144380	0.0850893	1.00E-05
robust	PD_mortality	psychencode	ENSG00000254270	ERHP1	2823	3.59E-03	7.84E-01	8.58E-04	0.1873061	0.0242623	0.2115684	0.1295330	5.00E-06
robust	PD_mortality	psychencode	ENSG00000157764	BRAF	2352	3.59E-03	7.70E-01	9.40E-04	0.2015715	0.0241256	0.2256970	0.1196875	5.00E-06
robust	PD_mortality	psychencode	ENSG00000257316		4035	2.95E-03	7.67E-01	7.92E-04	0.2054809	0.0237003	0.2291812	0.1153409	5.00E-06
robust	PD_mortality	psychencode	ENSG00000216588	IGSF23	3426	8.84E-05	7.88E-01	2.11E-05	0.1881174	0.0236347	0.2117522	0.1256382	5.00E-06
liberal	PD_mortality	psychencode	ENSG00000268341		3734	5.00E-05	4.46E-01	5.95E-05	0.5308652	0.0228820	0.5537473	0.0431033	1.00E-05
robust	PD_mortality	psychencode	ENSG00000142273	CBLC	3387	7.77E-05	6.93E-01	3.20E-05	0.2852247	0.0218698	0.3070945	0.0766759	5.00E-06
liberal	PD_mortality	psychencode	ENSG00000263002	ZNF234	3437	6.92E-05	6.18E-01	4.05E-05	0.3608785	0.0215076	0.3823861	0.0595979	1.00E-05

robust	PD_mortality	psychencode	ENSG00000159917	ZNF235	3527	6.93E-05	6.18E-01	4.04E-05	0.3601526	0.0214560	0.3816086	0.0595749	5.00E-06
liberal	PD_mortality	psychencode	ENSG00000170684	ZNF296	3325	6.09E-05	5.43E-01	4.89E-05	0.4358993	0.0207903	0.4566896	0.0476952	1.00E-05
liberal	PD_mortality	psychencode	ENSG00000257511		4156	1.94E-03	5.04E-01	1.82E-03	0.4710372	0.0207382	0.4917755	0.0440267	1.00E-05
robust	PD_mortality	psychencode	ENSG00000241226	RN7SL836P	3769	7.48E-05	6.67E-01	3.50E-05	0.3120743	0.0207012	0.3327755	0.0663343	5.00E-06
liberal	PD_mortality	psychencode	ENSG00000267242		3419	6.88E-05	6.13E-01	4.11E-05	0.3663193	0.0202690	0.3865882	0.0553314	1.00E-05
robust	PD_mortality	psychencode	ENSG00000256614		3028	3.16E-03	8.19E-01	6.05E-04	0.1569656	0.0199085	0.1768741	0.1268336	5.00E-06
robust	PD_mortality	psychencode	ENSG00000104941	RSPH6A	3746	8.29E-05	7.39E-01	2.70E-05	0.2409006	0.0198476	0.2607482	0.0823890	5.00E-06
liberal	PD_mortality	eQTLGen	ENSG00000159905	ZNF221	5645	2.93E-05	3.87E-01	4.50E-05	0.5937755	0.0195798	0.6133553	0.0329751	1.00E-05
robust	PD_mortality	psychencode	ENSG00000267144		3417	8.96E-05	7.99E-01	2.04E-05	0.1815447	0.0193088	0.2008535	0.1063583	5.00E-06
robust	PD_mortality	psychencode	ENSG00000267680	ZNF224	3452	8.40E-05	7.50E-01	2.59E-05	0.2311090	0.0192345	0.2503435	0.0832270	5.00E-06
liberal	PD_mortality	eQTLGen	ENSG00000207003	RNU6-611P	5140	5.89E-05	7.76E-01	1.55E-05	0.2046577	0.0190825	0.2237401	0.0932408	1.00E-05
robust	PD_mortality	psychencode	ENSG00000270807		3948	3.05E-03	7.92E-01	7.14E-04	0.1854434	0.0188793	0.2043227	0.1018062	5.00E-06
liberal	PD_mortality	psychencode	ENSG00000178386	ZNF223	3437	6.07E-05	5.42E-01	4.92E-05	0.4392666	0.0188236	0.4580902	0.0428523	1.00E-05
robust	PD_mortality	psychencode	ENSG00000244743		4130	2.93E-03	7.59E-01	8.42E-04	0.2184961	0.0182792	0.2367753	0.0836590	5.00E-06
liberal	PD_mortality	psychencode	ENSG00000186019		3447	4.78E-05	4.26E-01	6.23E-05	0.5555221	0.0181420	0.5736641	0.0326575	1.00E-05
liberal	PD_mortality	psychencode	ENSG00000117877	CD3EAP	3672	4.47E-06	3.99E-02	1.06E-04	0.9419639	0.0180628	0.9600268	0.0191757	1.00E-05
robust	PD_mortality	psychencode	ENSG00000104856	RELB	3256	9.09E-05	8.11E-01	1.92E-05	0.1713221	0.0180117	0.1893337	0.1051335	5.00E-06
liberal	PD_mortality	psychencode	ENSG00000179846	NKPD1	3462	2.43E-05	2.17E-01	8.58E-05	0.7648966	0.0179050	0.7828016	0.0234084	1.00E-05
robust	PD_mortality	psychencode	ENSG00000185800	DMWD	3727	7.44E-05	6.63E-01	3.58E-05	0.3189250	0.0175548	0.3364798	0.0550437	5.00E-06
liberal	PD_mortality	eQTLGen	ENSG00000267163		5420	5.57E-05	7.34E-01	1.88E-05	0.2482489	0.0174764	0.2657253	0.0703988	1.00E-05
liberal	PD_mortality	psychencode	ENSG00000130208	APOC1	3337	2.15E-05	1.92E-01	8.87E-05	0.7909867	0.0172044	0.8081911	0.0217505	1.00E-05
liberal	PD_mortality	eQTLGen	ENSG00000267114		5172	5.30E-05	6.98E-01	2.16E-05	0.2847763	0.0168391	0.3016154	0.0591311	1.00E-05
robust	PD_mortality	psychencode	ENSG00000139131	YARS2	4163	2.98E-03	7.73E-01	7.95E-04	0.2062048	0.0167573	0.2229621	0.0812654	5.00E-06
liberal	PD_mortality	eQTLGen	ENSG00000240889	NDUFB2-AS1	4206	8.12E-04	6.58E-01	4.00E-04	0.3237813	0.0167239	0.3405052	0.0516518	1.00E-05
robust	PD_mortality	psychencode	ENSG00000270679		3547	8.48E-05	7.57E-01	2.55E-05	0.2270135	0.0162083	0.2432218	0.0713979	5.00E-06
robust	PD_mortality	psychencode	ENSG00000267348		3378	8.91E-05	7.94E-01	2.12E-05	0.1893429	0.0161839	0.2055268	0.0854740	5.00E-06
robust	PD_mortality	psychencode	ENSG00000267173		3480	9.07E-05	8.09E-01	1.97E-05	0.1753115	0.0160585	0.1913700	0.0915996	5.00E-06
robust	PD_mortality	psychencode	ENSG00000125753	VASP	3729	8.30E-05	7.40E-01	2.73E-05	0.2438637	0.0160359	0.2598997	0.0657578	5.00E-06
robust	PD_mortality	psychencode	ENSG00000007255	TRAPPC6A	3489	7.37E-05	6.57E-01	3.67E-05	0.3268770	0.0160255	0.3429025	0.0490262	5.00E-06
robust	PD_mortality	psychencode	ENSG00000266921		3387	8.47E-05	7.56E-01	2.56E-05	0.2284705	0.0158055	0.2442760	0.0691796	5.00E-06
robust	PD_mortality	psychencode	ENSG00000170608	FOXA3	3675	8.53E-05	7.61E-01	2.50E-05	0.2228881	0.0157527	0.2386407	0.0706752	5.00E-06
robust	PD_mortality	psychencode	ENSG00000187244	BCAM	3339	8.79E-05	7.84E-01	2.25E-05	0.2007213	0.0153335	0.2160548	0.0763918	5.00E-06
robust	PD_mortality	psychencode	ENSG00000255652		3056	2.83E-03	7.34E-01	9.52E-04	0.2470781	0.0149963	0.2620743	0.0606945	5.00E-06
robust	PD_mortality	psychencode	ENSG00000267346	EIF5AP3	3455	8.74E-05	7.80E-01	2.30E-05	0.2053997	0.0147278	0.2201275	0.0717031	5.00E-06

robust	PD_mortality	psychencode	ENSG00000130203	APOE	3323	9.14E-05	8.15E-01	1.91E-05	0.1699970	0.0146487	0.1846457	0.0861704	5.00E-06
robust	PD_mortality	psychencode	ENSG00000267545		3494	8.87E-05	7.91E-01	2.18E-05	0.1946439	0.0146008	0.2092447	0.0750130	5.00E-06
robust	PD_mortality	psychencode	ENSG00000267188		3408	8.57E-05	7.64E-01	2.49E-05	0.2215812	0.0145013	0.2360826	0.0654448	5.00E-06
liberal	PD_mortality	psychencode	ENSG00000257643		4147	1.40E-03	3.63E-01	2.38E-03	0.6183969	0.0144604	0.6328573	0.0233836	1.00E-05
robust	PD_mortality	psychencode	ENSG00000266958		3500	8.50E-05	7.58E-01	2.55E-05	0.2271775	0.0144392	0.2416167	0.0635592	5.00E-06
robust	PD_mortality	psychencode	ENSG00000230666	CEACAM22P	3417	8.47E-05	7.56E-01	2.58E-05	0.2298858	0.0143733	0.2442591	0.0625238	5.00E-06
robust	PD_mortality	psychencode	ENSG00000267590	NDUFA3P1	3539	8.72E-05	7.77E-01	2.33E-05	0.2081172	0.0142834	0.2224007	0.0686317	5.00E-06
robust	PD_mortality	psychencode	ENSG00000167380	ZNF226	3446	2.05E-05	1.83E-01	9.00E-05	0.8026748	0.0142462	0.8169211	0.0177485	5.00E-06
robust	PD_mortality	psychencode	ENSG00000131115	ZNF227	3482	8.81E-05	7.86E-01	2.24E-05	0.1995840	0.0142431	0.2138271	0.0713640	5.00E-06
robust	PD_mortality	psychencode	ENSG00000269148		3744	8.33E-05	7.43E-01	2.72E-05	0.2429900	0.0142002	0.2571901	0.0584394	5.00E-06
robust	PD_mortality	psychencode	ENSG00000214855	APOC1P1	3362	4.53E-05	4.04E-01	6.53E-05	0.5822043	0.0138526	0.5960568	0.0237933	5.00E-06
robust	PD_mortality	psychencode	ENSG00000267757	C19orf83	3763	8.59E-05	7.66E-01	2.46E-05	0.2197315	0.0138387	0.2335702	0.0629802	5.00E-06
robust	PD_mortality	psychencode	ENSG00000267037		3457	8.71E-05	7.77E-01	2.35E-05	0.2092656	0.0137897	0.2230553	0.0658955	5.00E-06
robust	PD_mortality	psychencode	ENSG00000256294	ZNF225	3450	8.55E-05	7.62E-01	2.51E-05	0.2236699	0.0137863	0.2374562	0.0616367	5.00E-06
robust	PD_mortality	psychencode	ENSG00000265842	RN7SL53P	3443	8.59E-05	7.66E-01	2.46E-05	0.2197397	0.0137659	0.2335056	0.0626463	5.00E-06
robust	PD_mortality	psychencode	ENSG00000259605		3689	8.65E-05	7.72E-01	2.40E-05	0.2142358	0.0137468	0.2279827	0.0641669	5.00E-06
robust	PD_mortality	psychencode	ENSG00000267282		3311	8.49E-05	7.57E-01	2.57E-05	0.2290810	0.0135913	0.2426722	0.0593296	5.00E-06
robust	PD_mortality	psychencode	ENSG00000177045	SIX5	3693	8.53E-05	7.61E-01	2.53E-05	0.2257823	0.0135855	0.2393678	0.0601707	5.00E-06
robust	PD_mortality	psychencode	ENSG00000267045		3577	8.37E-05	7.47E-01	2.69E-05	0.2398014	0.0134873	0.2532887	0.0562437	5.00E-06
liberal	PD_mortality	eQTLGen	ENSG00000269148		5467	5.06E-05	6.66E-01	2.43E-05	0.3200690	0.0134804	0.3335494	0.0421172	1.00E-05
robust	PD_mortality	psychencode	ENSG00000268434		3719	8.54E-05	7.62E-01	2.52E-05	0.2247362	0.0132744	0.2380105	0.0590664	5.00E-06
robust	PD_mortality	psychencode	ENSG00000125740	FOSB	3720	8.76E-05	7.81E-01	2.30E-05	0.2053035	0.0132236	0.2185271	0.0644103	5.00E-06
liberal	PD_mortality	psychencode	ENSG00000006459	KDM7A	2342	5.34E-04	1.16E-01	3.98E-03	0.8660023	0.0131675	0.8791698	0.0152049	1.00E-05
robust	PD_mortality	psychencode	ENSG00000267044		3462	8.84E-05	7.88E-01	2.23E-05	0.1984903	0.0131539	0.2116442	0.0662695	5.00E-06
robust	PD_mortality	psychencode	ENSG00000245482		3388	2.42E-03	6.29E-01	1.36E-03	0.3537356	0.0131383	0.3668738	0.0371415	5.00E-06
robust	PD_mortality	eQTLGen	ENSG00000167384	ZNF180	5445	3.24E-05	4.26E-01	4.26E-05	0.5605371	0.0131011	0.5736382	0.0233724	5.00E-06
robust	PD_mortality	psychencode	ENSG00000177464	GPR4	3763	8.67E-05	7.73E-01	2.39E-05	0.2135588	0.0130846	0.2266434	0.0612694	5.00E-06
robust	PD_mortality	psychencode	ENSG00000069399	BCL3	3410	7.99E-05	7.13E-01	3.07E-05	0.2741744	0.0129988	0.2871731	0.0474106	5.00E-06
robust	PD_mortality	psychencode	ENSG00000130201	EXOC3L2	3543	8.65E-05	7.71E-01	2.42E-05	0.2158444	0.0129224	0.2287668	0.0598691	5.00E-06
robust	PD_mortality	psychencode	ENSG00000267163		3453	8.04E-05	7.17E-01	3.03E-05	0.2698445	0.0129192	0.2827636	0.0478763	5.00E-06
robust	PD_mortality	psychencode	ENSG00000104884	ERCC2	3699	8.66E-05	7.72E-01	2.41E-05	0.2148501	0.0128953	0.2277453	0.0600199	5.00E-06
robust	PD_mortality	psychencode	ENSG00000057294	PKP2	4046	2.46E-03	6.38E-01	1.33E-03	0.3453985	0.0128185	0.3582170	0.0371121	5.00E-06
robust	PD_mortality	psychencode	ENSG00000130204	TOMM40	3313	7.91E-05	7.05E-01	3.16E-05	0.2817926	0.0127555	0.2945481	0.0452657	5.00E-06
robust	PD_mortality	psychencode	ENSG00000267395		3689	8.67E-05	7.74E-01	2.39E-05	0.2134552	0.0127276	0.2261828	0.0596267	5.00E-06

robust	PD_mortality	psychencode	ENSG00000104879	CKM	3741	7.72E-05	6.88E-01	3.35E-05	0.2988785	0.0127200	0.3115985	0.0425590	5.00E-06
robust	PD_mortality	psychencode	ENSG00000177051	FBXO46	3709	7.04E-05	6.28E-01	4.03E-05	0.3593114	0.0127030	0.3720144	0.0353539	5.00E-06
robust	PD_mortality	psychencode	ENSG00000267467	APOC4	3336	8.33E-05	7.43E-01	2.74E-05	0.2439983	0.0125654	0.2565637	0.0514981	5.00E-06
robust	PD_mortality	psychencode	ENSG00000104853	CLPTM1	3304	7.35E-05	6.55E-01	3.72E-05	0.3321856	0.0125136	0.3446993	0.0376706	5.00E-06
robust	PD_mortality	psychencode	ENSG00000104866	PPP1R37	3385	7.78E-05	6.94E-01	3.29E-05	0.2933980	0.0124825	0.3058805	0.0425446	5.00E-06
robust	PD_mortality	psychencode	ENSG00000268341		3734	5.06E-05	4.51E-01	6.02E-05	0.5370092	0.0115734	0.5485826	0.0215516	5.00E-06
liberal	PD_mortality	psychencode	ENSG00000122778	KIAA1549	2945	7.79E-04	1.74E-01	3.63E-03	0.8106804	0.0110927	0.8217731	0.0136832	1.00E-05
robust	PD_mortality	psychencode	ENSG00000263002	ZNF234	3437	7.00E-05	6.24E-01	4.09E-05	0.3648015	0.0108707	0.3756722	0.0297990	5.00E-06
robust	PD_mortality	psychencode	ENSG00000170684	ZNF296	3325	6.15E-05	5.49E-01	4.94E-05	0.4404781	0.0105043	0.4509825	0.0238476	5.00E-06
robust	PD_mortality	psychencode	ENSG00000257511		4156	1.96E-03	5.10E-01	1.83E-03	0.4759727	0.0104778	0.4864504	0.0220134	5.00E-06
robust	PD_mortality	psychencode	ENSG00000267242		3419	6.95E-05	6.20E-01	4.15E-05	0.3700697	0.0102382	0.3803080	0.0276657	5.00E-06
robust	PD_mortality	eQTLGen	ENSG00000159905	ZNF221	5645	2.96E-05	3.90E-01	4.55E-05	0.5996460	0.0098867	0.6095327	0.0164875	5.00E-06
robust	PD_mortality	eQTLGen	ENSG00000207003	RNU6-611P	5140	5.95E-05	7.84E-01	1.57E-05	0.2066292	0.0096331	0.2162623	0.0466204	5.00E-06
robust	PD_mortality	psychencode	ENSG00000178386	ZNF223	3437	6.13E-05	5.47E-01	4.97E-05	0.4434402	0.0095012	0.4529414	0.0214261	5.00E-06
robust	PD_mortality	psychencode	ENSG00000186019		3447	4.82E-05	4.30E-01	6.29E-05	0.5606074	0.0091540	0.5697614	0.0163288	5.00E-06
robust	PD_mortality	psychencode	ENSG00000117877	CD3EAP	3672	4.51E-06	4.02E-02	1.07E-04	0.9505487	0.0091137	0.9596624	0.0095879	5.00E-06
robust	PD_mortality	psychencode	ENSG00000179846	NKPD1	3462	2.46E-05	2.19E-01	8.65E-05	0.7718062	0.0090334	0.7808396	0.0117042	5.00E-06
liberal	PD_mortality	eQTLGen	ENSG00000130204	TOMM40	5199	5.04E-06	6.65E-02	7.02E-05	0.9245588	0.0089147	0.9334735	0.0096421	1.00E-05
robust	PD_mortality	eQTLGen	ENSG00000267163		5420	5.62E-05	7.41E-01	1.90E-05	0.2504372	0.0088152	0.2592525	0.0351994	5.00E-06
robust	PD_mortality	psychencode	ENSG00000130208	APOC1	3337	2.17E-05	1.93E-01	8.95E-05	0.7978499	0.0086768	0.8065268	0.0108753	5.00E-06
liberal	PD_mortality	eQTLGen	ENSG00000130203	APOE	5224	6.72E-05	8.86E-01	8.00E-06	0.1053352	0.0085487	0.1138838	0.0811568	1.00E-05
robust	PD_mortality	eQTLGen	ENSG00000267114		5172	5.34E-05	7.04E-01	2.18E-05	0.2871943	0.0084911	0.2956854	0.0295655	5.00E-06
robust	PD_mortality	eQTLGen	ENSG00000240889	NDUFB2-AS1	4206	8.19E-04	6.64E-01	4.03E-04	0.3265116	0.0084325	0.3349441	0.0258259	5.00E-06
liberal	PD_mortality	psychencode	ENSG00000125743	SNRPD2	3752	2.60E-05	2.32E-01	8.52E-05	0.7595849	0.0080891	0.7676741	0.0106494	1.00E-05
robust	PD_mortality	psychencode	ENSG00000257643		4147	1.41E-03	3.66E-01	2.40E-03	0.6229006	0.0072828	0.6301835	0.0116918	5.00E-06
liberal	PD_mortality	psychencode	ENSG00000267191		3230	2.35E-03	1.80E-01	1.05E-02	0.8000156	0.0069749	0.8069905	0.0087185	1.00E-05
robust	PD_mortality	eQTLGen	ENSG00000269148		5467	5.09E-05	6.71E-01	2.45E-05	0.3222409	0.0067859	0.3290269	0.0210586	5.00E-06
robust	PD_mortality	psychencode	ENSG00000006459	KDM7A	2342	5.38E-04	1.17E-01	4.00E-03	0.8717416	0.0066274	0.8783690	0.0076025	5.00E-06
liberal	PD_mortality	psychencode	ENSG00000011478	QPCTL	3752	2.24E-05	2.00E-01	8.90E-05	0.7934213	0.0065772	0.7999984	0.0082897	1.00E-05
liberal	PD_mortality	eQTLGen	ENSG00000062370	ZNF112	5505	6.01E-05	7.92E-01	1.53E-05	0.2018508	0.0063935	0.2082443	0.0316746	1.00E-05
liberal	PD_mortality	psychencode	ENSG00000269955	LUC7L2	2867	2.06E-04	4.54E-02	4.28E-03	0.9438250	0.0062445	0.9500696	0.0066162	1.00E-05
robust	PD_mortality	psychencode	ENSG00000122778	KIAA1549	2945	7.83E-04	1.75E-01	3.65E-03	0.8152018	0.0055773	0.8207791	0.0068416	5.00E-06
liberal	PD_mortality	eQTLGen	ENSG00000259605		5438	1.45E-05	1.91E-01	6.10E-05	0.8041051	0.0052452	0.8093504	0.0065231	1.00E-05
robust	PD_mortality	eQTLGen	ENSG00000130204	TOMM40	5199	5.07E-06	6.67E-02	7.05E-05	0.9286983	0.0044773	0.9331756	0.0048210	5.00E-06

robust	PD_mortality	eQTLGen	ENSG00000130203	APOE	5224	6.75E-05	8.90E-01	8.03E-06	0.1057874	0.0042927	0.1100800	0.0405784	5.00E-06
liberal	PD_mortality	psychencode	ENSG00000139132	FGD4	4456	6.34E-10	7.54E-09	7.72E-02	0.9185712	0.0042371	0.9228083	0.0046127	1.00E-05
liberal	PD_mortality	psychencode	ENSG00000176222	ZNF404	3208	1.95E-78	6.59E-78	2.27E-01	0.7687265	0.0040727	0.7727992	0.0052979	1.00E-05
robust	PD_mortality	psychencode	ENSG00000125743	SNRPD2	3752	2.61E-05	2.33E-01	8.55E-05	0.7626696	0.0040610	0.7667306	0.0053247	5.00E-06
robust	PD_mortality	psychencode	ENSG00000267191		3230	2.36E-03	1.81E-01	1.05E-02	0.8028153	0.0034997	0.8063150	0.0043592	5.00E-06
robust	PD_mortality	psychencode	ENSG00000011478	QPCTL	3752	2.25E-05	2.01E-01	8.92E-05	0.7960391	0.0032994	0.7993386	0.0041448	5.00E-06
robust	PD_mortality	eQTLGen	ENSG00000062370	ZNF112	5505	6.03E-05	7.94E-01	1.54E-05	0.2024981	0.0032070	0.2057051	0.0158373	5.00E-06
robust	PD_mortality	psychencode	ENSG00000269955	LUC7L2	2867	2.07E-04	4.56E-02	4.29E-03	0.9467811	0.0031320	0.9499132	0.0033081	5.00E-06
liberal	PD_mortality	psychencode	ENSG00000224916	APOC4-APOC2	3336	8.82E-06	7.86E-02	1.03E-04	0.9181665	0.0030848	0.9212513	0.0033598	1.00E-05
robust	PD_mortality	eQTLGen	ENSG00000259605		5438	1.45E-05	1.91E-01	6.12E-05	0.8062196	0.0026295	0.8088491	0.0032615	5.00E-06
robust	PD_mortality	psychencode	ENSG00000139132	FGD4	4456	6.35E-10	7.56E-09	7.74E-02	0.9205214	0.0021230	0.9226445	0.0023064	5.00E-06
robust	PD_mortality	psychencode	ENSG00000176222	ZNF404	3208	1.95E-78	6.60E-78	2.28E-01	0.7702951	0.0020405	0.7723356	0.0026490	5.00E-06
liberal	PD_mortality	eQTLGen	ENSG00000267395		5420	4.21E-06	5.54E-02	7.15E-05	0.9427055	0.0017904	0.9444959	0.0018992	1.00E-05
robust	PD_mortality	psychencode	ENSG00000224916	APOC4-APOC2	3336	8.83E-06	7.88E-02	1.03E-04	0.9195848	0.0015448	0.9211296	0.0016799	5.00E-06
liberal	PD_mortality	psychencode	ENSG00000267022		3417	6.64E-07	5.92E-03	1.11E-04	0.9925240	0.0014420	0.9939660	0.0014529	1.00E-05
liberal	PD_mortality	eQTLGen	ENSG00000170684	ZNF296	5241	1.42E-05	1.87E-01	6.16E-05	0.8118766	0.0014389	0.8133156	0.0017724	1.00E-05
liberal	PD_mortality	psychencode	ENSG00000105939	ZC3HAV1	2979	6.24E-05	1.39E-02	4.39E-03	0.9802102	0.0014243	0.9816345	0.0014531	1.00E-05
liberal	PD_mortality	psychencode	ENSG00000236807		3399	4.13E-06	3.69E-02	1.08E-04	0.9617898	0.0012276	0.9630174	0.0012764	1.00E-05
liberal	PD_mortality	psychencode	ENSG00000176761	ZNF285B	3416	3.08E-06	2.75E-02	1.09E-04	0.9713194	0.0011068	0.9724262	0.0011395	1.00E-05
robust	PD_mortality	eQTLGen	ENSG00000267395		5420	4.21E-06	5.55E-02	7.16E-05	0.9435502	0.0008960	0.9444461	0.0009496	5.00E-06
liberal	PD_mortality	eQTLGen	ENSG00000124459	ZNF45	5715	7.30E-06	9.63E-02	6.84E-05	0.9027858	0.0007939	0.9035797	0.0008794	1.00E-05
liberal	PD_mortality	psychencode	ENSG00000125744	RTN2	3718	1.92E-06	1.71E-02	1.10E-04	0.9819939	0.0007932	0.9827871	0.0008078	1.00E-05
robust	PD_mortality	psychencode	ENSG00000267022		3417	6.64E-07	5.93E-03	1.11E-04	0.9932402	0.0007215	0.9939617	0.0007264	5.00E-06
robust	PD_mortality	eQTLGen	ENSG00000170684	ZNF296	5241	1.42E-05	1.87E-01	6.17E-05	0.8124612	0.0007200	0.8131812	0.0008862	5.00E-06
robust	PD_mortality	psychencode	ENSG00000105939	ZC3HAV1	2979	6.24E-05	1.39E-02	4.40E-03	0.9809087	0.0007127	0.9816214	0.0007265	5.00E-06
robust	PD_mortality	psychencode	ENSG00000236807		3399	4.14E-06	3.69E-02	1.08E-04	0.9623805	0.0006142	0.9629947	0.0006382	5.00E-06
liberal	PD_mortality	eQTLGen	ENSG00000125741	OPA3	5639	5.55E-06	7.32E-02	7.03E-05	0.9261552	0.0005998	0.9267549	0.0006476	1.00E-05
liberal	PD_mortality	eQTLGen	ENSG00000176222	ZNF404	5731	5.25E-98	1.60E-96	3.17E-02	0.9677501	0.0005656	0.9683157	0.0005845	1.00E-05
robust	PD_mortality	psychencode	ENSG00000176761	ZNF285B	3416	3.08E-06	2.75E-02	1.09E-04	0.9718572	0.0005537	0.9724110	0.0005698	5.00E-06
liberal	PD_mortality	eQTLGen	ENSG00000139132	FGD4	6454	2.20E-27	7.16E-25	3.06E-03	0.9963916	0.0005458	0.9969374	0.0005478	1.00E-05
robust	PD_mortality	eQTLGen	ENSG00000124459	ZNF45	5715	7.31E-06	9.64E-02	6.85E-05	0.9031443	0.0003971	0.9035415	0.0004397	5.00E-06
robust	PD_mortality	psychencode	ENSG00000125744	RTN2	3718	1.92E-06	1.71E-02	1.10E-04	0.9823835	0.0003968	0.9827803	0.0004039	5.00E-06
liberal	PD_mortality	eQTLGen	ENSG00000257511		6187	1.68E-06	6.95E-04	2.41E-03	0.9965574	0.0003390	0.9968963	0.0003401	1.00E-05
robust	PD_mortality	eQTLGen	ENSG00000125741	OPA3	5639	5.55E-06	7.32E-02	7.03E-05	0.9264330	0.0003000	0.9267329	0.0003238	5.00E-06

robust	PD_mortality	eQTLGen	ENSG00000176222	ZNF404	5731	5.25E-98	1.60E-96	3.17E-02	0.9680239	0.0002829	0.9683068	0.0002922	5.00E-06
liberal	PD_mortality	eQTLGen	ENSG00000258794		3993	3.72E-06	1.54E-03	2.40E-03	0.9957768	0.0002755	0.9960523	0.0002767	1.00E-05
robust	PD_mortality	eQTLGen	ENSG00000139132	FGD4	6454	2.20E-27	7.17E-25	3.06E-03	0.9966636	0.0002730	0.9969366	0.0002739	5.00E-06
liberal	PD_mortality	psychencode	ENSG00000090266	NDUFB2	2328	3.42E-09	7.37E-07	4.62E-03	0.9951230	0.0002531	0.9953761	0.0002543	1.00E-05
liberal	PD_mortality	eQTLGen	ENSG00000159885	ZNF222	5542	1.02E-06	1.34E-02	7.48E-05	0.9862726	0.0002450	0.9865176	0.0002484	1.00E-05
liberal	PD_mortality	psychencode	ENSG00000267058		3230	1.89E-23	1.45E-21	1.29E-02	0.9868956	0.0002124	0.9871080	0.0002152	1.00E-05
liberal	PD_mortality	eQTLGen	ENSG00000157741	UBN2	4973	1.87E-08	1.55E-05	1.21E-03	0.9985659	0.0002121	0.9987780	0.0002124	1.00E-05
liberal	PD_mortality	psychencode	ENSG00000256538		3403	2.46E-08	6.39E-06	3.84E-03	0.9959596	0.0001987	0.9961582	0.0001995	1.00E-05
liberal	PD_mortality	eQTLGen	ENSG00000139131	YARS2	6191	4.32E-07	1.79E-04	2.41E-03	0.9972145	0.0001975	0.9974120	0.0001981	1.00E-05
liberal	PD_mortality	psychencode	ENSG00000125755	SYMPK	3741	4.72E-07	4.21E-03	1.12E-04	0.9954810	0.0001971	0.9956781	0.0001980	1.00E-05
liberal	PD_mortality	psychencode	ENSG00000164898	C7orf55	2870	4.12E-06	9.10E-04	4.50E-03	0.9943872	0.0001942	0.9945814	0.0001953	1.00E-05
robust	PD_mortality	eQTLGen	ENSG00000257511		6187	1.68E-06	6.95E-04	2.41E-03	0.9967263	0.0001695	0.9968958	0.0001701	5.00E-06
liberal	PD_mortality	eQTLGen	ENSG00000146955	RAB19	4440	8.94E-06	7.30E-03	1.21E-03	0.9913085	0.0001668	0.9914753	0.0001683	1.00E-05
liberal	PD_mortality	psychencode	ENSG00000188883	KLRG2	2797	1.38E-11	3.02E-09	4.57E-03	0.9952720	0.0001627	0.9954347	0.0001635	1.00E-05
liberal	PD_mortality	psychencode	ENSG00000133597	ADCK2	2320	9.07E-27	1.95E-24	4.62E-03	0.9952198	0.0001565	0.9953763	0.0001573	1.00E-05
liberal	PD_mortality	psychencode	ENSG00000157800	SLC37A3	2491	5.55E-07	1.20E-04	4.60E-03	0.9951439	0.0001399	0.9952837	0.0001405	1.00E-05
robust	PD_mortality	eQTLGen	ENSG00000258794		3993	3.72E-06	1.54E-03	2.40E-03	0.9959139	0.0001378	0.9960517	0.0001383	5.00E-06
robust	PD_mortality	psychencode	ENSG00000090266	NDUFB2	2328	3.42E-09	7.37E-07	4.62E-03	0.9952490	0.0001265	0.9953755	0.0001272	5.00E-06
robust	PD_mortality	eQTLGen	ENSG00000159885	ZNF222	5542	1.02E-06	1.34E-02	7.48E-05	0.9863935	0.0001225	0.9865160	0.0001242	5.00E-06
liberal	PD_mortality	eQTLGen	ENSG00000267058		5719	1.08E-39	1.54E-37	6.99E-03	0.9928893	0.0001181	0.9930074	0.0001189	1.00E-05
liberal	PD_mortality	psychencode	ENSG00000139133	ALG10	3445	1.86E-10	4.83E-08	3.84E-03	0.9960510	0.0001132	0.9961643	0.0001137	1.00E-05
liberal	PD_mortality	psychencode	ENSG00000133606	MKRN1	2418	2.18E-06	4.70E-04	4.60E-03	0.9948167	0.0001064	0.9949231	0.0001069	1.00E-05
liberal	PD_mortality	psychencode	ENSG00000146966	DENND2A	2361	1.38E-15	2.98E-13	4.62E-03	0.9952781	0.0001063	0.9953844	0.0001068	1.00E-05
robust	PD_mortality	psychencode	ENSG00000267058		3230	1.89E-23	1.45E-21	1.29E-02	0.9870005	0.0001062	0.9871067	0.0001076	5.00E-06
robust	PD_mortality	eQTLGen	ENSG00000157741	UBN2	4973	1.87E-08	1.55E-05	1.21E-03	0.9986718	0.0001061	0.9987779	0.0001062	5.00E-06
liberal	PD_mortality	eQTLGen	ENSG00000159915	ZNF233	5599	9.77E-07	1.29E-02	7.49E-05	0.9869473	0.0001026	0.9870499	0.0001039	1.00E-05
liberal	PD_mortality	psychencode	ENSG00000259937		3947	7.30E-14	1.90E-11	3.84E-03	0.9960643	0.0001005	0.9961648	0.0001009	1.00E-05
liberal	PD_mortality	psychencode	ENSG00000260231	JHDM1D-AS1	2343	2.71E-18	5.91E-16	4.57E-03	0.9953285	0.0001001	0.9954286	0.0001006	1.00E-05
robust	PD_mortality	psychencode	ENSG00000256538		3403	2.46E-08	6.39E-06	3.84E-03	0.9960585	0.0000994	0.9961579	0.0000997	5.00E-06
robust	PD_mortality	eQTLGen	ENSG00000139131	YARS2	6191	4.32E-07	1.79E-04	2.41E-03	0.9973130	0.0000988	0.9974118	0.0000990	5.00E-06
liberal	PD_mortality	psychencode	ENSG00000256070		3964	4.11E-09	1.07E-06	3.84E-03	0.9960653	0.0000985	0.9961638	0.0000989	1.00E-05
robust	PD_mortality	psychencode	ENSG00000125755	SYMPK	3741	4.72E-07	4.21E-03	1.12E-04	0.9955791	0.0000985	0.9956776	0.0000990	5.00E-06
robust	PD_mortality	psychencode	ENSG00000164898	C7orf55	2870	4.12E-06	9.10E-04	4.50E-03	0.9944838	0.0000971	0.9945809	0.0000976	5.00E-06
liberal	PD_mortality	psychencode	ENSG00000110975	SYT10	3970	3.23E-46	8.38E-44	3.84E-03	0.9960703	0.0000946	0.9961649	0.0000949	1.00E-05

liberal	PD_mortality	psychencode	ENSG00000258794		3067	6.00E-17	1.56E-14	3.84E-03	0.9960697	0.0000933	0.9961631	0.0000937	1.00E-05
liberal	PD_mortality	psychencode	ENSG00000186026	ZNF284	3423	2.28E-07	2.03E-03	1.12E-04	0.9977648	0.0000901	0.9978549	0.0000903	1.00E-05
liberal	PD_mortality	eQTLGen	ENSG00000176761	ZNF285B	5417	4.44E-07	5.85E-03	7.55E-05	0.9939909	0.0000874	0.9940783	0.0000879	1.00E-05
robust	PD_mortality	eQTLGen	ENSG00000146955	RAB19	4440	8.94E-06	7.30E-03	1.21E-03	0.9913912	0.0000834	0.9914746	0.0000841	5.00E-06
robust	PD_mortality	psychencode	ENSG00000188883	KLRG2	2797	1.38E-11	3.02E-09	4.57E-03	0.9953530	0.0000814	0.9954343	0.0000817	5.00E-06
liberal	PD_mortality	eQTLGen	ENSG00000069399	BCL3	5346	4.62E-07	6.08E-03	7.54E-05	0.9937609	0.0000801	0.9938410	0.0000806	1.00E-05
liberal	PD_mortality	eQTLGen	ENSG00000146963	C7orf55-LUC7L2	5045	5.44E-07	4.47E-04	1.21E-03	0.9982588	0.0000796	0.9983384	0.0000797	1.00E-05
robust	PD_mortality	psychencode	ENSG00000133597	ADCK2	2320	9.07E-27	1.95E-24	4.62E-03	0.9952977	0.0000783	0.9953759	0.0000786	5.00E-06
liberal	PD_mortality	psychencode	ENSG00000062370	ZNF112	3509	2.09E-07	1.87E-03	1.12E-04	0.9979462	0.0000762	0.9980224	0.0000763	1.00E-05
liberal	PD_mortality	eQTLGen	ENSG00000139133	ALG10	4513	9.79E-45	4.06E-42	2.41E-03	0.9975212	0.0000717	0.9975929	0.0000719	1.00E-05
robust	PD_mortality	psychencode	ENSG00000157800	SLC37A3	2491	5.55E-07	1.20E-04	4.60E-03	0.9952134	0.0000699	0.9952834	0.0000703	5.00E-06
liberal	PD_mortality	eQTLGen	ENSG00000105939	ZC3HAV1	5168	8.05E-90	6.71E-87	1.20E-03	0.9987371	0.0000649	0.9988020	0.0000649	1.00E-05
liberal	PD_mortality	eQTLGen	ENSG00000133597	ADCK2	4199	1.82E-305	1.47E-302	1.23E-03	0.9987046	0.0000633	0.9987679	0.0000634	1.00E-05
liberal	PD_mortality	eQTLGen	ENSG00000105948	TTC26	5112	1.69E-161	1.41E-158	1.20E-03	0.9987379	0.0000614	0.9987993	0.0000615	1.00E-05
robust	PD_mortality	eQTLGen	ENSG00000267058		5719	1.08E-39	1.54E-37	6.99E-03	0.9929479	0.0000590	0.9930070	0.0000595	5.00E-06
robust	PD_mortality	psychencode	ENSG00000139133	ALG10	3445	1.86E-10	4.83E-08	3.84E-03	0.9961074	0.0000566	0.9961641	0.0000568	5.00E-06
liberal	PD_mortality	eQTLGen	ENSG00000087470	DNM1L	6223	1.24E-68	5.13E-66	2.41E-03	0.9975365	0.0000548	0.9975914	0.0000550	1.00E-05
liberal	PD_mortality	eQTLGen	ENSG00000133606	MKRN1	4384	1.12E-16	9.12E-14	1.22E-03	0.9987223	0.0000543	0.9987765	0.0000543	1.00E-05
liberal	PD_mortality	eQTLGen	ENSG00000164898	C7orf55	5034	1.92E-25	1.58E-22	1.21E-03	0.9987371	0.0000535	0.9987906	0.0000536	1.00E-05
liberal	PD_mortality	eQTLGen	ENSG00000260231	JHDM1D-AS1	4317	3.19E-88	2.61E-85	1.22E-03	0.9987258	0.0000533	0.9987791	0.0000533	1.00E-05
robust	PD_mortality	psychencode	ENSG00000133606	MKRN1	2418	2.18E-06	4.70E-04	4.60E-03	0.9948696	0.0000532	0.9949228	0.0000535	5.00E-06
robust	PD_mortality	psychencode	ENSG00000146966	DENND2A	2361	1.38E-15	2.98E-13	4.62E-03	0.9953310	0.0000532	0.9953842	0.0000534	5.00E-06
liberal	PD_mortality	eQTLGen	ENSG00000157764	BRAF	4190	7.08E-08	5.71E-05	1.24E-03	0.9986537	0.0000521	0.9987058	0.0000522	1.00E-05
robust	PD_mortality	eQTLGen	ENSG00000159915	ZNF233	5599	9.77E-07	1.29E-02	7.49E-05	0.9869979	0.0000513	0.9870492	0.0000520	5.00E-06
robust	PD_mortality	psychencode	ENSG00000259937		3947	7.30E-14	1.90E-11	3.84E-03	0.9961144	0.0000503	0.9961646	0.0000505	5.00E-06
robust	PD_mortality	psychencode	ENSG00000260231	JHDM1D-AS1	2343	2.71E-18	5.91E-16	4.57E-03	0.9953783	0.0000501	0.9954284	0.0000503	5.00E-06
robust	PD_mortality	psychencode	ENSG00000256070		3964	4.11E-09	1.07E-06	3.84E-03	0.9961144	0.0000493	0.9961636	0.0000495	5.00E-06
liberal	PD_mortality	eQTLGen	ENSG00000236279	CLEC2L	5074	1.30E-37	1.06E-34	1.22E-03	0.9987323	0.0000483	0.9987807	0.0000484	1.00E-05
robust	PD_mortality	psychencode	ENSG00000110975	SYT10	3970	3.23E-46	8.38E-44	3.84E-03	0.9961174	0.0000473	0.9961647	0.0000475	5.00E-06
robust	PD_mortality	psychencode	ENSG00000258794		3067	6.00E-17	1.56E-14	3.84E-03	0.9961162	0.0000467	0.9961629	0.0000469	5.00E-06
robust	PD_mortality	psychencode	ENSG00000186026	ZNF284	3423	2.28E-07	2.03E-03	1.12E-04	0.9978098	0.0000450	0.9978548	0.0000451	5.00E-06
robust	PD_mortality	eQTLGen	ENSG00000176761	ZNF285B	5417	4.44E-07	5.85E-03	7.55E-05	0.9940343	0.0000437	0.9940780	0.0000440	5.00E-06
robust	PD_mortality	eQTLGen	ENSG00000069399	BCL3	5346	4.62E-07	6.08E-03	7.54E-05	0.9938007	0.0000400	0.9938407	0.0000403	5.00E-06
robust	PD_mortality	eQTLGen	ENSG00000146963	C7orf55-LUC7L2	5045	5.44E-07	4.47E-04	1.21E-03	0.9982985	0.0000398	0.9983383	0.0000399	5.00E-06

robust	PD_mortality	psychencode	ENSG00000062370	ZNF112	3509	2.09E-07	1.87E-03	1.12E-04	0.9979842	0.0000381	0.9980223	0.0000382	5.00E-06
liberal	PD_mortality	psychencode	ENSG00000167383	ZNF229	3408	1.18E-07	1.05E-03	1.12E-04	0.9988014	0.0000368	0.9988383	0.0000369	1.00E-05
robust	PD_mortality	eQTLGen	ENSG00000139133	ALG10	4513	9.79E-45	4.06E-42	2.41E-03	0.9975570	0.0000359	0.9975928	0.0000360	5.00E-06
liberal	PD_mortality	eQTLGen	ENSG00000057294	PKP2	6166	1.35E-102	5.61E-100	2.41E-03	0.9975564	0.0000358	0.9975922	0.0000359	1.00E-05
liberal	PD_mortality	eQTLGen	ENSG00000170604	IRF2BP1	5372	3.16E-07	4.16E-03	7.56E-05	0.9957313	0.0000343	0.9957656	0.0000344	1.00E-05
robust	PD_mortality	eQTLGen	ENSG00000105939	ZC3HAV1	5168	8.05E-90	6.71E-87	1.20E-03	0.9987695	0.0000324	0.9988019	0.0000325	5.00E-06
robust	PD_mortality	eQTLGen	ENSG00000133597	ADCK2	4199	1.82E-305	1.47E-302	1.23E-03	0.9987362	0.0000317	0.9987679	0.0000317	5.00E-06
robust	PD_mortality	eQTLGen	ENSG00000105948	TTC26	5112	1.69E-161	1.41E-158	1.20E-03	0.9987686	0.0000307	0.9987993	0.0000307	5.00E-06
liberal	PD_mortality	psychencode	ENSG00000104936	DMPK	3720	1.52E-08	1.36E-04	1.12E-04	0.9997233	0.0000286	0.9997519	0.0000286	1.00E-05
liberal	PD_mortality	eQTLGen	ENSG00000059377	TBXAS1	4674	3.06E-263	2.51E-260	1.22E-03	0.9987544	0.0000278	0.9987822	0.0000279	1.00E-05
robust	PD_mortality	eQTLGen	ENSG00000087470	DNM1L	6223	1.24E-68	5.13E-66	2.41E-03	0.9975639	0.0000274	0.9975913	0.0000275	5.00E-06
robust	PD_mortality	eQTLGen	ENSG00000133606	MKRN1	4384	1.12E-16	9.12E-14	1.22E-03	0.9987494	0.0000271	0.9987765	0.0000272	5.00E-06
robust	PD_mortality	eQTLGen	ENSG00000164898	C7orf55	5034	1.92E-25	1.58E-22	1.21E-03	0.9987638	0.0000268	0.9987906	0.0000268	5.00E-06
robust	PD_mortality	eQTLGen	ENSG00000260231	JHDM1D-AS1	4317	3.19E-88	2.61E-85	1.22E-03	0.9987524	0.0000266	0.9987791	0.0000267	5.00E-06
robust	PD_mortality	eQTLGen	ENSG00000157764	BRAF	4190	7.08E-08	5.71E-05	1.24E-03	0.9986797	0.0000261	0.9987058	0.0000261	5.00E-06
robust	PD_mortality	eQTLGen	ENSG00000236279	CLEC2L	5074	1.30E-37	1.06E-34	1.22E-03	0.9987565	0.0000242	0.9987806	0.0000242	5.00E-06
liberal	PD_mortality	eQTLGen	ENSG00000090266	NDUFB2	4212	6.40E-306	5.18E-303	1.23E-03	0.9987441	0.0000235	0.9987676	0.0000235	1.00E-05
liberal	PD_mortality	eQTLGen	ENSG00000157800	SLC37A3	4502	1.48E-299	1.21E-296	1.22E-03	0.9987521	0.0000229	0.9987750	0.0000229	1.00E-05
liberal	PD_mortality	psychencode	ENSG00000167384	ZNF180	3427	6.31E-22	5.63E-18	1.12E-04	0.9998653	0.0000226	0.9998879	0.0000226	1.00E-05
liberal	PD_mortality	eQTLGen	ENSG00000064393	HIPK2	5038	7.77E-15	6.35E-12	1.22E-03	0.9987559	0.0000226	0.9987784	0.0000226	1.00E-05
liberal	PD_mortality	eQTLGen	ENSG00000229677		5107	9.73E-14	8.11E-11	1.20E-03	0.9987803	0.0000221	0.9988024	0.0000221	1.00E-05
liberal	PD_mortality	eQTLGen	ENSG00000104856	RELB	5188	9.30E-09	1.23E-04	7.59E-05	0.9997802	0.0000214	0.9998016	0.0000214	1.00E-05
liberal	PD_mortality	psychencode	ENSG00000125746	EML2	3763	5.74E-08	5.12E-04	1.12E-04	0.9993571	0.0000189	0.9993760	0.0000189	1.00E-05
robust	PD_mortality	psychencode	ENSG00000167383	ZNF229	3408	1.18E-07	1.05E-03	1.12E-04	0.9988198	0.0000184	0.9988383	0.0000184	5.00E-06
robust	PD_mortality	eQTLGen	ENSG00000057294	PKP2	6166	1.35E-102	5.61E-100	2.41E-03	0.9975743	0.0000179	0.9975922	0.0000179	5.00E-06
liberal	PD_mortality	eQTLGen	ENSG00000178386	ZNF223	5499	4.22E-66	5.57E-62	7.59E-05	0.9999064	0.0000178	0.9999241	0.0000178	1.00E-05
liberal	PD_mortality	eQTLGen	ENSG00000186026	ZNF284	5425	9.96E-34	1.31E-29	7.59E-05	0.9999064	0.0000177	0.9999241	0.0000177	1.00E-05
robust	PD_mortality	eQTLGen	ENSG00000170604	IRF2BP1	5372	3.16E-07	4.16E-03	7.56E-05	0.9957484	0.0000171	0.9957655	0.0000172	5.00E-06
liberal	PD_mortality	eQTLGen	ENSG00000146858	ZC3HAV1L	5105	1.17E-21	9.78E-19	1.20E-03	0.9987865	0.0000164	0.9988030	0.0000164	1.00E-05
liberal	PD_mortality	eQTLGen	ENSG00000059378	PARP12	4448	9.93E-27	8.12E-24	1.22E-03	0.9987631	0.0000159	0.9987791	0.0000160	1.00E-05
liberal	PD_mortality	eQTLGen	ENSG00000006459	KDM7A	4370	8.63E-43	7.06E-40	1.22E-03	0.9987641	0.0000154	0.9987795	0.0000154	1.00E-05
liberal	PD_mortality	psychencode	ENSG00000159882	ZNF230	3387	4.12E-12	3.67E-08	1.12E-04	0.9998728	0.0000151	0.9998879	0.0000151	1.00E-05
robust	PD_mortality	psychencode	ENSG00000104936	DMPK	3720	1.52E-08	1.36E-04	1.12E-04	0.9997376	0.0000143	0.9997519	0.0000143	5.00E-06
robust	PD_mortality	eQTLGen	ENSG00000059377	TBXAS1	4674	3.06E-263	2.51E-260	1.22E-03	0.9987683	0.0000139	0.9987822	0.0000139	5.00E-06

liberal	PD_mortality	eQTLGen	ENSG00000267680	ZNF224	5455	4.37E-91	5.75E-87	7.59E-05	0.9999121	0.0000120	0.9999241	0.0000120	1.00E-05
robust	PD_mortality	eQTLGen	ENSG00000090266	NDUFB2	4212	6.40E-306	5.18E-303	1.23E-03	0.9987558	0.0000117	0.9987676	0.0000118	5.00E-06
robust	PD_mortality	eQTLGen	ENSG00000157800	SLC37A3	4502	1.48E-299	1.21E-296	1.23E-03	0.9987635	0.0000114	0.9987750	0.0000115	5.00E-06
robust	PD_mortality	psychencode	ENSG00000167384	ZNF180	3427	6.31E-22	5.63E-18	1.12E-04	0.9998766	0.0000113	0.9998879	0.0000113	5.00E-06
robust	PD_mortality	eQTLGen	ENSG00000064393	HIPK2	5038	7.77E-15	6.35E-12	1.22E-03	0.9987671	0.0000113	0.9987784	0.0000113	5.00E-06
robust	PD_mortality	eQTLGen	ENSG00000229677		5107	9.73E-14	8.11E-11	1.20E-03	0.9987913	0.0000110	0.9988024	0.0000111	5.00E-06
robust	PD_mortality	eQTLGen	ENSG00000104856	RELB	5188	9.30E-09	1.23E-04	7.59E-05	0.9997909	0.0000107	0.9998016	0.0000107	5.00E-06
robust	PD_mortality	psychencode	ENSG00000125746	EML2	3763	5.74E-08	5.12E-04	1.12E-04	0.9993665	0.0000094	0.9993759	0.0000094	5.00E-06
robust	PD_mortality	eQTLGen	ENSG00000178386	ZNF223	5499	4.22E-66	5.57E-62	7.59E-05	0.9999153	0.0000089	0.9999241	0.0000089	5.00E-06
robust	PD_mortality	eQTLGen	ENSG00000186026	ZNF284	5425	9.96E-34	1.31E-29	7.59E-05	0.9999153	0.0000089	0.9999241	0.0000089	5.00E-06
liberal	PD_mortality	psychencode	ENSG00000170604	IRF2BP1	3639	9.32E-09	8.31E-05	1.12E-04	0.9997959	0.0000089	0.9998048	0.0000089	1.00E-05
liberal	PD_mortality	eQTLGen	ENSG00000125746	EML2	5603	1.59E-10	2.09E-06	7.59E-05	0.9999134	0.0000086	0.9999220	0.0000086	1.00E-05
liberal	PD_mortality	psychencode	ENSG00000186567	CEACAM19	3485	6.61E-10	5.89E-06	1.12E-04	0.9998734	0.0000086	0.9998820	0.0000086	1.00E-05
robust	PD_mortality	eQTLGen	ENSG00000146858	ZC3HAV1L	5105	1.17E-21	9.78E-19	1.20E-03	0.9987947	0.0000082	0.9988030	0.0000082	5.00E-06
robust	PD_mortality	eQTLGen	ENSG00000059378	PARP12	4448	9.93E-27	8.12E-24	1.22E-03	0.9987711	0.0000080	0.9987791	0.0000080	5.00E-06
liberal	PD_mortality	psychencode	ENSG00000130202	PVRL2	3285	2.33E-26	2.08E-22	1.12E-04	0.9998799	0.0000080	0.9998879	0.0000080	1.00E-05
robust	PD_mortality	eQTLGen	ENSG00000006459	KDM7A	4370	8.63E-43	7.06E-40	1.22E-03	0.9987718	0.0000077	0.9987795	0.0000077	5.00E-06
robust	PD_mortality	psychencode	ENSG00000159882	ZNF230	3387	4.12E-12	3.67E-08	1.12E-04	0.9998804	0.0000075	0.9998879	0.0000075	5.00E-06
liberal	PD_mortality	eQTLGen	ENSG00000167383	ZNF229	5467	3.37E-08	4.44E-04	7.59E-05	0.9994723	0.0000074	0.9994797	0.0000074	1.00E-05
liberal	PD_mortality	eQTLGen	ENSG00000167380	ZNF226	5419	1.04E-22	1.37E-18	7.59E-05	0.9999176	0.0000065	0.9999241	0.0000065	1.00E-05
liberal	PD_mortality	eQTLGen	ENSG00000267242		5430	1.92E-36	2.53E-32	7.59E-05	0.9999177	0.0000064	0.9999241	0.0000064	1.00E-05
liberal	PD_mortality	psychencode	ENSG00000266903		3411	1.08E-82	9.67E-79	1.12E-04	0.9998818	0.0000061	0.9998879	0.0000061	1.00E-05
robust	PD_mortality	eQTLGen	ENSG00000267680	ZNF224	5455	4.37E-91	5.75E-87	7.59E-05	0.9999181	0.0000060	0.9999241	0.0000060	5.00E-06
liberal	PD_mortality	eQTLGen	ENSG00000256294	ZNF225	5442	1.46E-13	1.92E-09	7.59E-05	0.9999184	0.0000057	0.9999241	0.0000057	1.00E-05
liberal	PD_mortality	eQTLGen	ENSG00000186567	CEACAM19	5491	6.03E-38	7.94E-34	7.59E-05	0.9999187	0.0000054	0.9999241	0.0000054	1.00E-05
liberal	PD_mortality	eQTLGen	ENSG00000159882	ZNF230	5565	3.89E-21	5.13E-17	7.58E-05	0.9999193	0.0000049	0.9999242	0.0000049	1.00E-05
liberal	PD_mortality	psychencode	ENSG00000124459	ZNF45	3267	8.33E-09	7.43E-05	1.12E-04	0.9998090	0.0000046	0.9998136	0.0000046	1.00E-05
liberal	PD_mortality	eQTLGen	ENSG00000131115	ZNF227	5518	2.36E-32	3.11E-28	7.59E-05	0.9999196	0.0000045	0.9999241	0.0000045	1.00E-05
robust	PD_mortality	psychencode	ENSG00000170604	IRF2BP1	3639	9.32E-09	8.31E-05	1.12E-04	0.9998003	0.0000044	0.9998048	0.0000044	5.00E-06
robust	PD_mortality	eQTLGen	ENSG00000125746	EML2	5603	1.59E-10	2.09E-06	7.59E-05	0.9999177	0.0000043	0.9999220	0.0000043	5.00E-06
robust	PD_mortality	psychencode	ENSG00000186567	CEACAM19	3485	6.61E-10	5.89E-06	1.12E-04	0.9998777	0.0000043	0.9998820	0.0000043	5.00E-06
liberal	PD_mortality	eQTLGen	ENSG00000159917	ZNF235	5591	8.04E-48	1.06E-43	7.59E-05	0.9999199	0.0000042	0.9999241	0.0000042	1.00E-05
robust	PD_mortality	psychencode	ENSG00000130202	PVRL2	3285	2.33E-26	2.08E-22	1.12E-04	0.9998839	0.0000040	0.9998879	0.0000040	5.00E-06
robust	PD_mortality	eQTLGen	ENSG00000167383	ZNF229	5467	3.37E-08	4.44E-04	7.59E-05	0.9994760	0.0000037	0.9994797	0.0000037	5.00E-06

liberal	PD_mortality	psychencode	ENSG00000159915	ZNF233	3606	8.72E-11	7.77E-07	1.12E-04	0.9998835	0.0000036	0.9998871	0.0000036	1.00E-05
liberal	PD_mortality	eQTLGen	ENSG00000104853	CLPTM1	5189	2.37E-12	3.12E-08	7.59E-05	0.9999206	0.0000035	0.9999241	0.0000035	1.00E-05
liberal	PD_mortality	psychencode	ENSG00000010310	GIPR	3765	1.49E-09	1.33E-05	1.12E-04	0.9998711	0.0000035	0.9998746	0.0000035	1.00E-05
liberal	PD_mortality	psychencode	ENSG00000159905	ZNF221	3303	4.29E-09	3.83E-05	1.12E-04	0.9998462	0.0000034	0.9998496	0.0000034	1.00E-05
liberal	PD_mortality	psychencode	ENSG00000104892	KLC3	3744	1.79E-34	1.60E-30	1.12E-04	0.9998846	0.0000033	0.9998879	0.0000033	1.00E-05
robust	PD_mortality	eQTLGen	ENSG00000167380	ZNF226	5419	1.04E-22	1.37E-18	7.59E-05	0.9999208	0.0000033	0.9999241	0.0000033	5.00E-06
liberal	PD_mortality	eQTLGen	ENSG00000011478	QPCTL	5580	1.64E-28	2.16E-24	7.59E-05	0.9999209	0.0000033	0.9999241	0.0000033	1.00E-05
robust	PD_mortality	eQTLGen	ENSG00000267242		5430	1.92E-36	2.53E-32	7.59E-05	0.9999209	0.0000032	0.9999241	0.0000032	5.00E-06
robust	PD_mortality	psychencode	ENSG00000266903		3411	1.08E-82	9.67E-79	1.12E-04	0.9998848	0.0000031	0.9998879	0.0000031	5.00E-06
liberal	PD_mortality	psychencode	ENSG00000204920	ZNF155	3332	9.54E-21	8.51E-17	1.12E-04	0.9998850	0.0000029	0.9998879	0.0000029	1.00E-05
robust	PD_mortality	eQTLGen	ENSG00000256294	ZNF225	5442	1.46E-13	1.92E-09	7.59E-05	0.9999212	0.0000029	0.9999241	0.0000029	5.00E-06
liberal	PD_mortality	eQTLGen	ENSG00000176182	MYPOP	5353	4.07E-36	5.30E-32	7.67E-05	0.9999205	0.0000028	0.9999233	0.0000028	1.00E-05
robust	PD_mortality	eQTLGen	ENSG00000186567	CEACAM19	5491	6.03E-38	7.94E-34	7.59E-05	0.9999214	0.0000027	0.9999241	0.0000027	5.00E-06
liberal	PD_mortality	psychencode	ENSG00000267508	ZNF285	3480	7.85E-10	7.00E-06	1.12E-04	0.9998782	0.0000027	0.9998809	0.0000027	1.00E-05
liberal	PD_mortality	psychencode	ENSG00000189114	BLOC1S3	3492	2.39E-10	2.13E-06	1.12E-04	0.9998832	0.0000025	0.9998858	0.0000025	1.00E-05
robust	PD_mortality	eQTLGen	ENSG00000159882	ZNF230	5565	3.89E-21	5.13E-17	7.58E-05	0.9999217	0.0000025	0.9999242	0.0000025	5.00E-06
liberal	PD_mortality	psychencode	ENSG00000234906	APOC2	3324	2.29E-09	2.04E-05	1.12E-04	0.9998651	0.0000023	0.9998675	0.0000023	1.00E-05
robust	PD_mortality	psychencode	ENSG00000124459	ZNF45	3267	8.33E-09	7.43E-05	1.12E-04	0.9998113	0.0000023	0.9998136	0.0000023	5.00E-06
robust	PD_mortality	eQTLGen	ENSG00000131115	ZNF227	5518	2.36E-32	3.11E-28	7.59E-05	0.9999218	0.0000023	0.9999241	0.0000023	5.00E-06
liberal	PD_mortality	psychencode	ENSG00000142252	GEMIN7	3336	6.18E-14	5.51E-10	1.12E-04	0.9998857	0.0000022	0.9998879	0.0000022	1.00E-05
liberal	PD_mortality	eQTLGen	ENSG00000125755	SYMPK	5498	3.87E-59	5.09E-55	7.59E-05	0.9999220	0.0000021	0.9999241	0.0000021	1.00E-05
robust	PD_mortality	eQTLGen	ENSG00000159917	ZNF235	5591	8.04E-48	1.06E-43	7.59E-05	0.9999220	0.0000021	0.9999241	0.0000021	5.00E-06
liberal	PD_mortality	psychencode	ENSG00000125741	OPA3	3772	1.46E-10	1.30E-06	1.12E-04	0.9998845	0.0000021	0.9998866	0.0000021	1.00E-05
liberal	PD_mortality	eQTLGen	ENSG00000073008	PVR	5465	1.01E-47	1.33E-43	7.59E-05	0.9999222	0.0000019	0.9999241	0.0000019	1.00E-05
liberal	PD_mortality	eQTLGen	ENSG00000267508	ZNF285	5499	1.08E-10	1.43E-06	7.59E-05	0.9999207	0.0000019	0.9999227	0.0000019	1.00E-05
robust	PD_mortality	psychencode	ENSG00000159915	ZNF233	3606	8.72E-11	7.77E-07	1.12E-04	0.9998853	0.0000018	0.9998871	0.0000018	5.00E-06
liberal	PD_mortality	psychencode	ENSG00000073008	PVR	3449	2.13E-56	1.90E-52	1.12E-04	0.9998861	0.0000018	0.9998879	0.0000018	1.00E-05
liberal	PD_mortality	eQTLGen	ENSG00000104892	KLC3	5693	1.32E-141	1.75E-137	7.59E-05	0.9999223	0.0000018	0.9999241	0.0000018	1.00E-05
liberal	PD_mortality	eQTLGen	ENSG00000104866	PPP1R37	5334	1.80E-83	2.37E-79	7.59E-05	0.9999223	0.0000018	0.9999241	0.0000018	1.00E-05
robust	PD_mortality	eQTLGen	ENSG00000104853	CLPTM1	5189	2.37E-12	3.12E-08	7.59E-05	0.9999223	0.0000017	0.9999241	0.0000017	5.00E-06
robust	PD_mortality	psychencode	ENSG00000010310	GIPR	3765	1.49E-09	1.33E-05	1.12E-04	0.9998729	0.0000017	0.9998746	0.0000017	5.00E-06
liberal	PD_mortality	eQTLGen	ENSG00000007255	TRAPPC6A	5368	2.19E-152	2.88E-148	7.59E-05	0.9999224	0.0000017	0.9999241	0.0000017	1.00E-05
liberal	PD_mortality	eQTLGen	ENSG00000010310	GIPR	5620	2.17E-12	2.85E-08	7.59E-05	0.9999224	0.0000017	0.9999241	0.0000017	1.00E-05
robust	PD_mortality	psychencode	ENSG00000159905	ZNF221	3303	4.29E-09	3.83E-05	1.12E-04	0.9998479	0.0000017	0.9998496	0.0000017	5.00E-06

liberal	PD_mortality	eQTLGen	ENSG00000012061	ERCC1	5598	4.62E-233	6.09E-229	7.59E-05	0.9999225	0.0000017	0.9999241	0.0000017	1.00E-05
robust	PD_mortality	psychencode	ENSG00000104892	KLC3	3744	1.79E-34	1.60E-30	1.12E-04	0.9998862	0.0000017	0.9998879	0.0000017	5.00E-06
robust	PD_mortality	eQTLGen	ENSG00000011478	QPCTL	5580	1.64E-28	2.16E-24	7.59E-05	0.9999225	0.0000016	0.9999241	0.0000016	5.00E-06
liberal	PD_mortality	eQTLGen	ENSG00000179846	NKPD1	5341	8.60E-25	1.13E-20	7.59E-05	0.9999225	0.0000016	0.9999241	0.0000016	1.00E-05
liberal	PD_mortality	eQTLGen	ENSG00000189114	BLOC1S3	5385	6.55E-45	8.63E-41	7.59E-05	0.9999225	0.0000016	0.9999241	0.0000016	1.00E-05
liberal	PD_mortality	eQTLGen	ENSG00000125744	RTN2	5645	9.68E-82	1.28E-77	7.59E-05	0.9999225	0.0000016	0.9999241	0.0000016	1.00E-05
liberal	PD_mortality	eQTLGen	ENSG00000142252	GEMIN7	5285	7.90E-45	1.04E-40	7.59E-05	0.9999226	0.0000015	0.9999241	0.0000015	1.00E-05
liberal	PD_mortality	eQTLGen	ENSG00000204920	ZNF155	5576	5.22E-305	6.89E-301	7.58E-05	0.9999227	0.0000015	0.9999242	0.0000015	1.00E-05
liberal	PD_mortality	psychencode	ENSG00000213889	PPM1N	3737	2.42E-14	2.15E-10	1.12E-04	0.9998864	0.0000015	0.9998879	0.0000015	1.00E-05
robust	PD_mortality	psychencode	ENSG00000204920	ZNF155	3332	9.54E-21	8.51E-17	1.12E-04	0.9998865	0.0000015	0.9998879	0.0000015	5.00E-06
robust	PD_mortality	eQTLGen	ENSG00000176182	MYPOP	5353	4.07E-36	5.30E-32	7.67E-05	0.9999219	0.0000014	0.9999233	0.0000014	5.00E-06
liberal	PD_mortality	eQTLGen	ENSG00000177051	FBXO46	5544	1.35E-13	1.78E-09	7.59E-05	0.9999227	0.0000014	0.9999241	0.0000014	1.00E-05
liberal	PD_mortality	eQTLGen	ENSG00000104881	PPP1R13L	5624	3.36E-09	4.42E-05	7.59E-05	0.9998785	0.0000014	0.9998799	0.0000014	1.00E-05
liberal	PD_mortality	eQTLGen	ENSG00000230666	CEACAM22P	5422	5.31E-181	7.00E-177	7.59E-05	0.9999227	0.0000014	0.9999241	0.0000014	1.00E-05
liberal	PD_mortality	eQTLGen	ENSG00000187244	BCAM	5226	8.65E-15	1.14E-10	7.59E-05	0.9999227	0.0000014	0.9999241	0.0000014	1.00E-05
robust	PD_mortality	psychencode	ENSG00000267508	ZNF285	3480	7.85E-10	7.00E-06	1.12E-04	0.9998795	0.0000013	0.9998809	0.0000013	5.00E-06
liberal	PD_mortality	eQTLGen	ENSG00000117877	CD3EAP	5585	3.02E-33	3.98E-29	7.59E-05	0.9999228	0.0000013	0.9999241	0.0000013	1.00E-05
liberal	PD_mortality	eQTLGen	ENSG00000130202	PVRL2	5175	4.01E-308	5.29E-304	7.59E-05	0.9999228	0.0000013	0.9999241	0.0000013	1.00E-05
robust	PD_mortality	psychencode	ENSG00000189114	BLOC1S3	3492	2.39E-10	2.13E-06	1.12E-04	0.9998845	0.0000013	0.9998858	0.0000013	5.00E-06
liberal	PD_mortality	eQTLGen	ENSG00000104936	DMPK	5501	5.28E-283	6.96E-279	7.59E-05	0.9999229	0.0000013	0.9999241	0.0000013	1.00E-05
liberal	PD_mortality	eQTLGen	ENSG00000125743	SNRPD2	5604	1.01E-215	1.33E-211	7.59E-05	0.9999229	0.0000013	0.9999241	0.0000013	1.00E-05
liberal	PD_mortality	eQTLGen	ENSG00000185800	DMWD	5511	5.42E-208	7.15E-204	7.59E-05	0.9999229	0.0000013	0.9999241	0.0000013	1.00E-05
liberal	PD_mortality	eQTLGen	ENSG00000104879	CKM	5691	1.79E-226	2.36E-222	7.59E-05	0.9999229	0.0000012	0.9999241	0.0000012	1.00E-05
liberal	PD_mortality	eQTLGen	ENSG00000263002	ZNF234	5431	9.21E-308	1.21E-303	7.59E-05	0.9999229	0.0000012	0.9999241	0.0000012	1.00E-05
liberal	PD_mortality	eQTLGen	ENSG00000177045	SIX5	5468	6.67E-61	8.78E-57	7.59E-05	0.9999229	0.0000012	0.9999241	0.0000012	1.00E-05
liberal	PD_mortality	eQTLGen	ENSG00000104859	CLASRP	5179	4.95E-15	6.52E-11	7.59E-05	0.9999229	0.0000012	0.9999241	0.0000012	1.00E-05
liberal	PD_mortality	eQTLGen	ENSG00000130201	EXOC3L2	5429	7.83E-15	1.03E-10	7.59E-05	0.9999229	0.0000012	0.9999241	0.0000012	1.00E-05
robust	PD_mortality	psychencode	ENSG00000234906	APOC2	3324	2.29E-09	2.04E-05	1.12E-04	0.9998663	0.0000012	0.9998675	0.0000012	5.00E-06
liberal	PD_mortality	eQTLGen	ENSG00000007047	MARK4	5388	1.97E-307	2.60E-303	7.59E-05	0.9999230	0.0000011	0.9999241	0.0000011	1.00E-05
robust	PD_mortality	psychencode	ENSG00000142252	GEMIN7	3336	6.18E-14	5.51E-10	1.12E-04	0.9998868	0.0000011	0.9998879	0.0000011	5.00E-06
liberal	PD_mortality	eQTLGen	ENSG00000216588	IGSF23	5439	2.48E-26	3.26E-22	7.59E-05	0.9999230	0.0000011	0.9999241	0.0000011	1.00E-05
liberal	PD_mortality	eQTLGen	ENSG00000125753	VASP	5638	9.18E-308	1.21E-303	7.59E-05	0.9999230	0.0000011	0.9999241	0.0000011	1.00E-05
liberal	PD_mortality	eQTLGen	ENSG00000213889	PPM1N	5610	6.95E-96	9.16E-92	7.59E-05	0.9999231	0.0000011	0.9999241	0.0000011	1.00E-05
robust	PD_mortality	eQTLGen	ENSG00000125755	SYMPK	5498	3.87E-59	5.09E-55	7.59E-05	0.9999231	0.0000011	0.9999241	0.0000011	5.00E-06

robust	PD_mortality	psychencode	ENSG00000125741	OPA3	3772	1.46E-10	1.30E-06	1.12E-04	0.9998856	0.0000010	0.9998866	0.0000010	5.00E-06
liberal	PD_mortality	eQTLGen	ENSG00000267348		5238	1.24E-19	1.63E-15	7.59E-05	0.9999231	0.0000010	0.9999241	0.0000010	1.00E-05
liberal	PD_mortality	eQTLGen	ENSG00000125740	FOSB	5629	1.69E-11	2.23E-07	7.59E-05	0.9999229	0.0000010	0.9999239	0.0000010	1.00E-05
robust	PD_mortality	eQTLGen	ENSG00000073008	PVR	5465	1.01E-47	1.33E-43	7.59E-05	0.9999231	0.0000010	0.9999241	0.0000010	5.00E-06
robust	PD_mortality	eQTLGen	ENSG00000267508	ZNF285	5499	1.08E-10	1.43E-06	7.59E-05	0.9999217	0.0000010	0.9999227	0.0000010	5.00E-06
liberal	PD_mortality	eQTLGen	ENSG00000104884	ERCC2	5662	1.93E-90	2.55E-86	7.59E-05	0.9999232	0.0000009	0.9999241	0.0000009	1.00E-05
robust	PD_mortality	psychencode	ENSG00000073008	PVR	3449	2.13E-56	1.90E-52	1.12E-04	0.9998870	0.0000009	0.9998879	0.0000009	5.00E-06
robust	PD_mortality	eQTLGen	ENSG00000104892	KLC3	5693	1.32E-141	1.75E-137	7.59E-05	0.9999232	0.0000009	0.9999241	0.0000009	5.00E-06
robust	PD_mortality	eQTLGen	ENSG00000104866	PPP1R37	5334	1.80E-83	2.37E-79	7.59E-05	0.9999232	0.0000009	0.9999241	0.0000009	5.00E-06
robust	PD_mortality	eQTLGen	ENSG00000007255	TRAPPC6A	5368	2.19E-152	2.88E-148	7.59E-05	0.9999232	0.0000009	0.9999241	0.0000009	5.00E-06
robust	PD_mortality	eQTLGen	ENSG00000010310	GIPR	5620	2.17E-12	2.85E-08	7.59E-05	0.9999232	0.0000008	0.9999241	0.0000008	5.00E-06
robust	PD_mortality	eQTLGen	ENSG00000012061	ERCC1	5598	4.62E-233	6.09E-229	7.59E-05	0.9999233	0.0000008	0.9999241	0.0000008	5.00E-06
robust	PD_mortality	eQTLGen	ENSG00000179846	NKPD1	5341	8.60E-25	1.13E-20	7.59E-05	0.9999233	0.0000008	0.9999241	0.0000008	5.00E-06
robust	PD_mortality	eQTLGen	ENSG00000189114	BLOC1S3	5385	6.55E-45	8.64E-41	7.59E-05	0.9999233	0.0000008	0.9999241	0.0000008	5.00E-06
robust	PD_mortality	eQTLGen	ENSG00000125744	RTN2	5645	9.68E-82	1.28E-77	7.59E-05	0.9999233	0.0000008	0.9999241	0.0000008	5.00E-06
robust	PD_mortality	eQTLGen	ENSG00000142252	GEMIN7	5285	7.90E-45	1.04E-40	7.59E-05	0.9999233	0.0000008	0.9999241	0.0000008	5.00E-06
robust	PD_mortality	eQTLGen	ENSG00000204920	ZNF155	5576	5.22E-305	6.89E-301	7.58E-05	0.9999234	0.0000008	0.9999242	0.0000008	5.00E-06
robust	PD_mortality	psychencode	ENSG00000213889	PPM1N	3737	2.42E-14	2.15E-10	0.000112097	0.9998871	0.0000008	0.9998879	0.0000008	5.00E-06
robust	PD_mortality	eQTLGen	ENSG00000177051	FBXO46	5544	1.35E-13	1.78E-09	7.59E-05	0.9999234	0.0000007	0.9999241	0.0000007	5.00E-06
robust	PD_mortality	eQTLGen	ENSG00000104881	PPP1R13L	5624	3.36E-09	4.42E-05	7.59E-05	0.9998792	0.0000007	0.9998799	0.0000007	5.00E-06
robust	PD_mortality	eQTLGen	ENSG00000230666	CEACAM22P	5422	5.31E-181	7.00E-177	7.59E-05	0.9999234	0.0000007	0.9999241	0.0000007	5.00E-06
robust	PD_mortality	eQTLGen	ENSG00000187244	BCAM	5226	8.65E-15	1.14E-10	7.59E-05	0.9999234	0.0000007	0.9999241	0.0000007	5.00E-06
robust	PD_mortality	eQTLGen	ENSG00000117877	CD3EAP	5585	3.02E-33	3.98E-29	7.59E-05	0.9999235	0.0000007	0.9999241	0.0000007	5.00E-06
robust	PD_mortality	eQTLGen	ENSG00000130202	PVRL2	5175	4.01E-308	5.29E-304	7.59E-05	0.9999234	0.0000007	0.9999241	0.0000007	5.00E-06
robust	PD_mortality	eQTLGen	ENSG00000104936	DMPK	5501	5.28E-283	6.96E-279	7.59E-05	0.9999235	0.0000006	0.9999241	0.0000006	5.00E-06
robust	PD_mortality	eQTLGen	ENSG00000125743	SNRPD2	5604	1.01E-215	1.33E-211	7.59E-05	0.9999235	0.0000006	0.9999241	0.0000006	5.00E-06
robust	PD_mortality	eQTLGen	ENSG00000185800	DMWD	5511	5.42E-208	7.15E-204	7.59E-05	0.9999235	0.0000006	0.9999241	0.0000006	5.00E-06
robust	PD_mortality	eQTLGen	ENSG00000104879	CKM	5691	1.79E-226	2.36E-222	7.59E-05	0.9999235	0.0000006	0.9999241	0.0000006	5.00E-06
robust	PD_mortality	eQTLGen	ENSG00000263002	ZNF234	5431	9.21E-308	1.21E-303	7.59E-05	0.9999235	0.0000006	0.9999241	0.0000006	5.00E-06
robust	PD_mortality	eQTLGen	ENSG00000177045	SIX5	5468	6.67E-61	8.78E-57	7.59E-05	0.9999235	0.0000006	0.9999241	0.0000006	5.00E-06
robust	PD_mortality	eQTLGen	ENSG00000104859	CLASRP	5179	4.95E-15	6.52E-11	7.59E-05	0.9999235	0.0000006	0.9999241	0.0000006	5.00E-06
robust	PD_mortality	eQTLGen	ENSG00000130201	EXOC3L2	5429	7.83E-15	1.03E-10	7.59E-05	0.9999235	0.0000006	0.9999241	0.0000006	5.00E-06
robust	PD_mortality	eQTLGen	ENSG00000007047	MARK4	5388	1.97E-307	2.60E-303	7.59E-05	0.9999235	0.0000006	0.9999241	0.0000006	5.00E-06
robust	PD_mortality	eQTLGen	ENSG00000216588	IGSF23	5439	2.48E-26	3.26E-22	7.59E-05	0.9999235	0.0000005	0.9999241	0.0000005	5.00E-06

robust	PD_mortality	eQTLGen	ENSG00000125753	VASP	5638	9.18E-308	1.21E-303	7.59E-05	0.9999236	0.0000005	0.9999241	0.0000005	5.00E-06
robust	PD_mortality	eQTLGen	ENSG00000213889	PPM1N	5610	6.95E-96	9.16E-92	7.59E-05	0.9999236	0.0000005	0.9999241	0.0000005	5.00E-06
robust	PD_mortality	eQTLGen	ENSG00000267348		5238	1.24E-19	1.63E-15	7.59E-05	0.9999236	0.0000005	0.9999241	0.0000005	5.00E-06
robust	PD_mortality	eQTLGen	ENSG00000125740	FOSB	5629	1.69E-11	2.23E-07	7.59E-05	0.9999234	0.0000005	0.9999239	0.0000005	5.00E-06
robust	PD_mortality	eQTLGen	ENSG00000104884	ERCC2	5662	1.93E-90	2.55E-86	7.59E-05	0.9999237	0.0000005	0.9999241	0.0000005	5.00E-06

Supplementary Table 3. Full colocalisation results for PD H&Y3 and eQTLs in eQTLGen and PsychENCODE.

prior_type	GWAS_1	dataset	gene_2	hgnc_symbol	nsnps	PP.H0.abf	PP.H1.abf	PP.H2.abf	PP.H3.abf	PP.H4.abf	sum_PPH3_PPH4	ratio_PPH4_PPH3	p12
liberal	PD_HY3	psychencode	ENSG00000162927	PUS10	2632	1.42E-20	1.00E-20	0.1777668	0.1241484	0.6980847	0.8222332	5.6229851	1.00E-05
liberal	PD_HY3	psychencode	ENSG00000237522	NONOP2	2699	0.049621127	0.03488599	0.1831185	0.1281367	0.6042376	0.7323743	4.7155695	1.00E-05
robust	PD_HY3	psychencode	ENSG00000162927	PUS10	2632	2.19E-20	1.54E-20	0.2730851	0.1907166	0.5361983	0.7269149	2.8114926	5.00E-06
robust	PD_HY3	psychencode	ENSG00000237522	NONOP2	2699	0.071102543	0.049988437	0.2623921	0.1836082	0.4329087	0.6165169	2.3577847	5.00E-06
liberal	PD_HY3	eQTLGen	ENSG00000162924	REL	4328	3.95E-53	4.75E-52	0.0492792	0.5917167	0.3590041	0.9507208	0.6067162	1.00E-05
liberal	PD_HY3	psychencode	ENSG00000177133	LINC00982	2631	0.003742761	0.423467658	0.0019487	0.2201278	0.3507131	0.5708409	1.5932253	1.00E-05
liberal	PD_HY3	eQTLGen	ENSG00000162927	PUS10	4233	8.45E-181	1.02E-179	0.0584524	0.7020047	0.2395429	0.9415476	0.3412269	1.00E-05
robust	PD_HY3	eQTLGen	ENSG00000162924	REL	4328	4.82E-53	5.79E-52	0.0600601	0.7211678	0.2187721	0.9399399	0.3033581	5.00E-06
robust	PD_HY3	psychencode	ENSG00000177133	LINC00982	2631	0.004538642	0.513516074	0.0023630	0.2669369	0.2126453	0.4795822	0.7966126	5.00E-06
liberal	PD_HY3	psychencode	ENSG00000142611	PRDM16	2631	0.005837132	0.66043126	0.0010642	0.1201892	0.2124782	0.3326675	1.7678638	1.00E-05
liberal	PD_HY3	psychencode	ENSG00000248333	CDK11B	1239	0.006226631	0.722160576	0.0007126	0.0824601	0.1884400	0.2709002	2.2852260	1.00E-05
liberal	PD_HY3	psychencode	ENSG00000271921		1984	0.006649391	0.751942239	0.0008460	0.0955291	0.1450333	0.2405623	1.5182112	1.00E-05
robust	PD_HY3	eQTLGen	ENSG00000162927	PUS10	4233	9.60E-181	1.15E-179	0.0664059	0.7975255	0.1360686	0.9335941	0.1706135	5.00E-06
liberal	PD_HY3	psychencode	ENSG00000226286		2572	0.006579451	0.744077014	0.0011337	0.1280933	0.1201165	0.2482098	0.9377262	1.00E-05
robust	PD_HY3	psychencode	ENSG00000142611	PRDM16	2631	0.006530977	0.738935059	0.0011906	0.1344758	0.1188675	0.2533433	0.8839319	5.00E-06
liberal	PD_HY3	psychencode	ENSG00000272235		2761	0.006557616	0.740758833	0.0011889	0.1341838	0.1173109	0.2514946	0.8742553	1.00E-05
liberal	PD_HY3	psychencode	ENSG00000234396		1931	0.006951817	0.787601808	0.0008228	0.0931118	0.1115117	0.2046235	1.1976107	1.00E-05
liberal	PD_HY3	psychencode	ENSG00000269737		1241	0.007053903	0.818222442	0.0005451	0.0631167	0.1110619	0.1741786	1.7596275	1.00E-05
liberal	PD_HY3	psychencode	ENSG00000243558		1931	0.006988399	0.791746276	0.0008110	0.0917684	0.1086859	0.2004544	1.1843499	1.00E-05
liberal	PD_HY3	psychencode	ENSG00000223663		867	0.013445398	0.829160348	0.0008199	0.0504555	0.1061189	0.1565744	2.1032190	1.00E-05
robust	PD_HY3	psychencode	ENSG00000248333	CDK11B	1239	0.00687433	0.797280349	0.0007867	0.0910377	0.1040209	0.1950586	1.1426130	5.00E-06
liberal	PD_HY3	psychencode	ENSG00000269227		1241	0.007003476	0.812373131	0.0006745	0.0781403	0.1018086	0.1799489	1.3028948	1.00E-05
liberal	PD_HY3	psychencode	ENSG00000157933	SKI	1916	0.006672975	0.755302264	0.0011971	0.1353983	0.1014294	0.2368276	0.7491185	1.00E-05
liberal	PD_HY3	psychencode	ENSG00000242485	MRPL20	894	0.00734465	0.865677125	0.0003719	0.0437545	0.0828518	0.1266063	1.8935581	1.00E-05
robust	PD_HY3	psychencode	ENSG00000271921		1984	0.007169283	0.810733939	0.0009122	0.1029981	0.0781865	0.1811846	0.7591056	5.00E-06
liberal	PD_HY3	eQTLGen	ENSG00000138735	PDE5A	5052	2.99E-48	5.10E-48	0.3411935	0.5809776	0.0778290	0.6588065	0.1339621	1.00E-05
liberal	PD_HY3	psychencode	ENSG00000215014		1141	0.006905286	0.807369503	0.0009548	0.1115604	0.0732100	0.1847704	0.6562367	1.00E-05
liberal	PD_HY3	psychencode	ENSG00000228037		2142	0.006999961	0.79301215	0.0011194	0.1267470	0.0721215	0.1988685	0.5690191	1.00E-05
liberal	PD_HY3	psychencode	ENSG00000157916	RER1	2025	0.007153269	0.806891025	0.0010043	0.1132160	0.0717354	0.1849514	0.6336157	1.00E-05
liberal	PD_HY3	psychencode	ENSG00000272004		1241	0.007312145	0.848177354	0.0006285	0.0728364	0.0710456	0.1438820	0.9754131	1.00E-05

liberal	PD_HY3	psychencode	ENSG00000142609	C1orf222	1503	0.007277704	0.832659799	0.0007782	0.0889633	0.0703211	0.1592843	0.7904505	1.00E-05
liberal	PD_HY3	psychencode	ENSG00000225931		2128	0.007181404	0.812828381	0.0009675	0.1094377	0.0695850	0.1790227	0.6358415	1.00E-05
liberal	PD_HY3	psychencode	ENSG00000215791		1132	0.006925042	0.810228875	0.0009610	0.1123660	0.0695191	0.1818851	0.6186850	1.00E-05
liberal	PD_HY3	psychencode	ENSG00000008130	NADK	1267	0.007257216	0.84390117	0.0006822	0.0792552	0.0689043	0.1481595	0.8693981	1.00E-05
liberal	PD_HY3	psychencode	ENSG00000189409	MMP23B	1228	0.00717052	0.831947427	0.0007895	0.0915275	0.0685651	0.1600926	0.7491208	1.00E-05
liberal	PD_HY3	psychencode	ENSG00000205090	TMEM240	1113	0.007404333	0.867811428	0.0004793	0.0561106	0.0681943	0.1243049	1.2153567	1.00E-05
liberal	PD_HY3	psychencode	ENSG00000197530	MIB2	1207	0.007062427	0.821078679	0.0008902	0.1034256	0.0675431	0.1709687	0.6530592	1.00E-05
liberal	PD_HY3	psychencode	ENSG00000221978	CCNL2	877	0.007294414	0.812916173	0.0009993	0.1112974	0.0674927	0.1787901	0.6064180	1.00E-05
liberal	PD_HY3	psychencode	ENSG00000272455		894	0.007205403	0.849264857	0.0006417	0.0755689	0.0673191	0.1428880	0.8908313	1.00E-05
liberal	PD_HY3	psychencode	ENSG00000180535	BHLHA15	3403	7.54E-41	5.75E-41	0.5295751	0.4040879	0.0663370	0.4704249	0.1641648	1.00E-05
liberal	PD_HY3	psychencode	ENSG00000182873		1925	0.006934657	0.785988968	0.0012332	0.1397113	0.0661318	0.2058431	0.4733461	1.00E-05
liberal	PD_HY3	psychencode	ENSG00000179403	VWA1	940	0.007444541	0.875084355	0.0004359	0.0511725	0.0658627	0.1170352	1.2870737	1.00E-05
robust	PD_HY3	psychencode	ENSG00000226286		2572	0.00699985	0.791620337	0.0012062	0.1362780	0.0638957	0.2001737	0.4688631	5.00E-06
liberal	PD_HY3	psychencode	ENSG00000067606	PRKCZ	1719	0.007429271	0.845381999	0.0007277	0.0827373	0.0637238	0.1464611	0.7701941	1.00E-05
liberal	PD_HY3	psychencode	ENSG00000233542		1660	0.006626399	0.756823231	0.0015067	0.1720233	0.0630203	0.2350437	0.3663477	1.00E-05
liberal	PD_HY3	psychencode	ENSG00000269753		1950	0.0069466	0.786556233	0.0012602	0.1426277	0.0626093	0.2052370	0.4389700	1.00E-05
robust	PD_HY3	psychencode	ENSG00000272235		2761	0.006966223	0.786915718	0.0012630	0.1425448	0.0623103	0.2048551	0.4371277	5.00E-06
liberal	PD_HY3	psychencode	ENSG00000225285		940	0.007062591	0.830187183	0.0008500	0.0998566	0.0620436	0.1619002	0.6213271	1.00E-05
liberal	PD_HY3	psychencode	ENSG00000272106		1208	0.007189692	0.83579245	0.0008117	0.0942974	0.0619087	0.1562062	0.6565261	1.00E-05
liberal	PD_HY3	psychencode	ENSG00000078369	GNB1	1429	0.007284911	0.837699784	0.0008084	0.0928987	0.0613082	0.1542069	0.6599466	1.00E-05
liberal	PD_HY3	psychencode	ENSG00000169885	CALML6	1490	0.007431244	0.850558862	0.0007006	0.0801315	0.0611778	0.1413093	0.7634671	1.00E-05
liberal	PD_HY3	psychencode	ENSG00000160072	ATAD3B	1020	0.007249935	0.849366304	0.0007016	0.0821357	0.0605465	0.1426822	0.7371525	1.00E-05
liberal	PD_HY3	psychencode	ENSG00000228594	C1orf233	1183	0.007410877	0.863478265	0.0005855	0.0681560	0.0603694	0.1285254	0.8857536	1.00E-05
liberal	PD_HY3	psychencode	ENSG00000238164		2127	0.007055801	0.797980419	0.0011813	0.1335377	0.0602448	0.1937825	0.4511449	1.00E-05
liberal	PD_HY3	psychencode	ENSG00000264293	RN7SL657P	894	0.007419092	0.874451299	0.0004877	0.0574256	0.0602163	0.1176419	1.0485962	1.00E-05
liberal	PD_HY3	psychencode	ENSG00000187730	GABRD	1669	0.007208942	0.822942381	0.0009547	0.1089243	0.0599697	0.1688940	0.5505631	1.00E-05
liberal	PD_HY3	psychencode	ENSG00000249356		3469	0.000749827	0.000134994	0.7964416	0.1433271	0.0593465	0.2026736	0.4140634	1.00E-05
robust	PD_HY3	psychencode	ENSG00000234396		1931	0.007362309	0.834108227	0.0008714	0.0986099	0.0590481	0.1576580	0.5988054	5.00E-06
liberal	PD_HY3	psychencode	ENSG00000269554		1926	0.007257403	0.822588794	0.0009735	0.1102806	0.0588997	0.1691803	0.5340897	1.00E-05
robust	PD_HY3	psychencode	ENSG00000269737		1241	0.007468644	0.866330591	0.0005771	0.0668277	0.0587959	0.1256236	0.8798137	5.00E-06

liberal	PD_HY3	psychencode	ENSG00000178642		2006	0.007097254	0.801494451	0.0011647	0.1314661	0.0587776	0.1902436	0.4470933	1.00E-05
liberal	PD_HY3	psychencode	ENSG00000203301		1931	0.007211755	0.816975099	0.0010270	0.1162807	0.0585055	0.1747862	0.5031400	1.00E-05
liberal	PD_HY3	psychencode	ENSG00000138735	PDE5A	3595	0.215171845	0.046340023	0.5595780	0.1204539	0.0584563	0.1789102	0.4853004	1.00E-05
liberal	PD_HY3	psychencode	ENSG00000157873	TNFRSF14	2130	0.007098983	0.803156374	0.0011550	0.1306177	0.0579719	0.1885896	0.4438292	1.00E-05
liberal	PD_HY3	psychencode	ENSG00000272161		1947	0.007395656	0.837385505	0.0008536	0.0965923	0.0577729	0.1543652	0.5981114	1.00E-05
robust	PD_HY3	psychencode	ENSG00000243558		1931	0.007389993	0.837244633	0.0008576	0.0970420	0.0574658	0.1545078	0.5921750	5.00E-06
liberal	PD_HY3	psychencode	ENSG00000197921	HES5	2123	0.007158065	0.808960551	0.0011105	0.1254457	0.0573251	0.1827709	0.4569716	1.00E-05
liberal	PD_HY3	psychencode	ENSG00000271806		1839	0.007203308	0.816404517	0.0010469	0.1185957	0.0567496	0.1753453	0.4785127	1.00E-05
liberal	PD_HY3	psychencode	ENSG00000272449		2129	0.006864207	0.776007292	0.0014083	0.1591541	0.0565661	0.2157202	0.3554170	1.00E-05
liberal	PD_HY3	psychencode	ENSG00000238240		1958	0.006814118	0.771170516	0.0014522	0.1642876	0.0562756	0.2205632	0.3425434	1.00E-05
robust	PD_HY3	psychencode	ENSG00000223663		867	0.014198777	0.875620277	0.0008658	0.0532826	0.0560325	0.1093151	1.0516095	5.00E-06
liberal	PD_HY3	psychencode	ENSG00000256761		2631	0.007127531	0.806430984	0.0011500	0.1300546	0.0552369	0.1852915	0.4247207	1.00E-05
liberal	PD_HY3	psychencode	ENSG00000197785	ATAD3A	1068	0.006536566	0.767463572	0.0014427	0.1693376	0.0552195	0.2245571	0.3260912	1.00E-05
liberal	PD_HY3	psychencode	ENSG00000231050		1430	0.006251873	0.718909227	0.0018983	0.2182310	0.0547096	0.2729406	0.2506958	1.00E-05
liberal	PD_HY3	psychencode	ENSG00000189339	SLC35E2B	1241	0.006574797	0.762648249	0.0015113	0.1752544	0.0540112	0.2292656	0.3081873	1.00E-05
robust	PD_HY3	psychencode	ENSG00000269227		1241	0.007379104	0.855944371	0.0007107	0.0823313	0.0536345	0.1359658	0.6514474	5.00E-06
robust	PD_HY3	psychencode	ENSG00000157933	SKI	1916	0.007029473	0.795653579	0.0012611	0.1426318	0.0534241	0.1960559	0.3745592	5.00E-06
liberal	PD_HY3	psychencode	ENSG00000205116	TMEM88B	929	0.005504604	0.64716034	0.0024853	0.2921390	0.0527107	0.3448497	0.1804303	1.00E-05
liberal	PD_HY3	psychencode	ENSG00000250412	KLHL2P1	3349	0.001544109	0.0002874	0.7988303	0.1486331	0.0507052	0.1993382	0.3411433	1.00E-05
liberal	PD_HY3	eQTLGen	ENSG00000150961	SEC24D	4816	5.29E-10	7.45E-10	0.3946146	0.5560091	0.0493763	0.6053854	0.0888048	1.00E-05
liberal	PD_HY3	psychencode	ENSG00000272782		3373	2.66E-40	2.02E-40	0.5430740	0.4114928	0.0454332	0.4569260	0.1104108	1.00E-05
liberal	PD_HY3	psychencode	ENSG00000142606	MMEL1	2156	0.005825982	0.660394872	0.0025231	0.2859514	0.0453047	0.3312561	0.1584349	1.00E-05
robust	PD_HY3	psychencode	ENSG00000242485	MRPL20	894	0.007662057	0.903088355	0.0003880	0.0456454	0.0432161	0.0888616	0.9467791	5.00E-06
liberal	PD_HY3	eQTLGen	ENSG00000205356	TECPR1	4709	3.38E-305	2.92E-305	0.5137764	0.4438993	0.0423243	0.4862236	0.0953466	1.00E-05
robust	PD_HY3	eQTLGen	ENSG00000138735	PDE5A	5052	3.12E-48	5.31E-48	0.3550084	0.6045014	0.0404901	0.6449916	0.0669810	5.00E-06
liberal	PD_HY3	psychencode	ENSG00000213455		3088	0.322928933	0.237548884	0.2308169	0.1697516	0.0389537	0.2087053	0.2294746	1.00E-05
liberal	PD_HY3	psychencode	ENSG00000237058		2207	0.004714864	0.534720072	0.0036871	0.4181168	0.0387612	0.4568780	0.0927043	1.00E-05
robust	PD_HY3	psychencode	ENSG00000215014		1141	0.007167658	0.838046196	0.0009911	0.1157992	0.0379959	0.1537951	0.3281183	5.00E-06
liberal	PD_HY3	eQTLGen	ENSG00000115421	PAPOLG	4389	0.056195862	0.675793875	0.0176692	0.2124467	0.0378943	0.2503410	0.1783710	1.00E-05
robust	PD_HY3	psychencode	ENSG00000228037		2142	0.007261828	0.822678538	0.0011613	0.1314886	0.0374098	0.1688983	0.2845096	5.00E-06
robust	PD_HY3	psychencode	ENSG00000157916	RER1	2025	0.007419386	0.836909033	0.0010417	0.1174278	0.0372021	0.1546299	0.3168078	5.00E-06
liberal	PD_HY3	psychencode	ENSG00000157881	PANK4	2123	0.004419965	0.499517265	0.0040256	0.4549072	0.0371300	0.4920372	0.0816210	1.00E-05
robust	PD_HY3	psychencode	ENSG00000272004		1241	0.007581459	0.879416685	0.0006517	0.0755190	0.0368311	0.1123502	0.4877065	5.00E-06
robust	PD_HY3	psychencode	ENSG00000142609	C1orf222	1503	0.007542917	0.863003454	0.0008065	0.0922052	0.0364418	0.1286471	0.3952252	5.00E-06

robust	PD_HY3	psychencode	ENSG00000225931		2128	0.007440269	0.842128135	0.0010024	0.1133825	0.0360467	0.1494292	0.3179208	5.00E-06
robust	PD_HY3	psychencode	ENSG00000215791		1132	0.007174422	0.839406274	0.0009956	0.1164124	0.0360113	0.1524237	0.3093425	5.00E-06
liberal	PD_HY3	psychencode	ENSG00000229393		2081	0.004195267	0.473772918	0.0042685	0.4820098	0.0357535	0.5177633	0.0741759	1.00E-05
robust	PD_HY3	psychencode	ENSG00000008130	NADK	1267	0.007516164	0.874012785	0.0007065	0.0820831	0.0356814	0.1177646	0.4346990	5.00E-06
liberal	PD_HY3	psychencode	ENSG00000173163	COMMD1	2567	0.502220831	0.324641065	0.0835939	0.0540005	0.0355437	0.0895442	0.6582113	1.00E-05
robust	PD_HY3	psychencode	ENSG00000189409	MMP23B	1228	0.007425071	0.861481212	0.0008175	0.0947766	0.0354996	0.1302762	0.3745604	5.00E-06
liberal	PD_HY3	psychencode	ENSG00000221048		3313	0.170095421	0.129272601	0.3779660	0.2872189	0.0354470	0.3226660	0.1234147	1.00E-05
robust	PD_HY3	psychencode	ENSG00000205090	TMEM240	1113	0.007665712	0.898445894	0.0004962	0.0580913	0.0353008	0.0933921	0.6076784	5.00E-06
liberal	PD_HY3	psychencode	ENSG00000164715	LMTK2	3317	0.451905772	0.342449101	0.0970001	0.0734704	0.0351746	0.1086450	0.4787580	1.00E-05
liberal	PD_HY3	psychencode	ENSG00000227284	MARK2P10	2726	0.499684596	0.324949923	0.0849974	0.0552396	0.0351285	0.0903681	0.6359304	1.00E-05
robust	PD_HY3	psychencode	ENSG00000197530	MIB2	1207	0.007309272	0.84977695	0.0009213	0.1070406	0.0349519	0.1419925	0.3265296	5.00E-06
robust	PD_HY3	psychencode	ENSG00000221978	CCNL2	877	0.007549171	0.841307235	0.0010342	0.1151844	0.0349250	0.1501094	0.3032090	5.00E-06
robust	PD_HY3	psychencode	ENSG00000272455		894	0.007456382	0.878846448	0.0006641	0.0782011	0.0348320	0.1130331	0.4454157	5.00E-06
robust	PD_HY3	psychencode	ENSG00000180535	BHLHA15	3403	7.80E-41	5.95E-41	0.5477429	0.4179507	0.0343064	0.4522571	0.0820824	5.00E-06
robust	PD_HY3	psychencode	ENSG00000182873		1925	0.007171799	0.812867159	0.0012754	0.1444890	0.0341967	0.1786856	0.2366731	5.00E-06
robust	PD_HY3	psychencode	ENSG00000179403	VWA1	940	0.007698048	0.904883405	0.0004507	0.0529150	0.0340528	0.0869678	0.6435369	5.00E-06
liberal	PD_HY3	psychencode	ENSG00000271146		2575	0.391008241	0.268717521	0.1820402	0.1250726	0.0331614	0.1582340	0.2651371	1.00E-05
robust	PD_HY3	psychencode	ENSG00000067606	PRKCZ	1719	0.007673772	0.873203924	0.0007516	0.0854602	0.0329105	0.1183707	0.3850970	5.00E-06
liberal	PD_HY3	psychencode	ENSG00000006453	BAIAP2L1	3133	0.386703882	0.285112227	0.1701346	0.1254056	0.0326437	0.1580493	0.2603048	1.00E-05
robust	PD_HY3	psychencode	ENSG00000233542		1660	0.006841991	0.781446752	0.0015557	0.1776202	0.0325354	0.2101555	0.1831738	5.00E-06
robust	PD_HY3	psychencode	ENSG00000269753		1950	0.007171089	0.811974812	0.0013009	0.1472369	0.0323163	0.1795532	0.2194850	5.00E-06
liberal	PD_HY3	psychencode	ENSG00000231859		3310	0.426140758	0.323871325	0.1238222	0.0940740	0.0320917	0.1261657	0.3411323	1.00E-05
robust	PD_HY3	psychencode	ENSG00000225285		940	0.007288699	0.856765596	0.0008772	0.1030535	0.0320150	0.1350685	0.3106635	5.00E-06
robust	PD_HY3	psychencode	ENSG00000272106		1208	0.007419353	0.862490286	0.0008376	0.0973096	0.0319431	0.1292527	0.3282630	5.00E-06
liberal	PD_HY3	psychencode	ENSG00000215914	MMP23A	1242	0.003660872	0.424769337	0.0046124	0.5351452	0.0318122	0.5669574	0.0594459	1.00E-05
robust	PD_HY3	psychencode	ENSG00000078369	GNB1	1429	0.007515285	0.864190768	0.0008340	0.0958365	0.0316235	0.1274600	0.3299733	5.00E-06
liberal	PD_HY3	psychencode	ENSG00000145390	USP53	3410	1.06E-23	1.89E-24	0.8217532	0.1466521	0.0315947	0.1782468	0.2154398	1.00E-05
robust	PD_HY3	psychencode	ENSG00000169885	CALML6	1490	0.00766573	0.877397469	0.0007227	0.0826600	0.0315541	0.1142141	0.3817335	5.00E-06
liberal	PD_HY3	psychencode	ENSG00000232713		2698	0.317983401	0.223542448	0.2508103	0.1762884	0.0313754	0.2076638	0.1779780	1.00E-05
robust	PD_HY3	psychencode	ENSG00000160072	ATAD3B	1020	0.007476265	0.875882102	0.0007235	0.0846998	0.0312183	0.1159181	0.3685762	5.00E-06
robust	PD_HY3	psychencode	ENSG00000228594	C1orf233	1183	0.007641535	0.890353314	0.0006037	0.0702773	0.0311242	0.1014015	0.4428768	5.00E-06
liberal	PD_HY3	psychencode	ENSG00000224475		3050	0.468610946	0.343068496	0.0908066	0.0664481	0.0310658	0.0975139	0.4675195	1.00E-05
robust	PD_HY3	psychencode	ENSG00000238164		2127	0.00727494	0.822764062	0.0012180	0.1376851	0.0310580	0.1687430	0.2255725	5.00E-06
robust	PD_HY3	psychencode	ENSG00000264293	RN7SL657P	894	0.007649402	0.901596696	0.0005029	0.0592083	0.0310428	0.0902510	0.5242981	5.00E-06

robust	PD_HY3	psychencode	ENSG00000187730	GABRD	1669	0.007431783	0.848380953	0.0009842	0.1122913	0.0309117	0.1432031	0.2752815	5.00E-06
liberal	PD_HY3	psychencode	ENSG00000227361	RPS24P7	2621	0.445815589	0.294146851	0.1380924	0.0910818	0.0308633	0.1219451	0.3388527	1.00E-05
liberal	PD_HY3	psychencode	ENSG00000166448	TMEM130	2492	0.447731431	0.324647731	0.1141532	0.0827412	0.0307265	0.1134676	0.3713564	1.00E-05
liberal	PD_HY3	psychencode	ENSG00000231764	DLX6-AS1	2867	0.466411613	0.316508081	0.1111052	0.0753657	0.0306094	0.1059751	0.4061450	1.00E-05
robust	PD_HY3	psychencode	ENSG00000249356		3469	0.000772758	0.000139123	0.8207973	0.1477101	0.0305807	0.1782908	0.2070317	5.00E-06
liberal	PD_HY3	psychencode	ENSG00000196636	ACN9	2953	0.468810266	0.334933066	0.0967034	0.0690575	0.0304957	0.0995532	0.4415987	1.00E-05
robust	PD_HY3	psychencode	ENSG00000269554		1926	0.007477618	0.847548996	0.0010030	0.1136269	0.0303435	0.1439704	0.2670449	5.00E-06
liberal	PD_HY3	psychencode	ENSG00000164096	C4orf3	3353	0.406512588	0.075060194	0.4120391	0.0760503	0.0303378	0.1063881	0.3989176	1.00E-05
robust	PD_HY3	psychencode	ENSG00000178642		2006	0.007312149	0.825762618	0.0011999	0.1354467	0.0302786	0.1657253	0.2235466	5.00E-06
robust	PD_HY3	psychencode	ENSG00000203301		1931	0.007429076	0.841594026	0.0010579	0.1197847	0.0301342	0.1499190	0.2515700	5.00E-06
robust	PD_HY3	psychencode	ENSG00000138735	PDE5A	3595	0.221650273	0.047735236	0.5764258	0.1240805	0.0301082	0.1541887	0.2426502	5.00E-06
liberal	PD_HY3	psychencode	ENSG00000226046		3181	0.462791986	0.345690993	0.0924223	0.0690064	0.0300883	0.0990947	0.4360212	1.00E-05
liberal	PD_HY3	psychencode	ENSG00000106236	NPTX2	2931	0.451611832	0.331074811	0.1080492	0.0791804	0.0300837	0.1092641	0.3799394	1.00E-05
liberal	PD_HY3	psychencode	ENSG00000271707	ATP1B3P1	2615	0.505119088	0.343972395	0.0720152	0.0490105	0.0298827	0.0788933	0.6097207	1.00E-05
liberal	PD_HY3	psychencode	ENSG00000214389	RPS3AP26	3182	0.47313682	0.34966441	0.0847297	0.0625883	0.0298807	0.0924690	0.4774169	1.00E-05
robust	PD_HY3	psychencode	ENSG00000157873	TNFRSF14	2130	0.007310896	0.827131586	0.0011895	0.1345168	0.0298512	0.1643680	0.2219146	5.00E-06
liberal	PD_HY3	psychencode	ENSG00000226744		3381	0.442388589	0.335438984	0.1094336	0.0829477	0.0297911	0.1127388	0.3591559	1.00E-05
liberal	PD_HY3	psychencode	ENSG00000244318	RN7SL252P	2742	0.491993016	0.318209584	0.0972037	0.0628393	0.0297543	0.0925937	0.4734986	1.00E-05
robust	PD_HY3	psychencode	ENSG00000272161		1947	0.007615645	0.862294142	0.0008790	0.0994655	0.0297457	0.1292112	0.2990557	5.00E-06
robust	PD_HY3	psychencode	ENSG00000197921	HES5	2123	0.007369287	0.832831647	0.0011433	0.1291474	0.0295084	0.1586558	0.2284858	5.00E-06
liberal	PD_HY3	psychencode	ENSG00000271956	DLX6-AS2	2854	0.455376679	0.299603647	0.1300183	0.0855128	0.0294885	0.1150013	0.3448434	1.00E-05
liberal	PD_HY3	psychencode	ENSG00000199475	RN7SKP104	3111	0.453595462	0.335851977	0.1040918	0.0770424	0.0294184	0.1064608	0.3818470	1.00E-05
robust	PD_HY3	psychencode	ENSG00000271806		1839	0.007413669	0.840246321	0.0010775	0.1220591	0.0292034	0.1512625	0.2392563	5.00E-06
liberal	PD_HY3	psychencode	ENSG00000232032		3310	0.445892757	0.339135584	0.1055671	0.0802627	0.0291418	0.1094046	0.3630805	1.00E-05
robust	PD_HY3	psychencode	ENSG00000272449		2129	0.007063999	0.798593955	0.0014493	0.1637865	0.0291063	0.1928927	0.1777085	5.00E-06
liberal	PD_HY3	psychencode	ENSG00000231812		3359	0.431258199	0.327099043	0.1209200	0.0916859	0.0290369	0.1207228	0.3166996	1.00E-05
liberal	PD_HY3	psychencode	ENSG00000006377	DLX6	2848	0.504250628	0.326130482	0.0853991	0.0552039	0.0290158	0.0842198	0.5256117	1.00E-05
robust	PD_HY3	psychencode	ENSG00000238240		1958	0.007011404	0.793497809	0.0014942	0.1690441	0.0289525	0.1979966	0.1712717	5.00E-06
liberal	PD_HY3	psychencode	ENSG00000237818	RPS3AP29	3327	0.380434413	0.289044384	0.1713867	0.1301863	0.0289482	0.1591345	0.2223599	1.00E-05
liberal	PD_HY3	psychencode	ENSG00000228414		2707	0.459622557	0.321471107	0.1117994	0.0781663	0.0289407	0.1071069	0.3702452	1.00E-05
liberal	PD_HY3	psychencode	ENSG00000224551	HMGB3P21	2980	0.459819506	0.330775373	0.1049826	0.0754913	0.0289312	0.1044225	0.3832394	1.00E-05
liberal	PD_HY3	psychencode	ENSG00000263819	RN7SL478P	3326	0.469189783	0.356116777	0.0828863	0.0628821	0.0289251	0.0918071	0.4599891	1.00E-05
liberal	PD_HY3	psychencode	ENSG00000232097		3287	0.449100131	0.33993667	0.1038317	0.0785646	0.0285668	0.1071315	0.3636093	1.00E-05
liberal	PD_HY3	psychencode	ENSG00000205356	TECPR1	3395	0.432935847	0.329008607	0.1190907	0.0904742	0.0284907	0.1189649	0.3149042	1.00E-05

robust	PD_HY3	psychencode	ENSG00000256761		2631	0.007329973	0.829335955	0.0011826	0.1337486	0.0284029	0.1621515	0.2123603	5.00E-06
robust	PD_HY3	psychencode	ENSG00000197785	ATAD3A	1068	0.006722163	0.789254702	0.0014837	0.1741457	0.0283937	0.2025394	0.1630456	5.00E-06
liberal	PD_HY3	psychencode	ENSG00000105880	DLX5	2877	0.475627696	0.330926145	0.0973972	0.0677375	0.0283115	0.0960490	0.4179595	1.00E-05
robust	PD_HY3	psychencode	ENSG00000231050		1430	0.006427702	0.739127923	0.0019517	0.2243686	0.0281241	0.2524927	0.1253479	5.00E-06
liberal	PD_HY3	psychencode	ENSG00000164713	BRI3	3396	0.25138901	0.191057488	0.3009808	0.2287198	0.0278529	0.2565727	0.1217775	1.00E-05
liberal	PD_HY3	psychencode	ENSG00000135175	OCM2	3318	0.400656616	0.304007374	0.1521129	0.1153913	0.0278318	0.1432231	0.2411954	1.00E-05
robust	PD_HY3	psychencode	ENSG00000189339	SLC35E2B	1241	0.006757282	0.783815657	0.0015533	0.1801186	0.0277551	0.2078738	0.1540936	5.00E-06
liberal	PD_HY3	psychencode	ENSG00000237024		2687	0.481163342	0.336461505	0.0910336	0.0636290	0.0277126	0.0913416	0.4355335	1.00E-05
liberal	PD_HY3	psychencode	ENSG00000221245		3465	0.612765788	0.129464789	0.1902412	0.0401667	0.0273615	0.0675282	0.6811999	1.00E-05
robust	PD_HY3	psychencode	ENSG00000205116	TMEM88B	929	0.005653607	0.664678179	0.0025526	0.3000468	0.0270688	0.3271156	0.0902152	5.00E-06
liberal	PD_HY3	psychencode	ENSG00000230702		2576	0.504729439	0.327054073	0.0858223	0.0555842	0.0268099	0.0823942	0.4823300	1.00E-05
liberal	PD_HY3	psychencode	ENSG00000225905		913	0.003224886	0.379720286	0.0049719	0.5854049	0.0266779	0.6120829	0.0455718	1.00E-05
liberal	PD_HY3	psychencode	ENSG00000272950		3304	1.14E-24	8.59E-25	0.5554668	0.4180042	0.0265290	0.4445332	0.0634660	1.00E-05
liberal	PD_HY3	psychencode	ENSG00000235982		3363	0.38405452	0.291313996	0.1696238	0.1286371	0.0263705	0.1550076	0.2049991	1.00E-05
robust	PD_HY3	psychencode	ENSG00000250412	KLHL2P1	3349	0.001584275	0.000294876	0.8196095	0.1524993	0.0260121	0.1785114	0.1705716	5.00E-06
liberal	PD_HY3	eQTLGen	ENSG00000196636	ACN9	4165	3.00E-35	2.39E-35	0.5416952	0.4322982	0.0260066	0.4583048	0.0601589	1.00E-05
liberal	PD_HY3	psychencode	ENSG00000242158	RN7SL361P	2616	0.421205836	0.285208012	0.1595637	0.1080182	0.0260043	0.1340225	0.2407399	1.00E-05
liberal	PD_HY3	psychencode	ENSG00000236498		2574	0.509788315	0.329332486	0.0821355	0.0530353	0.0257085	0.0787437	0.4847424	1.00E-05
liberal	PD_HY3	eQTLGen	ENSG00000180535	BHLHA15	4709	0.330875262	0.286210466	0.1916449	0.1657493	0.0255201	0.1912694	0.1539684	1.00E-05
liberal	PD_HY3	psychencode	ENSG00000212978		2583	1.50E-20	1.03E-20	0.5772510	0.3973490	0.0254001	0.4227490	0.0639239	1.00E-05
robust	PD_HY3	eQTLGen	ENSG00000150961	SEC24D	4816	5.42E-10	7.64E-10	0.4046035	0.5700834	0.0253131	0.5953965	0.0444024	5.00E-06
liberal	PD_HY3	psychencode	ENSG00000115421	PAPOLG	2665	0.467831857	0.325330256	0.1071305	0.0744733	0.0252340	0.0997073	0.3388327	1.00E-05
liberal	PD_HY3	psychencode	ENSG00000162924	REL	2707	0.493155075	0.344924559	0.0804633	0.0562528	0.0252043	0.0814571	0.4480545	1.00E-05
liberal	PD_HY3	eQTLGen	ENSG00000214389	RPS3AP26	4439	0.419233609	0.348765992	0.1129246	0.0939183	0.0251575	0.1190758	0.2678658	1.00E-05
liberal	PD_HY3	psychencode	ENSG00000234624		2617	0.503669002	0.347204732	0.0733988	0.0505724	0.0251550	0.0757274	0.4974056	1.00E-05
liberal	PD_HY3	psychencode	ENSG00000229839		2442	0.510167805	0.259474595	0.1360948	0.0691936	0.0250691	0.0942628	0.3623038	1.00E-05
liberal	PD_HY3	psychencode	ENSG00000271134		2615	0.449036716	0.303404494	0.1331010	0.0899089	0.0245489	0.1144578	0.2730414	1.00E-05
liberal	PD_HY3	psychencode	ENSG00000178636		3410	0.409472202	0.073091163	0.4183283	0.0746475	0.0244608	0.0991083	0.3276844	1.00E-05
liberal	PD_HY3	psychencode	ENSG00000229831		2611	0.50510388	0.330949892	0.0844773	0.0553263	0.0241426	0.0794690	0.4363674	1.00E-05
liberal	PD_HY3	eQTLGen	ENSG00000142611	PRDM16	4240	0.00761516	0.871317011	0.0008409	0.0961869	0.0240400	0.1202270	0.2499303	1.00E-05
liberal	PD_HY3	eQTLGen	ENSG00000115464	USP34	3993	7.04E-64	8.39E-63	0.0755837	0.9005429	0.0238734	0.9244163	0.0265100	1.00E-05
liberal	PD_HY3	psychencode	ENSG00000115484	CCT4	2568	0.47859472	0.30943905	0.1143032	0.0738798	0.0237831	0.0976630	0.3219166	1.00E-05
liberal	PD_HY3	psychencode	ENSG00000270820		2664	0.419801643	0.290840663	0.1570443	0.1087775	0.0235359	0.1323134	0.2163671	1.00E-05
liberal	PD_HY3	eQTLGen	ENSG00000164715	LMTK2	4603	2.03E-182	1.74E-182	0.5259115	0.4506665	0.0234220	0.4740885	0.0519719	1.00E-05

liberal	PD_HY3	psychencode	ENSG00000271243		2769	0.498530355	0.347573822	0.0769060	0.0535952	0.0233946	0.0769898	0.4365049	1.00E-05
liberal	PD_HY3	psychencode	ENSG00000213495		2595	0.469851865	0.317580277	0.1129341	0.0763106	0.0233232	0.0996338	0.3056346	1.00E-05
robust	PD_HY3	psychencode	ENSG00000272782		3373	2.72E-40	2.06E-40	0.5556975	0.4210578	0.0232447	0.4443025	0.0552054	5.00E-06
liberal	PD_HY3	psychencode	ENSG00000172399	MYOZ2	3455	1.64E-07	2.95E-08	0.8281653	0.1486291	0.0232055	0.1718345	0.1561301	1.00E-05
robust	PD_HY3	psychencode	ENSG00000142606	MMEL1	2156	0.005961012	0.675701086	0.0025815	0.2925790	0.0231774	0.3157564	0.0792175	5.00E-06
liberal	PD_HY3	psychencode	ENSG00000115464	USP34	2581	0.49483279	0.34012372	0.0841648	0.0578277	0.0230511	0.0808787	0.3986166	1.00E-05
liberal	PD_HY3	psychencode	ENSG00000238228	OR7E7P	3295	0.236848019	0.179465111	0.3190457	0.2417252	0.0229159	0.2646412	0.0948016	1.00E-05
liberal	PD_HY3	psychencode	ENSG00000249244		3404	1.99E-97	3.92E-98	0.8167958	0.1604180	0.0227862	0.1832042	0.1420429	1.00E-05
liberal	PD_HY3	psychencode	ENSG00000119866	BCL11A	2643	0.49667515	0.333791065	0.0878018	0.0589846	0.0227474	0.0817320	0.3856506	1.00E-05
liberal	PD_HY3	eQTLGen	ENSG00000145390	USP53	4858	1.40E-304	2.15E-304	0.3852846	0.5920624	0.0226530	0.6147154	0.0382612	1.00E-05
liberal	PD_HY3	eQTLGen	ENSG00000006453	BAIAP2L1	4559	6.93E-84	5.88E-84	0.5286774	0.4486821	0.0226404	0.4713226	0.0504599	1.00E-05
liberal	PD_HY3	eQTLGen	ENSG00000238228	OR7E7P	4477	0.181416474	0.153823087	0.3475691	0.2946814	0.0225099	0.3171913	0.0763873	1.00E-05
liberal	PD_HY3	psychencode	ENSG00000213486		2733	0.485978033	0.337564417	0.0908734	0.0630990	0.0224851	0.0855841	0.3563472	1.00E-05
liberal	PD_HY3	psychencode	ENSG00000241524	RN7SL632P	2687	0.483093999	0.337403965	0.0926071	0.0646567	0.0222382	0.0868949	0.3439421	1.00E-05
robust	PD_HY3	eQTLGen	ENSG00000205356	TECPR1	4709	3.45E-305	2.98E-305	0.5248841	0.4534962	0.0216197	0.4751159	0.0476733	5.00E-06
liberal	PD_HY3	psychencode	ENSG00000250950		3370	1.89E-17	3.78E-18	0.8157302	0.1627310	0.0215388	0.1842698	0.1323583	1.00E-05
liberal	PD_HY3	eQTLGen	ENSG00000164713	BRI3	4686	2.14E-117	1.84E-117	0.5258774	0.4526811	0.0214415	0.4741226	0.0473656	1.00E-05
liberal	PD_HY3	psychencode	ENSG00000245958		3369	5.93E-216	1.18E-216	0.8158924	0.1627497	0.0213579	0.1841076	0.1312316	1.00E-05
liberal	PD_HY3	eQTLGen	ENSG00000164109	MAD2L1	5638	7.55E-29	1.28E-28	0.3626645	0.6160094	0.0213261	0.6373355	0.0346197	1.00E-05
liberal	PD_HY3	psychencode	ENSG00000242735		2443	0.465517018	0.236774465	0.1834337	0.0932783	0.0209965	0.1142748	0.2250953	1.00E-05
liberal	PD_HY3	psychencode	ENSG00000170264	FAM161A	2580	0.062022442	0.040198849	0.5324739	0.3450942	0.0202106	0.3653048	0.0585654	1.00E-05
robust	PD_HY3	psychencode	ENSG00000213455		3088	0.329343505	0.24226749	0.2354018	0.1731235	0.0198637	0.1929872	0.1147373	5.00E-06
robust	PD_HY3	psychencode	ENSG00000237058		2207	0.004808047	0.545288092	0.0037599	0.4263803	0.0197636	0.4461439	0.0463522	5.00E-06
liberal	PD_HY3	eQTLGen	ENSG00000106236	NPTX2	4176	5.66E-12	4.70E-12	0.5352669	0.4452157	0.0195173	0.4647331	0.0438379	1.00E-05
liberal	PD_HY3	psychencode	ENSG00000273302		2588	0.036866004	0.0253636	0.5440502	0.3742840	0.0194363	0.3937202	0.0519292	1.00E-05
robust	PD_HY3	eQTLGen	ENSG00000115421	PAPOLG	4389	0.057281178	0.688845548	0.0180105	0.2165497	0.0193131	0.2358628	0.0891855	5.00E-06
liberal	PD_HY3	eQTLGen	ENSG00000260091		4848	7.14E-300	2.53E-300	0.7246278	0.2562808	0.0190914	0.2753722	0.0744940	1.00E-05
robust	PD_HY3	psychencode	ENSG00000157881	PANK4	2123	0.004503573	0.508966224	0.0041017	0.4635123	0.0189162	0.4824285	0.0408105	5.00E-06
liberal	PD_HY3	eQTLGen	ENSG00000197530	MIB2	3046	0.006377989	0.778251278	0.0015973	0.1948865	0.0188870	0.2137734	0.0969127	1.00E-05
liberal	PD_HY3	psychencode	ENSG00000186889	TMEM17	2427	3.29E-54	5.74E-55	0.8357276	0.1458642	0.0184082	0.1642724	0.1262008	1.00E-05
robust	PD_HY3	psychencode	ENSG00000229393		2081	0.00427163	0.482396608	0.0043462	0.4907834	0.0182022	0.5089855	0.0370880	5.00E-06
liberal	PD_HY3	psychencode	ENSG00000227775		1248	0.001696952	0.197127055	0.0066830	0.7763113	0.0181817	0.7944930	0.0234207	1.00E-05
liberal	PD_HY3	psychencode	ENSG00000082898	XPO1	2744	9.23E-08	6.43E-08	0.5787083	0.4031170	0.0181745	0.4212915	0.0450850	1.00E-05
robust	PD_HY3	psychencode	ENSG00000173163	COMMD1	2567	0.51130772	0.33051493	0.0851064	0.0549775	0.0180934	0.0730709	0.3291056	5.00E-06

robust	PD_HY3	psychencode	ENSG00000221048		3313	0.173164506	0.131605107	0.3847858	0.2924013	0.0180433	0.3104446	0.0617074	5.00E-06
robust	PD_HY3	psychencode	ENSG00000164715	LMTK2	3317	0.459995847	0.348579669	0.0987366	0.0747857	0.0179021	0.0926878	0.2393790	5.00E-06
robust	PD_HY3	psychencode	ENSG00000227284	MARK2P10	2726	0.508618093	0.330759467	0.0865170	0.0562271	0.0178783	0.0741054	0.3179652	5.00E-06
liberal	PD_HY3	eQTLGen	ENSG00000250412	KLHL2P1	4849	0.089368772	0.142487475	0.2893669	0.4613424	0.0174344	0.4787768	0.0377905	1.00E-05
liberal	PD_HY3	psychencode	ENSG00000164109	MAD2L1	4018	0.008046038	0.001824568	0.7929448	0.1797956	0.0173891	0.1971846	0.0967157	1.00E-05
liberal	PD_HY3	eQTLGen	ENSG00000248280		4844	4.39E-300	2.21E-300	0.6542228	0.3284114	0.0173658	0.3457772	0.0528781	1.00E-05
robust	PD_HY3	psychencode	ENSG00000271146		2575	0.397600737	0.273248165	0.1851095	0.1271814	0.0168602	0.1440416	0.1325685	5.00E-06
liberal	PD_HY3	psychencode	ENSG00000150961	SEC24D	3355	0.263331725	0.040336748	0.5893614	0.0902608	0.0167094	0.1069701	0.1851231	1.00E-05
robust	PD_HY3	psychencode	ENSG00000006453	BAIAP2L1	3133	0.393120328	0.289842997	0.1729576	0.1274864	0.0165927	0.1440791	0.1301524	5.00E-06
liberal	PD_HY3	eQTLGen	ENSG00000178636		4918	5.87E-50	1.60E-50	0.7731919	0.2104604	0.0163477	0.2268081	0.0776761	1.00E-05
robust	PD_HY3	psychencode	ENSG00000231859		3310	0.433090054	0.329152861	0.1258414	0.0956081	0.0163075	0.1119157	0.1705661	5.00E-06
robust	PD_HY3	psychencode	ENSG00000215914	MMP23A	1242	0.003720043	0.431634959	0.0046870	0.5437949	0.0161632	0.5599580	0.0297229	5.00E-06
robust	PD_HY3	psychencode	ENSG00000145390	USP53	3410	1.08E-23	1.92E-24	0.8349431	0.1490060	0.0160509	0.1650569	0.1077199	5.00E-06
robust	PD_HY3	psychencode	ENSG00000232713		2698	0.323051341	0.227105212	0.2548077	0.1790980	0.0159378	0.1950358	0.0889890	5.00E-06
robust	PD_HY3	psychencode	ENSG00000224475		3050	0.476004679	0.348481423	0.0922394	0.0674966	0.0157780	0.0832745	0.2337597	5.00E-06
liberal	PD_HY3	psychencode	ENSG00000243554		3310	0.027254386	0.020672015	0.5324543	0.4038422	0.0157771	0.4196193	0.0390674	1.00E-05
robust	PD_HY3	psychencode	ENSG00000227361	RPS24P7	2621	0.452803091	0.298757169	0.1402568	0.0925094	0.0156735	0.1081829	0.1694263	5.00E-06
robust	PD_HY3	psychencode	ENSG00000166448	TMEM130	2492	0.454717359	0.329713191	0.1159343	0.0840322	0.0156029	0.0996351	0.1856782	5.00E-06
robust	PD_HY3	psychencode	ENSG00000231764	DLX6-AS1	2867	0.473660848	0.32142743	0.1128321	0.0765371	0.0155426	0.0920796	0.2030725	5.00E-06
liberal	PD_HY3	psychencode	ENSG00000145388	METTL14	3385	0.081146522	0.0117134	0.7791760	0.1124576	0.0155066	0.1279641	0.1378880	1.00E-05
robust	PD_HY3	psychencode	ENSG00000196636	ACN9	2953	0.476069304	0.340119154	0.0982008	0.0701268	0.0154840	0.0856108	0.2207994	5.00E-06
robust	PD_HY3	psychencode	ENSG00000164096	C4orf3	3353	0.412773915	0.076216312	0.4183856	0.0772217	0.0154025	0.0926242	0.1994588	5.00E-06
liberal	PD_HY3	psychencode	ENSG00000162928	PEX13	2632	5.51E-18	3.87E-18	0.5783876	0.4062202	0.0153922	0.4216124	0.0378912	1.00E-05
robust	PD_HY3	psychencode	ENSG00000226046		3181	0.469860634	0.35097105	0.0938339	0.0700604	0.0152739	0.0853344	0.2180106	5.00E-06
robust	PD_HY3	psychencode	ENSG00000106236	NPTX2	2931	0.458508661	0.336130848	0.1096993	0.0803896	0.0152716	0.0956612	0.1899697	5.00E-06
robust	PD_HY3	psychencode	ENSG00000271707	ATP1B3P1	2615	0.512780735	0.349189769	0.0731076	0.0497539	0.0151680	0.0649219	0.3048604	5.00E-06
robust	PD_HY3	psychencode	ENSG00000214389	RPS3AP26	3182	0.480312867	0.354967755	0.0860148	0.0635376	0.0151670	0.0787045	0.2387085	5.00E-06
robust	PD_HY3	psychencode	ENSG00000226744		3381	0.449077862	0.340511093	0.1110883	0.0842019	0.0151208	0.0993227	0.1795779	5.00E-06
liberal	PD_HY3	psychencode	ENSG00000183444	OR7E38P	3306	0.004779763	0.003624445	0.5553692	0.4211156	0.0151110	0.4362266	0.0358833	1.00E-05
robust	PD_HY3	psychencode	ENSG00000244318	RN7SL252P	2742	0.499423017	0.323015135	0.0986717	0.0637883	0.0151018	0.0788902	0.2367493	5.00E-06
robust	PD_HY3	psychencode	ENSG00000271956	DLX6-AS2	2854	0.462191351	0.304087188	0.1319640	0.0867925	0.0149649	0.1017574	0.1724217	5.00E-06
robust	PD_HY3	psychencode	ENSG00000199475	RN7SKP104	3111	0.460367096	0.340865842	0.1056457	0.0781925	0.0149288	0.0931213	0.1909235	5.00E-06
robust	PD_HY3	psychencode	ENSG00000232032		3310	0.45248589	0.344150166	0.1071281	0.0814495	0.0147864	0.0962359	0.1815402	5.00E-06
robust	PD_HY3	psychencode	ENSG00000231812		3359	0.437611638	0.331917975	0.1227014	0.0930366	0.0147323	0.1077690	0.1583498	5.00E-06

robust	PD_HY3	psychencode	ENSG00000006377	DLX6	2848	0.511673953	0.330931612	0.0866563	0.0560166	0.0147215	0.0707381	0.2628059	5.00E-06
robust	PD_HY3	psychencode	ENSG00000237818	RPS3AP29	3327	0.386021733	0.293289487	0.1739038	0.1320983	0.0146867	0.1467850	0.1111800	5.00E-06
robust	PD_HY3	psychencode	ENSG00000228414		2707	0.466371106	0.326191205	0.1134409	0.0793140	0.0146828	0.0939968	0.1851226	5.00E-06
robust	PD_HY3	psychencode	ENSG00000224551	HMGB3P21	2980	0.46656871	0.335630474	0.1065235	0.0765993	0.0146779	0.0912773	0.1916197	5.00E-06
robust	PD_HY3	psychencode	ENSG00000263819	RN7SL478P	3326	0.476075034	0.361342708	0.0841026	0.0638049	0.0146748	0.0784796	0.2299945	5.00E-06
liberal	PD_HY3	psychencode	ENSG00000224387		2079	0.001600737	0.180749799	0.0070497	0.7960184	0.0145813	0.8105997	0.0183178	1.00E-05
robust	PD_HY3	psychencode	ENSG00000232097		3287	0.455607766	0.344862484	0.1053363	0.0797031	0.0144904	0.0941935	0.1818047	5.00E-06
robust	PD_HY3	psychencode	ENSG00000205356	TECPR1	3395	0.439192295	0.333763181	0.1208117	0.0917816	0.0144512	0.1062329	0.1574521	5.00E-06
liberal	PD_HY3	eQTLGen	ENSG00000070669	ASNS	4609	1.36E-304	1.16E-304	0.5320730	0.4535547	0.0143723	0.4679270	0.0316880	1.00E-05
robust	PD_HY3	psychencode	ENSG00000105880	DLX5	2877	0.482457243	0.33567792	0.0987957	0.0687101	0.0143590	0.0830691	0.2089797	5.00E-06
liberal	PD_HY3	psychencode	ENSG00000270335		2457	0.680392904	0.135836728	0.1412897	0.0281934	0.0142873	0.0424807	0.5067582	1.00E-05
liberal	PD_HY3	psychencode	ENSG00000070669	ASNS	3383	9.89E-14	7.50E-14	0.5606487	0.4251474	0.0142039	0.4393513	0.0334093	1.00E-05
robust	PD_HY3	psychencode	ENSG00000164713	BRI3	3396	0.254939413	0.193755821	0.3052316	0.2319500	0.0141231	0.2460732	0.0608887	5.00E-06
robust	PD_HY3	psychencode	ENSG00000135175	OCM2	3318	0.406310806	0.30829762	0.1542595	0.1170197	0.0141123	0.1311320	0.1205977	5.00E-06
robust	PD_HY3	psychencode	ENSG00000237024		2687	0.487924159	0.341189119	0.0923127	0.0645231	0.0140510	0.0785741	0.2177668	5.00E-06
liberal	PD_HY3	psychencode	ENSG00000006128	TAC1	3320	0.000728663	0.000544916	0.5634124	0.4213227	0.0139913	0.4353140	0.0332081	1.00E-05
liberal	PD_HY3	psychencode	ENSG00000228541		2434	0.700794757	0.123151869	0.1379181	0.0242227	0.0139126	0.0381353	0.5743642	1.00E-05
robust	PD_HY3	psychencode	ENSG00000221245		3465	0.621265175	0.131260534	0.1928799	0.0407238	0.0138705	0.0545943	0.3406000	5.00E-06
liberal	PD_HY3	eQTLGen	ENSG00000183444	OR7E38P	4497	2.99E-47	2.54E-47	0.5335140	0.4528105	0.0136755	0.4664860	0.0302014	1.00E-05
liberal	PD_HY3	psychencode	ENSG00000250938		4018	0.57174812	0.12965307	0.2323264	0.0526701	0.0136023	0.0662724	0.2582546	1.00E-05
robust	PD_HY3	psychencode	ENSG00000230702		2576	0.511587252	0.331497792	0.0869884	0.0563395	0.0135871	0.0699266	0.2411650	5.00E-06
robust	PD_HY3	psychencode	ENSG00000225905		913	0.003268484	0.38485384	0.0050392	0.5933192	0.0135193	0.6068385	0.0227859	5.00E-06
robust	PD_HY3	psychencode	ENSG00000272950		3304	1.16E-24	8.70E-25	0.5629338	0.4236234	0.0134428	0.4370662	0.0317330	5.00E-06
liberal	PD_HY3	psychencode	ENSG00000250219	LTV1P1	3953	0.571218848	0.128459615	0.2342709	0.0526711	0.0133795	0.0660506	0.2540203	1.00E-05
robust	PD_HY3	psychencode	ENSG00000235982		3363	0.389186035	0.295206366	0.1718903	0.1303559	0.0133614	0.1437173	0.1024996	5.00E-06
robust	PD_HY3	eQTLGen	ENSG00000196636	ACN9	4165	3.04E-35	2.42E-35	0.5488318	0.4379936	0.0131746	0.4511682	0.0300794	5.00E-06
robust	PD_HY3	psychencode	ENSG00000242158	RN7SL361P	2616	0.42675456	0.288965179	0.1616657	0.1094412	0.0131734	0.1226146	0.1203699	5.00E-06
robust	PD_HY3	psychencode	ENSG00000236498		2574	0.516426579	0.333620924	0.0832050	0.0537259	0.0130216	0.0667475	0.2423712	5.00E-06
robust	PD_HY3	eQTLGen	ENSG00000180535	BHLHA15	4709	0.335151823	0.289909735	0.1941219	0.1678916	0.0129250	0.1808166	0.0769842	5.00E-06
liberal	PD_HY3	psychencode	ENSG00000249409		4254	0.572542566	0.182633578	0.1758353	0.0560762	0.0129123	0.0689886	0.2302641	1.00E-05
liberal	PD_HY3	psychencode	ENSG00000225665		4044	0.582424135	0.15507807	0.1971205	0.0524730	0.0129043	0.0653773	0.2459225	1.00E-05
robust	PD_HY3	psychencode	ENSG00000212978		2583	1.52E-20	1.04E-20	0.5846764	0.4024602	0.0128634	0.4153236	0.0319619	5.00E-06
robust	PD_HY3	psychencode	ENSG00000115421	PAPOLG	2665	0.473809918	0.329487399	0.1084995	0.0754250	0.0127782	0.0882032	0.1694164	5.00E-06
robust	PD_HY3	psychencode	ENSG00000162924	REL	2707	0.49944921	0.349326829	0.0814902	0.0569707	0.0127630	0.0697337	0.2240272	5.00E-06

robust	PD_HY3	eQTLGen	ENSG00000214389	RPS3AP26	4439	0.424574222	0.353208919	0.1143631	0.0951147	0.0127390	0.1078537	0.1339329	5.00E-06
robust	PD_HY3	psychencode	ENSG00000234624		2617	0.510084591	0.351627325	0.0743338	0.0512166	0.0127377	0.0639543	0.2487028	5.00E-06
robust	PD_HY3	psychencode	ENSG00000229839		2442	0.516643706	0.262768279	0.1378224	0.0700720	0.0126937	0.0827656	0.1811519	5.00E-06
liberal	PD_HY3	psychencode	ENSG00000267520		2699	0.000982892	0.000690323	0.5790459	0.4066738	0.0126071	0.4192809	0.0310005	1.00E-05
robust	PD_HY3	psychencode	ENSG00000271134		2615	0.45461688	0.307174892	0.1347550	0.0910262	0.0124270	0.1034532	0.1365207	5.00E-06
robust	PD_HY3	psychencode	ENSG00000178636		3410	0.414542225	0.073996167	0.4235080	0.0755718	0.0123818	0.0879536	0.1638422	5.00E-06
liberal	PD_HY3	eQTLGen	ENSG00000243554		4503	2.01E-19	1.71E-19	0.5341905	0.4535109	0.0122986	0.4658095	0.0271187	1.00E-05
robust	PD_HY3	psychencode	ENSG00000229831		2611	0.511275644	0.334993703	0.0855095	0.0560024	0.0122188	0.0682212	0.2181837	5.00E-06
robust	PD_HY3	eQTLGen	ENSG00000142611	PRDM16	4240	0.007707808	0.881917673	0.0008511	0.0973572	0.0121663	0.1095234	0.1249651	5.00E-06
robust	PD_HY3	eQTLGen	ENSG00000115464	USP34	3993	7.13E-64	8.49E-63	0.0764969	0.9114222	0.0120809	0.9235031	0.0132550	5.00E-06
liberal	PD_HY3	psychencode	ENSG00000233702		2428	0.714879127	0.124818949	0.1262194	0.0220260	0.0120565	0.0340825	0.5473731	1.00E-05
robust	PD_HY3	psychencode	ENSG00000115484	CCT4	2568	0.484354457	0.313163051	0.1156788	0.0747690	0.0120347	0.0868036	0.1609583	5.00E-06
robust	PD_HY3	psychencode	ENSG00000270820		2664	0.424800672	0.294304015	0.1589144	0.1100729	0.0119081	0.1219809	0.1081836	5.00E-06
robust	PD_HY3	eQTLGen	ENSG00000164715	LMTK2	4603	2.06E-182	1.76E-182	0.5321435	0.4560068	0.0118498	0.4678565	0.0259860	5.00E-06
robust	PD_HY3	psychencode	ENSG00000271243		2769	0.504430829	0.351687614	0.0778162	0.0542296	0.0118357	0.0660653	0.2182524	5.00E-06
robust	PD_HY3	psychencode	ENSG00000213495		2595	0.47539573	0.321327463	0.1142666	0.0772110	0.0117992	0.0890102	0.1528173	5.00E-06
robust	PD_HY3	psychencode	ENSG00000172399	MYOZ2	3455	1.66E-07	2.98E-08	0.8378870	0.1503738	0.0117389	0.1621128	0.0780650	5.00E-06
liberal	PD_HY3	psychencode	ENSG00000260091		3394	0.625069677	0.12298761	0.2007676	0.0394910	0.0116842	0.0511752	0.2958691	1.00E-05
robust	PD_HY3	psychencode	ENSG00000115464	USP34	2581	0.500602501	0.344089535	0.0851461	0.0585019	0.0116599	0.0701619	0.1993083	5.00E-06
liberal	PD_HY3	eQTLGen	ENSG00000189409	MMP23B	3070	0.004684818	0.570908946	0.0033599	0.4094340	0.0116124	0.4210464	0.0283620	1.00E-05
robust	PD_HY3	psychencode	ENSG00000238228	OR7E7P	3295	0.23959327	0.18154525	0.3227437	0.2445270	0.0115908	0.2561178	0.0474008	5.00E-06
robust	PD_HY3	psychencode	ENSG00000249244		3404	2.02E-97	3.96E-98	0.8262089	0.1622667	0.0115244	0.1737911	0.0710214	5.00E-06
robust	PD_HY3	psychencode	ENSG00000119866	BCL11A	2643	0.502389181	0.337631185	0.0888119	0.0596631	0.0115046	0.0711677	0.1928253	5.00E-06
robust	PD_HY3	eQTLGen	ENSG00000145390	USP53	4858	1.42E-304	2.18E-304	0.3896985	0.5988452	0.0114563	0.6103015	0.0191306	5.00E-06
robust	PD_HY3	eQTLGen	ENSG00000006453	BAIAP2L1	4559	7.01E-84	5.95E-84	0.5347307	0.4538195	0.0114498	0.4652693	0.0252299	5.00E-06
robust	PD_HY3	eQTLGen	ENSG00000238228	OR7E7P	4477	0.183481551	0.155574066	0.3515255	0.2980358	0.0113831	0.3094189	0.0381936	5.00E-06
liberal	PD_HY3	psychencode	ENSG00000248280		3369	0.596376822	0.11893777	0.2278718	0.0454340	0.0113796	0.0568136	0.2504645	1.00E-05
robust	PD_HY3	psychencode	ENSG00000213486		2733	0.491503798	0.34140266	0.0919067	0.0638164	0.0113704	0.0751868	0.1781736	5.00E-06
liberal	PD_HY3	psychencode	ENSG00000250392		3841	0.629567319	0.140543572	0.1787344	0.0398891	0.0112656	0.0511547	0.2824243	1.00E-05
robust	PD_HY3	psychencode	ENSG00000241524	RN7SL632P	2687	0.488525961	0.341197772	0.0936484	0.0653837	0.0112441	0.0766278	0.1719711	5.00E-06
liberal	PD_HY3	psychencode	ENSG00000170340	B3GNT2	2393	0.70320871	0.143928643	0.1178415	0.0241082	0.0109130	0.0350211	0.4526657	1.00E-05
robust	PD_HY3	psychencode	ENSG00000250950		3370	1.91E-17	3.82E-18	0.8246107	0.1645026	0.0108866	0.1753893	0.0661792	5.00E-06
robust	PD_HY3	eQTLGen	ENSG00000164713	BRI3	4686	2.16E-117	1.86E-117	0.5315763	0.4575868	0.0108369	0.4684237	0.0236828	5.00E-06
liberal	PD_HY3	psychencode	ENSG00000172403	SYNPO2	3367	0.581450838	0.100774797	0.2616143	0.0453311	0.0108290	0.0561601	0.2388858	1.00E-05

liberal	PD_HY3	psychencode	ENSG00000173209	AHSA2	2586	1.60E-47	1.10E-47	0.5859557	0.4032314	0.0108129	0.4140443	0.0268157	1.00E-05
robust	PD_HY3	psychencode	ENSG00000245958		3369	5.99E-216	1.19E-216	0.8246994	0.1645064	0.0107942	0.1753006	0.0656158	5.00E-06
robust	PD_HY3	eQTLGen	ENSG00000164109	MAD2L1	5638	7.64E-29	1.30E-28	0.3665733	0.6226488	0.0107780	0.6334267	0.0173099	5.00E-06
liberal	PD_HY3	psychencode	ENSG00000271657		2403	0.708917352	0.145514773	0.1118873	0.0229556	0.0107250	0.0336806	0.4672046	1.00E-05
robust	PD_HY3	psychencode	ENSG00000242735		2443	0.470455985	0.239286557	0.1853799	0.0942680	0.0106096	0.1048776	0.1125476	5.00E-06
liberal	PD_HY3	psychencode	ENSG00000241625	RN7SL18P	2423	0.710893942	0.12409432	0.1315476	0.0229526	0.0105116	0.0334642	0.4579714	1.00E-05
liberal	PD_HY3	eQTLGen	ENSG00000164096	C4orf3	4840	7.26E-260	1.19E-259	0.3744798	0.6152303	0.0102899	0.6255202	0.0167253	1.00E-05
liberal	PD_HY3	psychencode	ENSG00000271889		2584	0.014228679	0.009795228	0.5719902	0.3937561	0.0102298	0.4039859	0.0259801	1.00E-05
robust	PD_HY3	psychencode	ENSG00000170264	FAM161A	2580	0.062655595	0.040609217	0.5379097	0.3486171	0.0102084	0.3588255	0.0292827	5.00E-06
robust	PD_HY3	eQTLGen	ENSG00000106236	NPTX2	4176	5.71E-12	4.75E-12	0.5405419	0.4496033	0.0098548	0.4594581	0.0219190	5.00E-06
robust	PD_HY3	psychencode	ENSG00000273302		2588	0.037227788	0.025612506	0.5493892	0.3779570	0.0098135	0.3877705	0.0259646	5.00E-06
robust	PD_HY3	eQTLGen	ENSG00000260091		4848	7.21E-300	2.55E-300	0.7316115	0.2587508	0.0096377	0.2683885	0.0372470	5.00E-06
liberal	PD_HY3	psychencode	ENSG00000239958	RN7SL51P	2402	0.726237566	0.147081486	0.0974021	0.0197168	0.0095620	0.0292789	0.4849681	1.00E-05
robust	PD_HY3	eQTLGen	ENSG00000197530	MIB2	3046	0.006438793	0.785670749	0.0016125	0.1967444	0.0095335	0.2062779	0.0484564	5.00E-06
liberal	PD_HY3	psychencode	ENSG00000237651	C2orf74	2583	1.57E-284	1.08E-284	0.5866872	0.4038376	0.0094753	0.4133128	0.0234631	1.00E-05
liberal	PD_HY3	psychencode	ENSG00000162929	KIAA1841	2595	7.17E-16	4.94E-16	0.5868170	0.4037305	0.0094524	0.4131830	0.0234128	1.00E-05
robust	PD_HY3	psychencode	ENSG00000186889	TMEM17	2427	3.32E-54	5.79E-55	0.8434912	0.1472192	0.0092896	0.1565088	0.0631004	5.00E-06
liberal	PD_HY3	psychencode	ENSG00000253825		4015	0.659353285	0.149481283	0.1482900	0.0336094	0.0092661	0.0428755	0.2756986	1.00E-05
robust	PD_HY3	psychencode	ENSG00000227775		1248	0.00171252	0.198935552	0.0067443	0.7834334	0.0091743	0.7926076	0.0117103	5.00E-06
robust	PD_HY3	psychencode	ENSG00000082898	XPO1	2744	9.31E-08	6.49E-08	0.5840154	0.4068138	0.0091706	0.4159844	0.0225425	5.00E-06
liberal	PD_HY3	psychencode	ENSG00000249002		3477	0.699026704	0.124973047	0.1415891	0.0253044	0.0091067	0.0344111	0.3598860	1.00E-05
liberal	PD_HY3	psychencode	ENSG00000145384	FABP2	3367	0.671598046	0.124677818	0.1644085	0.0305126	0.0088031	0.0393157	0.2885070	1.00E-05
robust	PD_HY3	eQTLGen	ENSG00000250412	KLHL2P1	4849	0.090154667	0.143740487	0.2919116	0.4653994	0.0087938	0.4741933	0.0188953	5.00E-06
robust	PD_HY3	psychencode	ENSG00000164109	MAD2L1	4018	0.008116608	0.001840571	0.7998995	0.1813725	0.0087708	0.1901433	0.0483578	5.00E-06
robust	PD_HY3	eQTLGen	ENSG00000248280		4844	4.43E-300	2.23E-300	0.6599531	0.3312880	0.0087589	0.3400469	0.0264390	5.00E-06
liberal	PD_HY3	eQTLGen	ENSG00000245958		4938	4.31E-179	7.34E-179	0.3671960	0.6242734	0.0085307	0.6328040	0.0136649	1.00E-05
robust	PD_HY3	psychencode	ENSG00000150961	SEC24D	3355	0.265550312	0.040676588	0.5943268	0.0910212	0.0084251	0.0994463	0.0925616	5.00E-06
robust	PD_HY3	eQTLGen	ENSG00000178636		4918	5.92E-50	1.61E-50	0.7795639	0.2121948	0.0082412	0.2204361	0.0388381	5.00E-06
robust	PD_HY3	psychencode	ENSG00000243554		3310	0.027471093	0.020836384	0.5366880	0.4070533	0.0079513	0.4150046	0.0195337	5.00E-06
robust	PD_HY3	psychencode	ENSG00000145388	METTL14	3385	0.08178059	0.011804926	0.7852643	0.1133363	0.0078139	0.1211502	0.0689440	5.00E-06
robust	PD_HY3	psychencode	ENSG00000162928	PEX13	2632	5.55E-18	3.90E-18	0.5828735	0.4093708	0.0077558	0.4171265	0.0189456	5.00E-06
robust	PD_HY3	psychencode	ENSG00000183444	OR7E38P	3306	0.004816152	0.003652038	0.5595972	0.4243215	0.0076130	0.4319346	0.0179416	5.00E-06
liberal	PD_HY3	eQTLGen	ENSG00000145388	METTL14	4764	1.16E-59	1.62E-59	0.4137137	0.5787027	0.0075836	0.5862863	0.0131045	1.00E-05
robust	PD_HY3	psychencode	ENSG00000224387		2079	0.001612493	0.182077261	0.0071015	0.8018645	0.0073442	0.8092087	0.0091589	5.00E-06

robust	PD_HY3	eQTLGen	ENSG00000070669	ASNS	4609	1.37E-304	1.17E-304	0.5359242	0.4568376	0.0072381	0.4640758	0.0158440	5.00E-06
robust	PD_HY3	psychencode	ENSG00000270335		2457	0.685288347	0.136814077	0.1423063	0.0283963	0.0071950	0.0355913	0.2533791	5.00E-06
robust	PD_HY3	psychencode	ENSG00000070669	ASNS	3383	9.96E-14	7.56E-14	0.5646589	0.4281884	0.0071527	0.4353411	0.0167046	5.00E-06
robust	PD_HY3	psychencode	ENSG00000006128	TAC1	3320	0.000733797	0.000548755	0.5673816	0.4242909	0.0070449	0.4313358	0.0166041	5.00E-06
robust	PD_HY3	psychencode	ENSG00000228541		2434	0.705703856	0.124014554	0.1388842	0.0243923	0.0070050	0.0313974	0.2871821	5.00E-06
liberal	PD_HY3	eQTLGen	ENSG00000170264	FAM161A	3914	7.24E-08	8.57E-07	0.0773776	0.9156313	0.0069902	0.9226215	0.0076343	1.00E-05
robust	PD_HY3	eQTLGen	ENSG00000183444	OR7E38P	4497	3.01E-47	2.56E-47	0.5371871	0.4559281	0.0068848	0.4628129	0.0151007	5.00E-06
robust	PD_HY3	psychencode	ENSG00000250938		4018	0.575663293	0.130540899	0.2339173	0.0530308	0.0068477	0.0598785	0.1291273	5.00E-06
liberal	PD_HY3	eQTLGen	ENSG00000172403	SYNPO2	4943	5.74E-66	8.65E-66	0.3959878	0.5972764	0.0067359	0.6040122	0.0112776	1.00E-05
robust	PD_HY3	psychencode	ENSG00000250219	LTV1P1	3953	0.575065902	0.129324767	0.2358487	0.0530258	0.0067348	0.0597606	0.1270102	5.00E-06
robust	PD_HY3	psychencode	ENSG00000249409		4254	0.57626302	0.183820354	0.1769779	0.0564406	0.0064981	0.0629388	0.1151321	5.00E-06
robust	PD_HY3	psychencode	ENSG00000225665		4044	0.586206426	0.156085154	0.1984006	0.0528138	0.0064940	0.0593078	0.1229613	5.00E-06
robust	PD_HY3	psychencode	ENSG00000267520		2699	0.000989127	0.000694703	0.5827191	0.4092536	0.0063435	0.4155971	0.0155003	5.00E-06
robust	PD_HY3	eQTLGen	ENSG00000243554		4503	2.03E-19	1.72E-19	0.5374957	0.4563169	0.0061874	0.4625043	0.0135593	5.00E-06
robust	PD_HY3	psychencode	ENSG00000233702		2428	0.719214719	0.12557595	0.1269849	0.0221596	0.0060648	0.0282244	0.2736866	5.00E-06
liberal	PD_HY3	psychencode	ENSG00000178821	TMEM52	1498	0.000625398	0.071568032	0.0079858	0.9138544	0.0059664	0.9198208	0.0065288	1.00E-05
robust	PD_HY3	psychencode	ENSG00000260091		3394	0.628742844	0.123710336	0.2019474	0.0397231	0.0058764	0.0455995	0.1479345	5.00E-06
robust	PD_HY3	eQTLGen	ENSG00000189409	MMP23B	3070	0.004712178	0.574243103	0.0033795	0.4118251	0.0058401	0.4176652	0.0141810	5.00E-06
robust	PD_HY3	psychencode	ENSG00000248280		3369	0.599789504	0.119618375	0.2291758	0.0456940	0.0057224	0.0514163	0.1252322	5.00E-06
robust	PD_HY3	psychencode	ENSG00000250392		3841	0.63313365	0.141339713	0.1797468	0.0401151	0.0056647	0.0457798	0.1412121	5.00E-06
robust	PD_HY3	psychencode	ENSG00000170340	B3GNT2	2393	0.707066803	0.144718295	0.1184880	0.0242405	0.0054864	0.0297269	0.2263328	5.00E-06
robust	PD_HY3	psychencode	ENSG00000172403	SYNPO2	3367	0.584616232	0.101323411	0.2630385	0.0455779	0.0054440	0.0510219	0.1194429	5.00E-06
robust	PD_HY3	psychencode	ENSG00000173209	AHSA2	2586	1.61E-47	1.11E-47	0.5891409	0.4054233	0.0054359	0.4108591	0.0134078	5.00E-06
robust	PD_HY3	psychencode	ENSG00000271657		2403	0.712739409	0.146299301	0.1124905	0.0230794	0.0053914	0.0284708	0.2336023	5.00E-06
robust	PD_HY3	psychencode	ENSG00000241625	RN7SL18P	2423	0.714650005	0.124749982	0.1322426	0.0230738	0.0052836	0.0283574	0.2289857	5.00E-06
liberal	PD_HY3	eQTLGen	ENSG00000162928	PEX13	4173	2.53E-17	3.04E-16	0.0764220	0.9183169	0.0052611	0.9235780	0.0057291	1.00E-05
robust	PD_HY3	eQTLGen	ENSG00000164096	C4orf3	4840	7.29E-260	1.20E-259	0.3764165	0.6184120	0.0051716	0.6235835	0.0083627	5.00E-06
robust	PD_HY3	psychencode	ENSG00000271889		2584	0.014301832	0.009845588	0.5749309	0.3957805	0.0051412	0.4009217	0.0129900	5.00E-06
robust	PD_HY3	psychencode	ENSG00000239958	RN7SL51P	2402	0.7297264	0.147788063	0.0978700	0.0198115	0.0048040	0.0246155	0.2424840	5.00E-06
robust	PD_HY3	psychencode	ENSG00000237651	C2orf74	2583	1.57E-284	1.08E-284	0.5894799	0.4057599	0.0047602	0.4105201	0.0117315	5.00E-06
robust	PD_HY3	psychencode	ENSG00000162929	KIAA1841	2595	7.21E-16	4.96E-16	0.5896036	0.4056477	0.0047487	0.4103964	0.0117064	5.00E-06
robust	PD_HY3	psychencode	ENSG00000253825		4015	0.662422309	0.150177058	0.1489802	0.0337658	0.0046546	0.0384204	0.1378493	5.00E-06
robust	PD_HY3	psychencode	ENSG00000249002		3477	0.702224178	0.125544696	0.1422368	0.0254202	0.0045742	0.0299943	0.1799430	5.00E-06
robust	PD_HY3	psychencode	ENSG00000145384	FABP2	3367	0.674567183	0.125229019	0.1651353	0.0306475	0.0044210	0.0350685	0.1442535	5.00E-06

robust	PD_HY3	eQTLGen	ENSG00000245958		4938	4.33E-179	7.37E-179	0.3687689	0.6269475	0.0042836	0.6312311	0.0068325	5.00E-06
robust	PD_HY3	eQTLGen	ENSG00000145388	METTL14	4764	1.17E-59	1.63E-59	0.4152884	0.5809054	0.0038062	0.5847116	0.0065522	5.00E-06
liberal	PD_HY3	eQTLGen	ENSG00000119866	BCL11A	4561	2.41E-05	8.78E-05	0.2146097	0.7816835	0.0035949	0.7852784	0.0045989	1.00E-05
liberal	PD_HY3	eQTLGen	ENSG00000267520		4276	5.30E-22	6.37E-21	0.0765830	0.9198663	0.0035507	0.9234170	0.0038600	1.00E-05
liberal	PD_HY3	psychencode	ENSG00000008128	CDK11A	1248	0.000233238	0.02709417	0.0082715	0.9608601	0.0035410	0.9644011	0.0036852	1.00E-05
robust	PD_HY3	eQTLGen	ENSG00000170264	FAM161A	3914	7.27E-08	8.60E-07	0.0776490	0.9188427	0.0035073	0.9223501	0.0038171	5.00E-06
robust	PD_HY3	eQTLGen	ENSG00000172403	SYNPO2	4943	5.76E-66	8.68E-66	0.3973259	0.5992947	0.0033793	0.6026741	0.0056388	5.00E-06
robust	PD_HY3	psychencode	ENSG00000178821	TMEM52	1498	0.000627269	0.071782173	0.0080097	0.9165888	0.0029921	0.9195809	0.0032644	5.00E-06
liberal	PD_HY3	eQTLGen	ENSG00000228541		3661	1.50E-15	1.36E-14	0.0991268	0.8981800	0.0026932	0.9008732	0.0029985	1.00E-05
robust	PD_HY3	eQTLGen	ENSG00000162928	PEX13	4173	2.53E-17	3.04E-16	0.0766235	0.9207390	0.0026375	0.9233765	0.0028645	5.00E-06
liberal	PD_HY3	eQTLGen	ENSG00000078369	GNB1	3278	0.000977368	0.118400654	0.0071894	0.8709351	0.0024975	0.8734326	0.0028677	1.00E-05
liberal	PD_HY3	eQTLGen	ENSG00000173163	COMMD1	3819	1.61E-20	1.87E-19	0.0790508	0.9184532	0.0024960	0.9209492	0.0027176	1.00E-05
liberal	PD_HY3	eQTLGen	ENSG00000237522	NONOP2	4296	1.61E-05	0.000193411	0.0766567	0.9209285	0.0022053	0.9231338	0.0023947	1.00E-05
liberal	PD_HY3	eQTLGen	ENSG00000162929	KIAA1841	4104	5.89E-306	7.05E-305	0.0769997	0.9208340	0.0021663	0.9230003	0.0023526	1.00E-05
liberal	PD_HY3	eQTLGen	ENSG00000186889	TMEM17	3679	4.34E-06	3.74E-05	0.1036644	0.8941505	0.0021433	0.8962938	0.0023970	1.00E-05
liberal	PD_HY3	eQTLGen	ENSG00000115484	CCT4	3895	2.22E-132	2.63E-131	0.0777781	0.9201806	0.0020414	0.9222219	0.0022184	1.00E-05
liberal	PD_HY3	eQTLGen	ENSG00000173209	AHSA2	4087	3.64E-305	4.35E-304	0.0770748	0.9209978	0.0019274	0.9229252	0.0020927	1.00E-05
liberal	PD_HY3	eQTLGen	ENSG00000082898	XPO1	4108	4.84E-07	5.77E-06	0.0772832	0.9208376	0.0018730	0.9227105	0.0020340	1.00E-05
robust	PD_HY3	eQTLGen	ENSG00000119866	BCL11A	4561	2.41E-05	8.80E-05	0.2149962	0.7830910	0.0018007	0.7848917	0.0022995	5.00E-06
robust	PD_HY3	eQTLGen	ENSG00000267520		4276	5.31E-22	6.38E-21	0.0767192	0.9215023	0.0017785	0.9232808	0.0019300	5.00E-06
robust	PD_HY3	psychencode	ENSG00000008128	CDK11A	1248	0.000233652	0.027142225	0.0082862	0.9625643	0.0017736	0.9643379	0.0018426	5.00E-06
liberal	PD_HY3	eQTLGen	ENSG00000205090	TMEM240	2835	0.00012071	0.014894486	0.0079052	0.9754177	0.0016620	0.9770797	0.0017038	1.00E-05
liberal	PD_HY3	eQTLGen	ENSG00000170340	B3GNT2	3668	9.36E-305	9.52E-304	0.0894255	0.9089629	0.0016116	0.9105745	0.0017730	1.00E-05
liberal	PD_HY3	psychencode	ENSG00000175756	AURKAIP1	862	9.31E-05	0.005743076	0.0158375	0.9767179	0.0016084	0.9783263	0.0016468	1.00E-05
liberal	PD_HY3	eQTLGen	ENSG00000232713		4272	7.34E-11	8.82E-10	0.0767344	0.9217852	0.0014804	0.9232656	0.0016060	1.00E-05
liberal	PD_HY3	eQTLGen	ENSG00000175756	AURKAIP1	2415	0.000302502	0.01964862	0.0148387	0.9638279	0.0013823	0.9652102	0.0014342	1.00E-05
robust	PD_HY3	eQTLGen	ENSG00000228541		3661	1.51E-15	1.36E-14	0.0992605	0.8993911	0.0013484	0.9007395	0.0014992	5.00E-06
robust	PD_HY3	eQTLGen	ENSG00000078369	GNB1	3278	0.00097859	0.118548695	0.0071984	0.8720240	0.0012503	0.8732744	0.0014338	5.00E-06
robust	PD_HY3	eQTLGen	ENSG00000173163	COMMD1	3819	1.61E-20	1.87E-19	0.0791496	0.9196008	0.0012496	0.9208504	0.0013588	5.00E-06
liberal	PD_HY3	eQTLGen	ENSG00000212978		4076	9.04E-125	1.08E-123	0.0771040	0.9216621	0.0012339	0.9228960	0.0013388	1.00E-05
robust	PD_HY3	eQTLGen	ENSG00000237522	NONOP2	4296	1.61E-05	0.000193625	0.0767413	0.9219451	0.0011039	0.9230490	0.0011973	5.00E-06
robust	PD_HY3	eQTLGen	ENSG00000162929	KIAA1841	4104	5.90E-306	7.05E-305	0.0770832	0.9218325	0.0010843	0.9229168	0.0011763	5.00E-06
robust	PD_HY3	eQTLGen	ENSG00000186889	TMEM17	3679	4.34E-06	3.75E-05	0.1037757	0.8951097	0.0010728	0.8961825	0.0011985	5.00E-06
liberal	PD_HY3	eQTLGen	ENSG00000221978	CCNL2	2461	0.000218754	0.026198256	0.0080536	0.9645037	0.0010258	0.9655294	0.0010635	1.00E-05

robust	PD_HY3	eQTLGen	ENSG00000115484	CCT4	3895	2.22E-132	2.63E-131	0.0778575	0.9211207	0.0010217	0.9221425	0.0011092	5.00E-06
robust	PD_HY3	eQTLGen	ENSG00000173209	AHSA2	4087	3.64E-305	4.35E-304	0.0771492	0.9218862	0.0009646	0.9228508	0.0010463	5.00E-06
robust	PD_HY3	eQTLGen	ENSG00000082898	XPO1	4108	4.85E-07	5.78E-06	0.0773557	0.9217007	0.0009374	0.9226381	0.0010170	5.00E-06
robust	PD_HY3	eQTLGen	ENSG00000205090	TMEM240	2835	0.000120811	0.014906873	0.0079117	0.9762289	0.0008317	0.9770606	0.0008519	5.00E-06
robust	PD_HY3	eQTLGen	ENSG00000170340	B3GNT2	3668	9.37E-305	9.53E-304	0.0894976	0.9096959	0.0008064	0.9105024	0.0008865	5.00E-06
robust	PD_HY3	psychencode	ENSG00000175756	AURKAIP1	862	9.32E-05	0.005747698	0.0158502	0.9775040	0.0008049	0.9783089	0.0008234	5.00E-06
liberal	PD_HY3	psychencode	ENSG00000235098	ANKRD65	917	2.55E-92	3.00E-90	0.0084180	0.9907987	0.0007834	0.9915820	0.0007906	1.00E-05
liberal	PD_HY3	eQTLGen	ENSG00000008128	CDK11A	3089	2.26E-19	2.75E-17	0.0081310	0.9911106	0.0007584	0.9918690	0.0007652	1.00E-05
robust	PD_HY3	eQTLGen	ENSG00000232713		4272	7.34E-11	8.82E-10	0.0767912	0.9224680	0.0007407	0.9232088	0.0008030	5.00E-06
robust	PD_HY3	eQTLGen	ENSG00000175756	AURKAIP1	2415	0.000302711	0.01966221	0.0148490	0.9644945	0.0006916	0.9651861	0.0007171	5.00E-06
liberal	PD_HY3	eQTLGen	ENSG00000231050		3298	2.71E-215	3.27E-213	0.0082308	0.9910780	0.0006913	0.9917692	0.0006975	1.00E-05
liberal	PD_HY3	eQTLGen	ENSG00000235098	ANKRD65	2530	1.36E-22	1.70E-20	0.0079578	0.9913955	0.0006466	0.9920422	0.0006522	1.00E-05
liberal	PD_HY3	eQTLGen	ENSG00000269227		3059	1.99E-43	2.42E-41	0.0081363	0.9912189	0.0006448	0.9918637	0.0006505	1.00E-05
liberal	PD_HY3	eQTLGen	ENSG00000269737		3059	1.51E-49	1.85E-47	0.0081363	0.9912189	0.0006448	0.9918637	0.0006505	1.00E-05
robust	PD_HY3	eQTLGen	ENSG00000212978		4076	9.04E-125	1.08E-123	0.0771516	0.9222310	0.0006173	0.9228484	0.0006694	5.00E-06
liberal	PD_HY3	eQTLGen	ENSG00000160072	ATAD3B	2724	5.58E-33	6.90E-31	0.0080131	0.9913940	0.0005930	0.9919869	0.0005981	1.00E-05
liberal	PD_HY3	eQTLGen	ENSG00000215915	ATAD3C	2672	1.60E-17	1.99E-15	0.0080008	0.9914072	0.0005920	0.9919992	0.0005971	1.00E-05
liberal	PD_HY3	eQTLGen	ENSG00000157933	SKI	3692	7.34E-16	8.54E-14	0.0085178	0.9909244	0.0005578	0.9914822	0.0005629	1.00E-05
liberal	PD_HY3	psychencode	ENSG00000271847		1989	3.43E-05	0.0038758	0.0087278	0.9868060	0.0005562	0.9873622	0.0005636	1.00E-05
robust	PD_HY3	eQTLGen	ENSG00000221978	CCNL2	2461	0.000218866	0.0262117	0.0080577	0.9649986	0.0005132	0.9655117	0.0005318	5.00E-06
liberal	PD_HY3	eQTLGen	ENSG00000225931		3781	1.20E-12	1.40E-10	0.0085073	0.9909836	0.0005091	0.9914927	0.0005137	1.00E-05
liberal	PD_HY3	eQTLGen	ENSG00000248333	CDK11B	3066	2.80E-55	3.41E-53	0.0081371	0.9913840	0.0004788	0.9918629	0.0004830	1.00E-05
liberal	PD_HY3	eQTLGen	ENSG00000008130	NADK	3100	1.81E-06	0.000220581	0.0081301	0.9911688	0.0004787	0.9916475	0.0004829	1.00E-05
liberal	PD_HY3	eQTLGen	ENSG00000228037		3788	4.93E-26	5.74E-24	0.0085065	0.9910158	0.0004777	0.9914935	0.0004821	1.00E-05
liberal	PD_HY3	eQTLGen	ENSG00000224870		2474	1.77E-55	2.17E-53	0.0080797	0.9914637	0.0004566	0.9919203	0.0004606	1.00E-05
liberal	PD_HY3	psychencode	ENSG00000268575		1248	6.70E-104	7.78E-102	0.0085311	0.9910201	0.0004488	0.9914689	0.0004528	1.00E-05
liberal	PD_HY3	eQTLGen	ENSG00000268575		3094	1.37E-181	1.67E-179	0.0081337	0.9914416	0.0004247	0.9918663	0.0004284	1.00E-05
robust	PD_HY3	psychencode	ENSG00000235098	ANKRD65	917	2.55E-92	3.00E-90	0.0084213	0.9911869	0.0003918	0.9915787	0.0003953	5.00E-06
liberal	PD_HY3	psychencode	ENSG00000224870		887	3.61E-07	4.17E-05	0.0085816	0.9909917	0.0003847	0.9913764	0.0003882	1.00E-05
robust	PD_HY3	eQTLGen	ENSG00000008128	CDK11A	3089	2.26E-19	2.75E-17	0.0081341	0.9914865	0.0003793	0.9918659	0.0003826	5.00E-06
liberal	PD_HY3	eQTLGen	ENSG00000215912	TTC34	4067	6.03E-32	7.01E-30	0.0085340	0.9911075	0.0003585	0.9914660	0.0003617	1.00E-05
robust	PD_HY3	eQTLGen	ENSG00000231050		3298	2.72E-215	3.27E-213	0.0082336	0.9914206	0.0003458	0.9917664	0.0003488	5.00E-06
liberal	PD_HY3	eQTLGen	ENSG00000215790	SLC35E2	3089	1.71E-23	2.08E-21	0.0081345	0.9915387	0.0003267	0.9918655	0.0003295	1.00E-05
robust	PD_HY3	eQTLGen	ENSG00000235098	ANKRD65	2530	1.36E-22	1.70E-20	0.0079604	0.9917162	0.0003234	0.9920396	0.0003261	5.00E-06

robust	PD_HY3	eQTLGen	ENSG00000269227		3059	1.99E-43	2.42E-41	0.0081389	0.9915386	0.0003225	0.9918611	0.0003253	5.00E-06
robust	PD_HY3	eQTLGen	ENSG00000269737		3059	1.52E-49	1.85E-47	0.0081389	0.9915386	0.0003225	0.9918611	0.0003253	5.00E-06
robust	PD_HY3	eQTLGen	ENSG00000160072	ATAD3B	2724	5.58E-33	6.90E-31	0.0080154	0.9916880	0.0002966	0.9919846	0.0002991	5.00E-06
robust	PD_HY3	eQTLGen	ENSG00000215915	ATAD3C	2672	1.60E-17	1.99E-15	0.0080032	0.9917008	0.0002961	0.9919968	0.0002986	5.00E-06
liberal	PD_HY3	psychencode	ENSG00000215912	TTC34	2207	8.58E-06	0.000973179	0.0087293	0.9900017	0.0002873	0.9902890	0.0002902	1.00E-05
robust	PD_HY3	eQTLGen	ENSG00000157933	SKI	3692	7.35E-16	8.55E-14	0.0085202	0.9912009	0.0002790	0.9914798	0.0002815	5.00E-06
robust	PD_HY3	psychencode	ENSG00000271847		1989	3.43E-05	0.003876878	0.0087302	0.9870805	0.0002782	0.9873587	0.0002818	5.00E-06
liberal	PD_HY3	eQTLGen	ENSG00000189339	SLC35E2B	3082	1.38E-65	1.69E-63	0.0081373	0.9915928	0.0002699	0.9918627	0.0002722	1.00E-05
liberal	PD_HY3	eQTLGen	ENSG00000227775		3093	2.81E-21	3.43E-19	0.0081351	0.9916082	0.0002567	0.9918649	0.0002589	1.00E-05
robust	PD_HY3	eQTLGen	ENSG00000225931		3781	1.20E-12	1.40E-10	0.0085095	0.9912359	0.0002546	0.9914905	0.0002568	5.00E-06
liberal	PD_HY3	psychencode	ENSG00000215915	ATAD3C	969	8.59E-08	1.01E-05	0.0084384	0.9913029	0.0002486	0.9915515	0.0002508	1.00E-05
robust	PD_HY3	eQTLGen	ENSG00000248333	CDK11B	3066	2.80E-55	3.41E-53	0.0081391	0.9916214	0.0002395	0.9918609	0.0002415	5.00E-06
robust	PD_HY3	eQTLGen	ENSG00000008130	NADK	3100	1.81E-06	0.000220634	0.0081321	0.9914061	0.0002394	0.9916455	0.0002415	5.00E-06
robust	PD_HY3	eQTLGen	ENSG00000228037		3788	4.93E-26	5.74E-24	0.0085085	0.9912526	0.0002389	0.9914915	0.0002410	5.00E-06
robust	PD_HY3	eQTLGen	ENSG00000224870		2474	1.77E-55	2.17E-53	0.0080815	0.9916901	0.0002284	0.9919185	0.0002303	5.00E-06
liberal	PD_HY3	psychencode	ENSG00000215790	SLC35E2	1248	1.60E-36	1.85E-34	0.0085330	0.9912400	0.0002270	0.9914670	0.0002290	1.00E-05
liberal	PD_HY3	eQTLGen	ENSG00000142606	MMEL1	3865	8.57E-237	9.97E-235	0.0085186	0.9912547	0.0002267	0.9914814	0.0002287	1.00E-05
liberal	PD_HY3	psychencode	ENSG00000160075	SSU72	1141	6.89E-17	8.06E-15	0.0084784	0.9912958	0.0002259	0.9915216	0.0002278	1.00E-05
robust	PD_HY3	psychencode	ENSG00000268575		1248	6.70E-104	7.78E-102	0.0085330	0.9912425	0.0002244	0.9914670	0.0002264	5.00E-06
liberal	PD_HY3	eQTLGen	ENSG00000178642		3732	5.35E-52	6.21E-50	0.0085432	0.9912333	0.0002235	0.9914568	0.0002255	1.00E-05
liberal	PD_HY3	psychencode	ENSG00000116151	MORN1	2025	4.96E-07	5.60E-05	0.0087849	0.9909446	0.0002140	0.9911586	0.0002159	1.00E-05
liberal	PD_HY3	psychencode	ENSG00000157870	FAM213B	2147	3.33E-07	3.77E-05	0.0087466	0.9910029	0.0002125	0.9912153	0.0002144	1.00E-05
robust	PD_HY3	eQTLGen	ENSG00000268575		3094	1.37E-181	1.67E-179	0.0081354	0.9916522	0.0002124	0.9918646	0.0002142	5.00E-06
liberal	PD_HY3	eQTLGen	ENSG00000160075	SSU72	2871	5.40E-306	6.66E-304	0.0080510	0.9917366	0.0002124	0.9919490	0.0002141	1.00E-05
liberal	PD_HY3	psychencode	ENSG00000162585	C1orf86	1927	5.55E-15	6.28E-13	0.0087481	0.9910468	0.0002051	0.9912519	0.0002070	1.00E-05
liberal	PD_HY3	eQTLGen	ENSG00000149527	PLCH2	3746	8.72E-305	1.01E-302	0.0085421	0.9912543	0.0002036	0.9914579	0.0002054	1.00E-05
liberal	PD_HY3	eQTLGen	ENSG00000169885	CALML6	3417	2.43E-29	2.90E-27	0.0082864	0.9915116	0.0002020	0.9917136	0.0002037	1.00E-05
liberal	PD_HY3	eQTLGen	ENSG00000197785	ATAD3A	2793	3.90E-39	4.83E-37	0.0080223	0.9917844	0.0001933	0.9919777	0.0001949	1.00E-05
liberal	PD_HY3	eQTLGen	ENSG00000187730	GABRD	3568	1.29E-29	1.52E-27	0.0083860	0.9914207	0.0001933	0.9916140	0.0001950	1.00E-05
robust	PD_HY3	psychencode	ENSG00000224870		887	3.61E-07	4.17E-05	0.0085832	0.9911823	0.0001924	0.9913747	0.0001941	5.00E-06
liberal	PD_HY3	eQTLGen	ENSG00000224387		3723	4.65E-29	5.40E-27	0.0085353	0.9912726	0.0001921	0.9914647	0.0001938	1.00E-05
liberal	PD_HY3	eQTLGen	ENSG00000228594	C1orf233	2982	2.19E-08	2.68E-06	0.0081003	0.9917079	0.0001891	0.9918970	0.0001907	1.00E-05
liberal	PD_HY3	eQTLGen	ENSG00000157916	RER1	3807	2.21E-21	2.56E-19	0.0085525	0.9912589	0.0001885	0.9914475	0.0001902	1.00E-05
liberal	PD_HY3	eQTLGen	ENSG00000215014		2882	1.86E-103	2.29E-101	0.0080672	0.9917471	0.0001857	0.9919328	0.0001872	1.00E-05

liberal	PD_HY3	eQTLGen	ENSG00000269554		3729	1.06E-08	1.23E-06	0.0085093	0.9913045	0.0001850	0.9914895	0.0001867	1.00E-05
liberal	PD_HY3	eQTLGen	ENSG00000162585	C1orf86	3755	1.28E-299	1.49E-297	0.0085095	0.9913061	0.0001845	0.9914905	0.0001861	1.00E-05
robust	PD_HY3	eQTLGen	ENSG00000215912	TTC34	4067	6.03E-32	7.01E-30	0.0085355	0.9912852	0.0001793	0.9914645	0.0001809	5.00E-06
liberal	PD_HY3	eQTLGen	ENSG00000229393		3754	5.27E-18	6.12E-16	0.0085335	0.9912888	0.0001777	0.9914665	0.0001793	1.00E-05
liberal	PD_HY3	psychencode	ENSG00000157911	PEX10	2050	2.05E-10	2.31E-08	0.0087893	0.9910392	0.0001714	0.9912107	0.0001730	1.00E-05
robust	PD_HY3	eQTLGen	ENSG00000215790	SLC35E2	3089	1.71E-23	2.08E-21	0.0081359	0.9917007	0.0001634	0.9918641	0.0001648	5.00E-06
liberal	PD_HY3	eQTLGen	ENSG00000157881	PANK4	3801	1.21E-86	1.41E-84	0.0085276	0.9913090	0.0001634	0.9914724	0.0001648	1.00E-05
liberal	PD_HY3	eQTLGen	ENSG00000182873		3731	4.17E-79	4.86E-77	0.0085090	0.9913311	0.0001599	0.9914910	0.0001613	1.00E-05
liberal	PD_HY3	eQTLGen	ENSG00000157870	FAM213B	3819	5.19E-304	6.05E-302	0.0085063	0.9913370	0.0001567	0.9914937	0.0001580	1.00E-05
liberal	PD_HY3	eQTLGen	ENSG00000215914	MMP23A	3065	4.37E-15	5.32E-13	0.0081380	0.9917074	0.0001546	0.9918620	0.0001559	1.00E-05
liberal	PD_HY3	eQTLGen	ENSG00000242485	MRPL20	2492	2.62E-94	3.27E-92	0.0079474	0.9918983	0.0001543	0.9920526	0.0001556	1.00E-05
liberal	PD_HY3	psychencode	ENSG00000149527	PLCH2	2038	7.98E-09	9.01E-07	0.0087877	0.9910576	0.0001537	0.9912114	0.0001551	1.00E-05
liberal	PD_HY3	eQTLGen	ENSG00000157911	PEX10	3799	2.02E-37	2.34E-35	0.0085534	0.9912959	0.0001507	0.9914466	0.0001520	1.00E-05
liberal	PD_HY3	psychencode	ENSG00000272420		1965	6.97E-19	7.88E-17	0.0087592	0.9910902	0.0001506	0.9912408	0.0001519	1.00E-05
liberal	PD_HY3	eQTLGen	ENSG00000067606	PRKCZ	3665	1.93E-51	2.27E-49	0.0084607	0.9913888	0.0001505	0.9915393	0.0001519	1.00E-05
liberal	PD_HY3	eQTLGen	ENSG00000238164		3780	1.29E-18	1.51E-16	0.0085196	0.9913301	0.0001503	0.9914804	0.0001516	1.00E-05
liberal	PD_HY3	psychencode	ENSG00000269896		1958	2.44E-31	2.76E-29	0.0087574	0.9910958	0.0001469	0.9912426	0.0001482	1.00E-05
liberal	PD_HY3	eQTLGen	ENSG00000269896		3735	9.82E-101	1.14E-98	0.0085255	0.9913305	0.0001440	0.9914745	0.0001453	1.00E-05
robust	PD_HY3	psychencode	ENSG00000215912	TTC34	2207	8.58E-06	0.000973319	0.0087305	0.9901439	0.0001437	0.9902876	0.0001451	5.00E-06
liberal	PD_HY3	eQTLGen	ENSG00000116151	MORN1	3746	5.20E-69	6.05E-67	0.0085271	0.9913294	0.0001434	0.9914729	0.0001447	1.00E-05
liberal	PD_HY3	eQTLGen	ENSG00000157873	TNFRSF14	3769	5.38E-306	6.26E-304	0.0085144	0.9913427	0.0001429	0.9914856	0.0001442	1.00E-05
robust	PD_HY3	eQTLGen	ENSG00000189339	SLC35E2B	3082	1.38E-65	1.69E-63	0.0081384	0.9917267	0.0001350	0.9918616	0.0001361	5.00E-06
robust	PD_HY3	eQTLGen	ENSG00000227775		3093	2.81E-21	3.43E-19	0.0081361	0.9917355	0.0001284	0.9918639	0.0001295	5.00E-06
robust	PD_HY3	psychencode	ENSG00000215915	ATAD3C	969	8.59E-08	1.01E-05	0.0084394	0.9914261	0.0001243	0.9915504	0.0001254	5.00E-06
robust	PD_HY3	psychencode	ENSG00000215790	SLC35E2	1248	1.60E-36	1.85E-34	0.0085340	0.9913525	0.0001135	0.9914660	0.0001145	5.00E-06
robust	PD_HY3	eQTLGen	ENSG00000142606	MMEL1	3865	8.57E-237	9.97E-235	0.0085195	0.9913671	0.0001134	0.9914805	0.0001143	5.00E-06
robust	PD_HY3	psychencode	ENSG00000160075	SSU72	1141	6.89E-17	8.06E-15	0.0084793	0.9914077	0.0001129	0.9915207	0.0001139	5.00E-06
robust	PD_HY3	eQTLGen	ENSG00000178642		3732	5.35E-52	6.21E-50	0.0085441	0.9913441	0.0001118	0.9914559	0.0001128	5.00E-06
robust	PD_HY3	psychencode	ENSG00000116151	MORN1	2025	4.97E-07	5.60E-05	0.0087859	0.9910506	0.0001070	0.9911576	0.0001080	5.00E-06
robust	PD_HY3	psychencode	ENSG00000157870	FAM213B	2147	3.33E-07	3.77E-05	0.0087476	0.9911081	0.0001062	0.9912144	0.0001072	5.00E-06
robust	PD_HY3	eQTLGen	ENSG00000160075	SSU72	2871	5.40E-306	6.66E-304	0.0080519	0.9918419	0.0001062	0.9919481	0.0001071	5.00E-06
robust	PD_HY3	psychencode	ENSG00000162585	C1orf86	1927	5.55E-15	6.28E-13	0.0087489	0.9911485	0.0001026	0.9912511	0.0001035	5.00E-06
robust	PD_HY3	eQTLGen	ENSG00000149527	PLCH2	3746	8.72E-305	1.01E-302	0.0085430	0.9913552	0.0001018	0.9914570	0.0001027	5.00E-06
robust	PD_HY3	eQTLGen	ENSG00000169885	CALML6	3417	2.43E-29	2.90E-27	0.0082872	0.9916117	0.0001010	0.9917128	0.0001019	5.00E-06

robust	PD_HY3	eQTLGen	ENSG00000197785	ATAD3A	2793	3.91E-39	4.83E-37	0.0080231	0.9918802	0.0000967	0.9919769	0.0000975	5.00E-06
robust	PD_HY3	eQTLGen	ENSG00000187730	GABRD	3568	1.29E-29	1.52E-27	0.0083868	0.9915165	0.0000967	0.9916132	0.0000975	5.00E-06
robust	PD_HY3	eQTLGen	ENSG00000224387		3723	4.65E-29	5.40E-27	0.0085361	0.9913678	0.0000961	0.9914639	0.0000969	5.00E-06
robust	PD_HY3	eQTLGen	ENSG00000228594	C1orf233	2982	2.19E-08	2.68E-06	0.0081011	0.9918017	0.0000946	0.9918962	0.0000954	5.00E-06
robust	PD_HY3	eQTLGen	ENSG00000157916	RER1	3807	2.21E-21	2.56E-19	0.0085533	0.9913524	0.0000943	0.9914467	0.0000951	5.00E-06
robust	PD_HY3	eQTLGen	ENSG00000215014		2882	1.86E-103	2.29E-101	0.0080680	0.9918392	0.0000929	0.9919320	0.0000936	5.00E-06
robust	PD_HY3	eQTLGen	ENSG00000269554		3729	1.06E-08	1.23E-06	0.0085100	0.9913962	0.0000925	0.9914887	0.0000933	5.00E-06
robust	PD_HY3	eQTLGen	ENSG00000162585	C1orf86	3755	1.28E-299	1.49E-297	0.0085102	0.9913975	0.0000922	0.9914898	0.0000930	5.00E-06
robust	PD_HY3	eQTLGen	ENSG00000229393		3754	5.27E-18	6.12E-16	0.0085342	0.9913769	0.0000889	0.9914658	0.0000897	5.00E-06
robust	PD_HY3	psychencode	ENSG00000157911	PEX10	2050	2.05E-10	2.31E-08	0.0087901	0.9911242	0.0000857	0.9912099	0.0000865	5.00E-06
robust	PD_HY3	eQTLGen	ENSG00000157881	PANK4	3801	1.21E-86	1.41E-84	0.0085283	0.9913900	0.0000817	0.9914717	0.0000824	5.00E-06
robust	PD_HY3	eQTLGen	ENSG00000182873		3731	4.17E-79	4.86E-77	0.0085097	0.9914103	0.0000800	0.9914903	0.0000806	5.00E-06
robust	PD_HY3	eQTLGen	ENSG00000157870	FAM213B	3819	5.19E-304	6.05E-302	0.0085070	0.9914147	0.0000783	0.9914930	0.0000790	5.00E-06
robust	PD_HY3	eQTLGen	ENSG00000215914	MMP23A	3065	4.37E-15	5.32E-13	0.0081386	0.9917841	0.0000773	0.9918614	0.0000780	5.00E-06
robust	PD_HY3	eQTLGen	ENSG00000242485	MRPL20	2492	2.62E-94	3.27E-92	0.0079480	0.9919748	0.0000772	0.9920520	0.0000778	5.00E-06
robust	PD_HY3	psychencode	ENSG00000149527	PLCH2	2038	7.99E-09	9.01E-07	0.0087884	0.9911338	0.0000769	0.9912107	0.0000776	5.00E-06
robust	PD_HY3	eQTLGen	ENSG00000157911	PEX10	3799	2.02E-37	2.34E-35	0.0085541	0.9913706	0.0000754	0.9914459	0.0000760	5.00E-06
robust	PD_HY3	psychencode	ENSG00000272420		1965	6.97E-19	7.88E-17	0.0087598	0.9911649	0.0000753	0.9912402	0.0000760	5.00E-06
robust	PD_HY3	eQTLGen	ENSG00000067606	PRKCZ	3665	1.93E-51	2.27E-49	0.0084613	0.9914634	0.0000753	0.9915387	0.0000759	5.00E-06
robust	PD_HY3	eQTLGen	ENSG00000238164		3780	1.29E-18	1.51E-16	0.0085203	0.9914046	0.0000752	0.9914797	0.0000758	5.00E-06
robust	PD_HY3	psychencode	ENSG00000269896		1958	2.44E-31	2.76E-29	0.0087580	0.9911685	0.0000734	0.9912420	0.0000741	5.00E-06
robust	PD_HY3	eQTLGen	ENSG00000269896		3735	9.82E-101	1.14E-98	0.0085261	0.9914019	0.0000720	0.9914739	0.0000726	5.00E-06
robust	PD_HY3	eQTLGen	ENSG00000116151	MORN1	3746	5.20E-69	6.05E-67	0.0085277	0.9914005	0.0000717	0.9914723	0.0000723	5.00E-06
robust	PD_HY3	eQTLGen	ENSG00000157873	TNFRSF14	3769	5.38E-306	6.26E-304	0.0085150	0.9914135	0.0000715	0.9914850	0.0000721	5.00E-06

Supplementary Table 4. Results for PD risk SNPs from Nalls 2019

							mortality		HY3	
**nearest gene**	**rsID**	**chr**	**bp**	**effect allele**	**other allele**	**effect allele freq**	**beta**	**p**	**beta**	**p**
PMVK	rs114138760	1	154898185	C	G	0.014	0.094	0.618	0.180	0.455
KRTCAP2	rs35749011	1	155135036	A	G	0.028	0.395	3.59E-04	0.120	0.459
FCGR2A	rs6658353	1	161469054	C	G	0.534	0.034	0.330	-0.036	0.495
VAMP4	rs11578699	1	171719769	T	C	0.192	-0.051	0.255	0.087	0.185
NUCKS1	rs823118	1	205723572	T	C	0.558	-0.019	0.585	0.035	0.523
RAB29	rs11557080	1	205737739	A	G	0.129	-0.094	0.076	0.175	0.017
ITPKB	rs4653767	1	226916078	T	C	0.733	-0.025	0.536	0.044	0.468
SIPA1L2	rs10797576	1	232664611	T	C	0.138	0.022	0.665	-0.029	0.713
KCNS3	rs76116224	2	18147848	A	T	0.908	-0.007	0.906	-0.048	0.596
KCNIP3	rs2042477	2	96000943	A	T	0.245	0.013	0.742	-0.042	0.499
MAP4K4	rs11683001	2	102396963	A	T	0.343	-0.058	0.119	0.015	0.785
TMEM163	rs57891859	2	135464616	A	G	0.757	0.019	0.660	-0.127	0.037
STK39	rs1474055	2	169110394	T	C	0.142	-0.030	0.536	0.040	0.586
SATB1	rs73038319	3	18361759	A	C	0.955	-0.141	0.090	0.060	0.666
LINC00693	rs6808178	3	28705690	T	C	0.404	-0.064	0.080	-0.069	0.209
IP6K2	rs12497850	3	48748989	T	G	0.650	-0.023	0.535	0.014	0.803
KPNA1	rs55961674	3	122196892	T	C	0.155	-0.002	0.965	0.095	0.174
MED12L	rs11707416	3	151108965	A	T	0.368	-0.051	0.157	0.030	0.588
SPTSSB	rs1450522	3	161077630	A	G	0.679	0.014	0.699	0.047	0.407
MCCC1	rs10513789	3	182760073	T	G	0.828	0.005	0.923	-0.007	0.919
GAK	rs873786	4	925376	T	C	0.096	-0.033	0.590	0.039	0.657
TMEM175	rs34311866	4	951947	T	C	0.796	-0.105	0.014	-0.049	0.440
BST1	rs4698412	4	15737348	A	G	0.570	-0.018	0.615	-0.087	0.102
LCORL	rs34025766	4	17968811	A	T	0.142	0.080	0.110	0.079	0.281
SCARB2	rs6825004	4	77110365	C	G	0.712	0.090	0.021	0.016	0.784
FAM47E	rs4101061	4	77147969	A	G	0.701	-0.016	0.666	-0.135	0.018
FAM47E-STBD1	rs6854006	4	77198054	T	C	0.350	-0.026	0.464	0.025	0.641
SNCA	rs356182	4	90626111	A	G	0.597	0.015	0.667	-0.006	0.910
SNCA	rs5019538	4	90636630	A	G	0.671	0.024	0.511	0.014	0.807
CAMK2D	rs13117519	4	114369065	T	C	0.188	0.005	0.916	-0.034	0.615
CLCN3	rs62333164	4	170583157	A	G	0.317	0.015	0.679	-0.165	0.005
ELOVL7	rs1867598	5	60137959	A	G	0.903	-0.071	0.230	0.085	0.385
PAM	rs26431	5	102365794	C	G	0.703	-0.042	0.265	0.032	0.584

C5orf24	rs11950533	5	134199105	A	C	0.099	0.088	0.132	0.062	0.477
LOC100131289	rs4140646	6	27738801	A	G	0.252	-0.080	0.113	-0.076	0.325
TRIM40	rs9261484	6	30108683	T	C	0.254	0.058	0.222	0.029	0.698
HLA-DRB5	rs112485576	6	32578772	A	C	0.171	0.061	0.267	-0.035	0.709
RIMS1	rs12528068	6	72487762	T	C	0.298	0.063	0.111	0.033	0.572
FYN	rs997368	6	112243291	A	G	0.822	0.001	0.986	0.055	0.449
RPS12	rs75859381	6	133210361	T	C	0.965	-0.157	0.090	-0.242	0.092
GPNMB	rs199351	7	23300049	A	C	0.617	-0.024	0.501	0.017	0.752
GS1-124K5.11	rs76949143	7	66009851	A	T	0.054	-0.080	0.330	0.040	0.751
CTSB	rs1293298	8	11712443	A	C	0.754	-0.002	0.968	0.003	0.965
FGF20	rs620513	8	16697593	T	G	0.261	0.018	0.638	0.091	0.127
BIN3	rs2280104	8	22525980	T	C	0.368	-0.018	0.622	-0.039	0.475
FAM49B	rs2086641	8	130901909	T	C	0.731	-0.022	0.571	0.074	0.220
SH3GL2	rs13294100	9	17579690	T	G	0.338	0.023	0.538	-0.024	0.673
SH3GL2	rs10756907	9	17727065	A	G	0.755	-0.016	0.701	-0.010	0.872
UBAP2	rs6476434	9	34046391	T	C	0.729	-0.005	0.891	0.037	0.536
ITGA8	rs896435	10	15557406	T	C	0.691	-0.023	0.538	0.035	0.555
GBF1	rs10748818	10	104015279	A	G	0.839	-0.014	0.772	-0.016	0.826
BAG3	rs72840788	10	121415685	A	G	0.228	-0.068	0.103	-0.052	0.419
INPP5F	rs117896735	10	121536327	A	G	0.028	0.230	0.030	0.058	0.725
RNF141	rs7938782	11	10558777	A	G	0.895	0.041	0.474	0.045	0.609
DLG2	rs12283611	11	83487277	A	C	0.386	0.062	0.081	0.025	0.638
IGSF9B	rs3802920	11	133787001	T	G	0.203	-0.016	0.721	0.106	0.092
LRRK2	rs76904798	12	40614434	T	C	0.158	-0.105	0.032	-0.051	0.483
SCAF11	rs7134559	12	46419086	T	C	0.378	0.065	0.073	0.057	0.294
HIP1R	rs10847864	12	123326598	T	G	0.378	-0.026	0.472	-0.034	0.574
FBRSL1	rs11610045	12	133063768	A	G	0.513	-0.007	0.839	0.013	0.810
CAB39L	rs9568188	13	49927732	T	C	0.727	0.051	0.187	0.036	0.547
MBNL2	rs4771268	13	97865021	T	C	0.236	0.067	0.098	-0.027	0.672
MIPOL1	rs12147950	14	37989270	T	C	0.418	0.054	0.129	-0.115	0.037
GCH1	rs11158026	14	55348869	T	C	0.303	-0.043	0.255	0.036	0.522
RPS6KL1	rs3742785	14	75373034	A	C	0.781	0.052	0.231	0.045	0.491
GALC	rs979812	14	88464264	T	G	0.440	0.003	0.924	-0.002	0.977
VPS13C	rs2251086	15	61997385	T	C	0.130	0.106	0.037	0.040	0.619
SYT17	rs6497339	16	19277493	A	T	0.462	-0.026	0.501	-0.004	0.941
CD19	rs2904880	16	28944396	C	G	0.319	0.032	0.394	0.020	0.732
SETD1A	rs11150601	16	30977799	A	G	0.647	0.049	0.179	-0.027	0.622
NOD2	rs6500328	16	50736656	A	G	0.610	-0.003	0.938	0.045	0.406
CASC16	rs3104783	16	52636242	A	C	0.417	-0.058	0.105	-0.046	0.389
CHD9	rs10221156	16	52969426	A	G	0.087	0.064	0.305	-0.268	0.010
CHRNB1	rs12600861	17	7355621	A	C	0.622	0.029	0.429	0.049	0.378
RETREG3	rs12951632	17	40741013	T	C	0.735	0.022	0.576	-0.087	0.132
UBTF	rs2269906	17	42294337	A	C	0.675	-0.002	0.959	-0.039	0.483
FAM171A2	rs850738	17	42434630	A	G	0.577	-0.022	0.539	-0.020	0.711

CRHR1	rs62053943	17	43744203	T	C	0.131	-0.027	0.587	-0.019	0.813
CRHR1	rs117615688	17	43798308	A	G	0.058	0.059	0.577	-0.022	0.860
WNT3	rs11658976	17	44866805	A	G	0.587	0.005	0.900	-0.032	0.547
BRIP1	rs61169879	17	59917366	T	C	0.161	-0.039	0.405	0.080	0.246
DNAH17	rs666463	17	76425480	A	T	0.832	0.004	0.933	0.052	0.459
ASXL3	rs1941685	18	31304318	T	G	0.504	-0.039	0.259	-0.099	0.060
RIT2	rs12456492	18	40673380	A	G	0.662	0.036	0.331	-0.042	0.453
MEX3C	rs8087969	18	48683589	T	G	0.525	-0.013	0.705	0.053	0.316
SPPL2B	rs55818311	19	2341047	T	C	0.665	0.023	0.532	0.048	0.385
CRLS1	rs77351827	20	6006041	T	C	0.125	-0.099	0.081	-0.042	0.608
DYRK1A	rs2248244	21	38852361	A	G	0.289	-0.018	0.640	0.038	0.510

Supplementary Table 5. Results for candidate variant analysis.

								mortality		HY3	
**Previous study**	**nearest gene**	**rsID**	**chr**	**bp**	**effect allele**	**other allele**	**effect allele freq**	**beta**	**p-value**	**beta**	**p-value**
Iwaki2019 - PD progression to HY3	*SLC44A1*	rs382940	9	108058562	A	T	0.074	0.098	0.141	-0.009	0.925
Liu2021 - PD progression to dementia	*RIMS2*	rs182987047	8	105249272	T	A	0.018	-0.108	0.485	0.016	0.943
Liu2021 - PD progression to dementia	*TMEM108*	rs138073281	3	132985956	C	A	0.018	0.085	0.591	0.084	0.687
Liu2021 - PD progression to dementia	*WWOX*	rs8050111	16	78281160	G	A	0.065	-0.017	0.814	0.005	0.963
PD cognition and dementia, APOE ε2	*APOE*	rs7412	19	45412079	T	C	0.080	-0.091	0.180	0.102	0.280
PD cognition and dementia, MAPT H1	*MAPT*	rs8070723	17	44081064	G	A	0.183	-0.010	0.828	-0.037	0.586
Jabbari2021 - PSP mortality	*SLC2A13*	rs2242367	12	40413698	A	G	0.265	0.118	0.002	-0.011	0.861

HY3 = Hoehn and Yahr stage 3 or greater; PD = Parkinson's disease; PSP = Progressive Supranuclear Palsy

Supplementary Table 6. Top mortality SNPs and their association with progression to H&Y3

							mortality			HY3		
**chr**	**bp**	**rsID**	**effect allele**	**non-effect allele**	**effect allele freq**	**nearest gene**	**beta**	**SE**	**p-value**	**beta**	**SE**	**p-value**
19	45411941	rs429358	C	T	0.160	*APOE*	0.295	0.046	1.35E-10	0.156	0.075	0.037
7	139637422	rs4726467	T	C	0.021	*TBXAS1*	0.713	0.116	7.71E-10	-0.248	0.266	0.352
12	33635494	rs10437796	A	C	0.098	*SYT10*	0.304	0.056	5.31E-08	0.022	0.090	0.807
9	17616880	rs3808753	G	A	0.035	*SH3GL2*	0.448	0.088	3.34E-07	0.156	0.166	0.347
3	113979619	rs142285045	A	C	0.013	*ZNF80*	0.836	0.165	3.80E-07	0.012	0.275	0.966
18	33965217	rs76125680	C	G	0.012	*FHOD3*	0.874	0.174	5.32E-07	-15.752	2601.490	0.995*
15	34896795	rs35294489	G	A	0.014	*GOLGA8B*	0.757	0.152	6.07E-07	-0.110	0.243	0.649
5	55533638	rs28811891	T	C	0.026	*ANKRD55*	0.544	0.109	6.21E-07	0.181	0.162	0.263
21	31807104	rs145506557	C	A	0.016	*KRTAP13-4*	0.896	0.180	6.59E-07	0.561	0.439	0.202*
10	98786658	rs17112311	A	G	0.049	*C10orf12*	0.364	0.074	8.07E-07	0.024	0.124	0.844

Genome coordinates are in build hg19/GRCh37.

* These SNPs were genotyped in < 1000 individuals across all cohorts in the H&Y3 GWAS. All other SNPs were genotyped in at least 1000 individuals.

Abbreviations: BP = base pair, chr = chromosome, CI = Confidence Interval, freq = frequency, GCTA-COJO = Genome-wide Complex Trait Analysis conditional and joint analysis, SE = Standard Error, SNP = Single Nucleotide Polymorphism

Supplementary Table 7. Mortality GWAS - Cox proportional hazards assumption p-value for each of the top 10 SNPs for each cohort included in the meta-analysis

chr	bp	rsid	Tracking Parkinson's	Oxford Discovery	QSBB	UKB incident	UKB prevalent	Calypso	CamPaIGN	DIGPD	Trondheim	Oslo
19	45411941	rs429358	0.726	0.437	0.074	0.710	0.657	0.250	0.522	0.237	0.009	0.720
7	139637422	rs4726467	0.587	0.703	0.941	0.083	0.042	0.166	0.115	0.362	NA	0.974
12	33635494	rs10437796	0.549	0.005	0.193	0.800	0.912	0.734	0.733	0.799	0.621	0.475
9	17616880	rs3808753	0.138	0.689	0.519	0.875	0.393	0.071	0.459	0.543	0.698	0.992
3	113979619	rs142285045	0.889	0.082	NA	0.637	0.716	0.320	NA	0.380	NA	NA
18	33965217	rs76125680	NA	NA	NA	0.031	0.027	0.541	NA	0.143	0.354	NA
15	34896795	rs35294489	0.543	0.242	NA	0.327	0.304	NA	1.000	NA	NA	0.644
5	55533638	rs28811891	0.531	0.001	NA	0.401	0.385	0.076	0.956	0.216	0.586	0.095
21	31807104	rs145506557	NA	NA	NA	0.078	0.568	NA	NA	NA	NA	NA
10	98786658	rs17112311	0.449	0.111	0.646	0.079	0.122	0.209	0.045	0.299	0.007	0.311

P-values for the test of the proportional hazards assumption generated using the cox.zph function in R. P-values less than 0.05 indicate the proportional hazards assumption was not met. We did not filter out SNPs or cohorts based on the proportional hazards assumption p-value, as the assumption was met in the majority of cohorts for the top SNPs.

Supplementary Table 8. H&Y3+ GWAS - Cox proportional hazards assumption p-value for each of the top 11 SNPs for each cohort included in the meta-analysis.

chr	bp	rsid	Tracking Parkinson's	Oxford Discovery	PPMI	DIGPD	Oslo
1	2315032	rs115217673	0.756	0.314	0.171	NA	0.245
7	97470925	rs145274312	NA	0.787	NA	0.072	NA
4	120566153	rs113120976	NA	0.646	NA	0.007	NA
2	61742356	rs141421624	0.688	0.263	0.171	0.435	0.335
1	231267735	rs74796254	0.614	0.532	0.239	0.809	0.753
1	247425485	rs72771919	0.450	0.181	0.867	0.063	0.853
4	186697549	rs75614365	0.484	0.974	0.867	0.918	0.508
1	43105085	rs75140767	0.473	0.079	1.000	0.744	0.729
2	131557187	rs7566590	0.357	0.620	0.669	0.996	0.288
6	11284774	rs148949229	NA	0.744	0.916	1.000	0.004
15	82198898	rs58698247	0.700	0.031	0.521	0.140	0.010

P-values for the test of the proportional hazards assumption generated using the cox.zph function in R. P-values less than 0.05 indicate the proportional hazards assumption was not met. We did not filter out SNPs or cohorts based on the proportional hazards assumption p-value, as the assumption was met in the majority of cohorts for the top SNPs

Supplementary Table 9. PAINTOR results for mortality GWAS, variants with posterior probability > 0.9.

chr	bp	A1 (effect)	A0 (noneffect)	ZSCORE	posterior_probability
3	113979619	A	C	-5.078663122	0.999
5	55533638	T	C	-4.984725	0.999
21	31807104	A	C	-4.9729549	0.999

Supplementary Table 10. PAINTOR results for H&Y3+ GWAS, variants with posterior probability > 0.9.

chr	bp	A1 (effect)	A0 (noneffect)	ZSCORE	posterior_probability
1	43105085	A	G	-5.1079342	0.997
1	231267735	T	C	-5.2989038	0.999
1	247425485	A	G	-5.2598155	1.000
2	61742356	A	G	-5.5367704	1.000
4	120566153	T	C	-5.7921042	1.000

Supplementary Table 11. SlopeHunter top SNPs by adjusted pvalue.

					Association with PD risk			Association with PD mortality					
								Unadjusted			Adjusted using Slope-Hunter		
SNP	CHR	BP	nearest_gene	Distance to gene	beta	SE	pval	beta	SE	pval	beta	SE	pval
19:45388130	19	45388130	*TOMM40*	6,347	-0.034	0.025	0.168	0.232	0.047	9.54E-07	0.243	0.048	4.91E-07
13:31540660	13	31540660	*TEX26*	0	-0.128	0.088	0.145	0.579	0.124	3.20E-06	0.619	0.129	1.53E-06
6:96387321	6	96387321	*FUT9*	76,524	-0.104	0.135	0.438	1.061	0.224	2.25E-06	1.094	0.229	1.84E-06
12:33573752	12	33573752	*SYT10*	0	0.020	0.031	0.506	0.275	0.056	6.97E-07	0.269	0.057	1.97E-06
5:55533638	5	55533638	*ANKRD55*	4,452	0.058	0.070	0.410	0.544	0.109	6.16E-07	0.525	0.112	2.68E-06
6:17028264	6	17028264	*STMND1*	74,225	-0.056	0.098	0.570	0.649	0.138	2.65E-06	0.666	0.142	2.85E-06
1:74507315	1	74507315	*LRRIQ3*	0	-0.047	0.043	0.281	0.382	0.083	4.60E-06	0.397	0.085	2.93E-06
13:72433394	13	72433394	*DACH1*	0	0.016	0.090	0.863	0.567	0.120	2.40E-06	0.562	0.124	6.00E-06
2:166279387	2	166279387	*CSRNP3*	46,770	0.162	0.089	0.067	-0.607	0.141	1.68E-05	-0.659	0.146	6.19E-06
11:77522336	11	77522336	*AAMDC*	9,811	0.081	0.088	0.353	-0.593	0.135	1.03E-05	-0.619	0.138	7.47E-06

Supplementary Table 12. Top mortality SNPs without adjusting for age at onset, and their direct association with age at onset in a linear model

					Association with mortality without adjusting for age at onset			Association with age at onset		
**chr**	**bp**	**rsID**	**effect allele**	**non-effect allele**	**beta**	**SE**	**pvalue**	**beta**	**SE**	**pvalue**
19	45411941	rs429358	C	T	0.239	0.044	4.80E-08	0.054	0.206	0.794
7	139637422	rs4726467	T	C	0.590	0.109	5.62E-08	-0.961	0.541	0.076
12	33635494	rs10437796	A	C	0.227	0.054	2.17E-05	-0.316	0.248	0.202
9	17616880	rs3808753	G	A	0.343	0.084	4.68E-05	-0.148	0.429	0.730
3	113979619	rs142285045	A	C	0.681	0.157	1.53E-05	-0.186	0.725	0.797
18	33965217	rs76125680	C	G	0.871	0.170	2.86E-07	0.615	0.881	0.485
15	34896795	rs35294489	G	A	0.697	0.151	4.06E-06	-0.136	0.657	0.836
5	55533638	rs28811891	T	C	0.328	0.104	0.002	-0.055	0.472	0.907
21	31807104	rs145506557	C	A	0.775	0.173	7.32E-06	0.389	0.767	0.612
10	98786658	rs17112311	A	G	0.250	0.071	3.96E-04	-0.096	0.347	0.782

Supplementary Table 13. Top 4 SNPs from the H&Y3+ GWAS and their association with progression to H&Y4+ and H&Y5

							H&Y4+			H&Y5		
**chr**	**bp**	**rsID**	**effect allele**	**non-effect allele**	**effect allele freq**	**nearest gene**	**beta**	**SE**	**p-value**	**beta**	**SE**	**p-value**
1	2315032	rs115217673	A	G	0.016	*MORN1*	0.291	0.387	0.453	0.390	1.025	0.704
7	97470925	rs145274312	A	G	0.011	*ASNS*	1.883	1.129	0.096	5.636	0.006	2.045
4	120566153	rs113120976	T	C	0.013	*PDE5A*	0.201	1.025	0.845	-17.283	10768.670	0.999
2	61742356	rs141421624	G	A	0.017	*XPO1*	0.680	0.357	0.057	0.794	1.079	0.462

SUPPLEMENTARY DISCUSSION

Potential causal genes

Here we briefly discuss the loci nominated by our GWASs. For ease, these have been named after the physically closest genes but we discuss other possible causal genes at the locus.

TBXAS1: TBXAS1 encodes Thromboxane A Synthase 1, which catalyses the conversion of prostaglandin H2 to thromboxane A2, which acts as a platelet aggregator and vasoconstrictor. Variants in *TBXAS1* have previously been associated with coronary artery disease in a number of GWASs.[5–8] We showed that a locus in *TBXAS1* was associated with all-cause mortality in PD cohorts. Thus, it is possible that the association between *TBXAS1* and PD mortality may be due to increased coronary artery disease or other non-PD causes.

However, there are a few important factors to consider. Firstly, the locus we identified for mortality in PD cohorts is different from those associated with coronary artery disease, so it may be that different SNPs within this gene have different effects. Secondly, our search of the most recent longevity GWASs[9] suggests that variation at *TBXAS1* does not influence mortality and longevity in the general population. Thirdly, it is also possible that patients with PD are more susceptible to adverse effects of a SNP than healthy controls, so PD or its treatment increases vulnerability to the SNP effect on coronary artery disease but the cause of death is not able to be classified as 'PD-related'. This has also been seen in previous non-genetic studies of PD mortality.[10] Thus future analyses including cause of death are needed to clarify the mechanism through which *TBXAS1* variants influence mortality in PD patients.

SYT10: We also found evidence that a locus near SYT10 (synaptotagmin 10) in Chromosome 12 was associated with mortality. This protein is a calcium sensor involved in regulation of calcium-dependent exocytosis, specifically related to peptidergic vesicles.[11] Deletion of Syt10 impairs IGF-1 secretion in olfactory bulb neurons[11], and IGF-1 deficiency has been suggested to play a role in the development of PD[12] and deficits in dopamine neuron firing accompanied by motor deficits[13]. This locus was also nominally associated with longevity in the general population (beta=-0.008, p=0.003)[9].

MORN1: MORN1 encodes the Membrane Occupation and Recognition (MORN) repeat containing 1 protein. The function of this protein is not very well understood or studied in humans.

ASNS: This gene encodes Asparagine synthetase which converts aspartate and glutamine to asparagine and glutamate[14]. Glutamate is an important neurotransmitter and extracellular glutamate can be neurotoxic through activation of N-methyl-D-aspartate (NMDA) receptors.[15] This excitotoxicity may contribute to neuronal death in neurodegenerative diseases including PD and AD.[15,16] It has also been shown that asparaginase, a cancer therapy which breaks down asparagine, has a neuroprotective effect in a SNCA A53T cell model of PD by depleting glutamine and activating the degradation of α-synuclein through autophagy and improved mitochondrial function.[17]

We also note that for two of the loci (near *PDE5A* and *ASNS*), there were no nearby SNPs with similar effect sizes providing the supportive GWAS 'peak', however this is likely due to a lack of SNPs in linkage disequilibrium with the lead SNP as shown in the LocusZoom plots (Supplementary Figures 8-9) and LDproxy (https://ldlink.nih.gov). The summary statistics from this study are publicly available and replication of these hits is needed.

PDE5A: Phosphoeidasterase 5A (PDE5A) is a member of the cyclic nucleotide phosphodiesterase (PDE) family which are responsible for breaking down the cyclic nucleotides: cyclic adenosine monophosphate (cAMP) and cyclic guanosine monophosphate (cGMP). These intracellular second messengers are important in aging, AD, Huntington's disease and PD.[18] PDE5 specifically breaks down cGMP. Studies have shown that PDE inhibitors like zaprinast restored long-term depression (a form of synaptic plasticity) in mice overexpressing SNCA A53T[19], and reduced the severity of dyskinesias and increased cAMP and cGMP levels in a hemiparkinsonian rat model.[20] However PDE5 is not very highly expressed in the striatum compared to other PDEs and also relative to other tissues, and other PDEs may be important in PD.[18,21]

The top SNP in this locus was also a significant eQTL for *USP53* in blood. *USP53* has been linked to hepatocellular carcinoma and oesophageal cancer, where its downregulation was found in tumour tissues from patients.[22–24] Overexpression of USP53 promoted tumour cell death and reduced tumour growth, suggesting an anti-tumour role in certain cancers.[23,24]

XPO1: This locus spanned 4 genes including *XPO1*, *USP34*, *C2orf74* and *KIAA1841*, with the lead SNP influencing expression of *KIAA1841* and *AHSA2* in blood and *C2orf74* in brain cortex. XPO1 (Exportin 1) transports tumour suppressors, anti-inflammatory factors and growth regulating proteins from the nucleus to the cytoplasm.[25,26] In a PD mouse model, inhibition of XPO1 with eltanexor prevented dopaminergic neuron loss and microglial activation, and rescued locomotor incoordination.[27]

USP34: USP34 is a ubiquitin-specific protease that modulates the Wnt signalling pathway by removing ubiquitin conjugates from axin and controlling the level of axin.[28]

C2orf74, chromosome 2 open reading frame 74, has not been extensively studied. It may be involved in autoimmune disorders, where differences in gene expression have been found in celiac disease,[29] and GWAS hits within C2orf74 for celiac disease, ulcerative colitis, and Crohn's disease may alter gene expression in thymic tissue.[30] However, so far it has not been studied in PD.

SUPPLEMENTARY NOTES

Software

METAL (version 2011-03-25): http://www.sph.umich.edu/csg/abecasis/metal/ (RRID:SCR_002013).
R (v3·6) https://cran.r-project.org/. R Project for Statistical Computing (RRID:SCR_001905).
PLINK (v1.9): https://www.cog-genomics.org/plink/ PLINK (RRID:SCR_001757).
LocusZoom: http://locuszoom.org/ (RRID:SCR_009257)
liftOver: https://genome.sph.umich.edu/wiki/LiftOver (RRID:SCR_018160).
GCTA-COJO (v1.94.1): https://yanglab.westlake.edu.cn/software/gcta/#COJO
MAGMA: https://snp-magma.sourceforge.net (RRID:SCR_005757)
PAINTOR (v3.1): https://bogdan.dgsom.ucla.edu/pages/paintor/
FUMA (v1.5.1): https://fuma.ctglab.nl/ (RRID:SCR_017521)

R packages for specific analyses:

- *survSNP:* https://cran.r-project.org/web/packages/survSNP/index.html
- *coloc* (v5.1): https://cran.r-project.org/web/packages/coloc/, https://chr1swallace.github.io/coloc/index.html
- *colochelpR:* https://github.com/RHReynolds/colochelpR
- *forestplot* (v3.3.1)*:* https://cran.r-project.org/web/packages/forestplot/
- *survival* (RRID:SCR_021137)*:* https://cran.r-project.org/web/packages/survival/index.html
- *meta* (RRID:SCR_019055)*:* https://cran.r-project.org/web/packages/meta/index.html
- *hudson* (v1.0.0): https://github.com/anastasia-lucas/hudson

Public databases

GWAS Catalogue: https://www.ebi.ac.uk/gwas

LDpair, LDproxy (version 5.6.2): https://ldlink.nih.gov/
eQTLGen: https://www.eqtlgen.org/
MetaBrain: https://www.metabrain.nl/
GTEx eQTL Browser (RRID:SCR_001618): https://gtexportal.org/
PsychENCODE (RRID:SCR_021760): http://resource.psychencode.org/
Michigan Imputation Server (RRID:SCR_017579):
https://imputationserver.readthedocs.io/en/latest/
HapMap 3 reference panel:
https://www.sanger.ac.uk/resources/downloads/human/hapmap3.html/
https://ftp.ncbi.nlm.nih.gov/hapmap/
Iwaki 2019 Progression GWAS summary statistics:
https://pdgenetics.shinyapps.io/pdprogmetagwasbrowser/
Timmers 2020 longevity GWAS: www.longevitygenomics.org/downloads

DIGPD study group

Steering committee: Jean-Christophe Corvol, MD (Pitié-Salpêtrière Hospital, Paris, principal investigator of DIGPD); Alexis Elbaz, MD (CESP, Villejuif, member of the steering committee and PI for statistical analyses), Marie Vidailhet, MD (Pitié-Salpêtrière Hospital, Paris, member of the steering committee), Alexis Brice, MD (Pitié-Salpêtrière Hospital, Paris, member of the steering committee and PI for genetic analysis) ; Statistical analyses: Fanny Artaud, PhD (CESP, Villejuif, statistician); Principal investigators for sites: Frédéric Bourdain, MD (CH Foch, Suresnes, PI for site), Jean-Philippe Brandel, MD (Fondation Rothschild, Paris, PI for site), Pascal Derkinderen, MD (CHU Nantes, PI for site), Franck Durif, MD (CHU Clermont-Ferrand, PI for site), Richard Levy, MD (CHU Saint-Antoine, Paris, PI for site), Fernando Pico, MD (CH Versailles, PI for site), Olivier Rascol, MD (CHU Toulouse, PI for site); Co-investigators: Cecilia Bonnet, MD (Pitié-Salpêtrière Hospital, Paris, site investigator), Christine Brefel-Courbon, MD (CHU Toulouse, site investigator), Florence Cormier-Dequaire, MD (Pitié-Salpêtrière Hospital, Paris, site investigator), Andreas Hartmann, MD (Pitié-Salpêtrière Hospital, Paris, site investigator), Stephan Klebe, MD (Pitié-Salpêtrière Hospital, Paris, site investigator), Julia Kraemmer, MD (Pitié-Salpêtrière Hospital, site investigator), Lucette Lacomblez (Pitié-Salpêtrière Hospital, Paris, site investigator), Graziella Mangone, MD (Pitié-Salpêtrière Hospital, Paris, site investigator), Ana-Raquel Marques, MD (CHU Clermont Ferrand, site investigator), Valérie Mesnage, MD (CHU Saint Antoine, Paris, site investigator), Julia Muellner (Pitié-Salpêtrière Hospital, Paris, site investigator), Fabienne Ory-Magne, MD (CHU Toulouse, site investigator), Hana You (Pitié-Salpêtrière Hospital, Paris, site investigator); Neuropsychologists: Eve Benchetrit, MS (Pitié-Salpêtrière Hospital, Paris, neuropsychologist), Julie Socha, MS (Pitié-Salpêtrière Hospital, Paris, neuropsychologist), Fanny Pineau, MS (Pitié-Salpêtrière Hospital, Paris,

neuropsychologist); Sponsor activities and clinical research assistants: Alain Mallet, PhD (Pitié-Salpêtrière Hospital, Paris, sponsor representative), Coralie Villeret (Hôpital Saint Louis, Paris, Project manager), Merry Mazmanian (Pitié-Salpêtrière Hospital, Paris, project manager), Hakima Manseur (Pitié-Salpêtrière Hospital, Paris, clinical research assistant), Mostafa Hajji (Pitié-Salpêtrière Hospital, Paris, data manager), Benjamin Le Toullec, MS (Pitié-Salpêtrière Hospital, Paris, clinical research assistant).

AMP PD acknowledgements

Data used in the preparation of this article were obtained from the Accelerating Medicine Partnership® (AMP®) Parkinson's Disease (AMP PD) Knowledge Platform. For up-to-date information on the study, visit https://www.amp-pd.org.

The AMP® PD program is a public-private partnership managed by the Foundation for the National Institutes of Health and funded by the National Institute of Neurological Disorders and Stroke (NINDS) in partnership with the Aligning Science Across Parkinson's (ASAP) initiative; Celgene Corporation, a subsidiary of Bristol-Myers Squibb Company; GlaxoSmithKline plc (GSK); The Michael J. Fox Foundation for Parkinson's Research ; Pfizer Inc.; Sanofi US Services Inc.; and Verily Life Sciences.

ACCELERATING MEDICINES PARTNERSHIP and AMP are registered service marks of the U.S. Department of Health and Human Services.

AMP PD Cohort Acknowledgements

Clinical data and biosamples used in preparation of this article were obtained from the (i) Michael J. Fox Foundation for Parkinson's Research (MJFF) and National Institutes of Neurological Disorders and Stroke (NINDS) BioFIND study, (ii) Harvard Biomarkers Study (HBS), (iii) National Institute on Aging (NIA) International Lewy Body Dementia Genetics Consortium Genome Sequencing in Lewy Body Dementia Case-control Cohort (LBD), (iv) MJFF LRRK2 Cohort Consortium (LCC), (v) NINDS Parkinson's Disease Biomarkers Program (PDBP), (vi) MJFF Parkinson's Progression Markers Initiative (PPMI), and (vii) NINDS Study of Isradipine as a Disease-modifying Agent in Subjects With Early Parkinson Disease, Phase 3 (STEADY-PD3) and (viii) the NINDS Study of Urate Elevation in Parkinson's Disease, Phase 3 (SURE-PD3).

BioFIND is sponsored by The Michael J. Fox Foundation for Parkinson's Research (MJFF) with support from the National Institute for Neurological Disorders and Stroke (NINDS). The BioFIND Investigators have not participated in reviewing the data analysis or content of the manuscript. For up-to-date information on the study, visit michaeljfox.org/news/biofind.

Genome sequence data for the Lewy body dementia case-control cohort were generated at the Intramural Research Program of the U.S. National Institutes of Health. The study

was supported in part by the National Institute on Aging (program #: 1ZIAAG000935) and the National Institute of Neurological Disorders and Stroke (program #: 1ZIANS003154).

The Harvard Biomarker Study (HBS) is a collaboration of HBS investigators [full list of HBS investigators found at https://www.bwhparkinsoncenter.org/biobank/ and funded through philanthropy and NIH and Non-NIH funding sources. The HBS Investigators have not participated in reviewing the data analysis or content of the manuscript.

Data used in preparation of this article were obtained from The Michael J. Fox Foundation sponsored LRRK2 Cohort Consortium (LCC). The LCC Investigators have not participated in reviewing the data analysis or content of the manuscript. For up-to-date information on the study, visit https://www.michaeljfox.org/biospecimens).

PPMI is sponsored by The Michael J. Fox Foundation for Parkinson's Research and supported by a consortium of scientific partners: [list the full names of all of the PPMI funding partners found at https://www.ppmi-info.org/about-ppmi/who-we-are/study-sponsors]. The PPMI investigators have not participated in reviewing the data analysis or content of the manuscript. For up-to-date information on the study, visit www.ppmi-info.org.

The Parkinson's Disease Biomarker Program (PDBP) consortium is supported by the National Institute of Neurological Disorders and Stroke (NINDS) at the National Institutes of Health. A full list of PDBP investigators can be found at https://pdbp.ninds.nih.gov/policy. The PDBP investigators have not participated in reviewing the data analysis or content of the manuscript.

The Study of Isradipine as a Disease-modifying Agent in Subjects With Early Parkinson Disease, Phase 3 (STEADY-PD3) is funded by the National Institute of Neurological Disorders and Stroke (NINDS) at the National Institutes of Health with support from The Michael J. Fox Foundation and the Parkinson Study Group. For additional study information, visit https://clinicaltrials.gov/ct2/show/study/NCT02168842. The STEADY-PD3 investigators have not participated in reviewing the data analysis or content of the manuscript.

The Study of Urate Elevation in Parkinson's Disease, Phase 3 (SURE-PD3) is funded by the National Institute of Neurological Disorders and Stroke (NINDS) at the National Institutes of Health with support from The Michael J. Fox Foundation and the Parkinson Study Group. For additional study information, visit https://clinicaltrials.gov/ct2/show/NCT02642393. The SURE-PD3 investigators have not participated in reviewing the data analysis or content of the manuscript.

SUPPLEMENTARY REFERENCES

1. Nalls, M. A. *et al.* Identification of novel risk loci, causal insights, and heritable risk for Parkinson's disease: a meta-analysis of genome-wide association studies. *Lancet Neurol.* **18**, 1091–1102 (2019).
2. Iwaki, H. *et al.* Genomewide association study of Parkinson's disease clinical biomarkers in 12 longitudinal patients' cohorts. *Mov. Disord.* 1–12 (2019) doi:10.1002/mds.27845.
3. Mitchell, R. E. *et al.* Strategies to investigate and mitigate collider bias in genetic and Mendelian randomisation studies of disease progression. *PLoS Genet.* **19**, 1–24 (2023).
4. Mahmoud, O., Dudbridge, F., Davey Smith, G., Munafo, M. & Tilling, K. A robust method for collider bias correction in conditional genome-wide association studies. *Nat. Commun.* **13**, 1–13 (2022).
5. Van Der Harst, P. & Verweij, N. Identification of 64 novel genetic loci provides an expanded view on the genetic architecture of coronary artery disease. *Circ. Res.* **122**, 433–443 (2018).
6. Matsunaga, H. *et al.* Transethnic Meta-Analysis of Genome-Wide Association Studies Identifies Three New Loci and Characterizes Population-Specific Differences for Coronary Artery Disease. *Circ. Genomic Precis. Med.* 128–138 (2020) doi:10.1161/CIRCGEN.119.002670.
7. Koyama, S. *et al.* Population-specific and trans-ancestry genome-wide analyses identify distinct and shared genetic risk loci for coronary artery disease. *Nat. Genet.* **52**, 1169–1177 (2020).
8. Shadrina, A. S. *et al.* Prioritization of causal genes for coronary artery disease based on cumulative evidence from experimental and in silico studies. *Sci. Rep.* **10**, 1–15 (2020).
9. Timmers, P. R. H. J., Wilson, J. F., Joshi, P. K. & Deelen, J. Multivariate genomic scan implicates novel loci and haem metabolism in human ageing. *Nat. Commun.* **11**, 1–10 (2020).
10. Ben-Shlomo, Y. & Marmot, M. G. Survival and cause of death in a cohort of patients with parkinsonism: Possible clues to aetiology? *J. Neurol. Neurosurg. Psychiatry* **58**, 293–299 (1995).
11. Cao, P., Maximov, A. & Südhof, T. C. Activity-dependent IGF-1 exocytosis is controlled by the Ca 2+-sensor synaptotagmin-10. *Cell* **145**, 300–311 (2011).
12. Castilla-Cortázar, I., Aguirre, G. A., Femat-Roldán, G., Martín-Estal, I. & Espinosa, L. Is insulin-like growth factor-1 involved in Parkinson's disease development? *J. Transl. Med.* **18**, 1–17 (2020).
13. Pristerà, A. *et al.* Dopamine neuron-derived IGF-1 controls dopamine neuron firing, skill learning, and exploration. *Proc. Natl. Acad. Sci. U. S. A.* **116**, 3817–3826 (2019).
14. Lomelino, C. L., Andring, J. T., McKenna, R. & Kilberg, M. S. Asparagine synthetase: Function, structure, and role in disease. *J. Biol. Chem.* **292**, 19952–19958 (2017).
15. Granzotto, A., Canzoniero, L. M. T. & Sensi, S. L. A Neurotoxic Ménage-à-trois: Glutamate, Calcium, and Zinc in the Excitotoxic Cascade. *Front. Mol. Neurosci.* **13**,

1–13 (2020).
16. Mehta, A., Prabhakar, M., Kumar, P., Deshmukh, R. & Sharma, P. L. Excitotoxicity: Bridge to various triggers in neurodegenerative disorders. *Eur. J. Pharmacol.* **698**, 6–18 (2013).
17. Zhang, Q. *et al.* L-Asparaginase Exerts Neuroprotective Effects in an SH-SY5Y-A53T Model of Parkinson's Disease by Regulating Glutamine Metabolism. *Front. Mol. Neurosci.* **13**, 1–15 (2020).
18. Erro, R., Mencacci, N. E. & Bhatia, K. P. The Emerging Role of Phosphodiesterases in Movement Disorders. *Mov. Disord.* **36**, 2225–2243 (2021).
19. Tozzi, A. *et al.* Mechanisms underlying altered striatal synaptic plasticity in old A53T-α synuclein overexpressing mice. *Neurobiol. Aging* **33**, 1792–1799 (2012).
20. Giorgi, M. *et al.* Lowered cAMP and cGMP signalling in the brain during levodopa-induced dyskinesias in hemiparkinsonian rats: New aspects in the pathogenetic mechanisms. *Eur. J. Neurosci.* **28**, 941–950 (2008).
21. Kelly, M. P. Cyclic nucleotide signaling changes associated with normal aging and age-related diseases of the brain. *Cell. Signal.* **42**, 281–291 (2018).
22. Young, M. J., Hsu, K. C., Lin, T. E., Chang, W. C. & Hung, J. J. The role of ubiquitin-specific peptidases in cancer progression. *J. Biomed. Sci.* **26**, 1–14 (2019).
23. Yao, Y. *et al.* USP53 plays an antitumor role in hepatocellular carcinoma through deubiquitination of cytochrome c. *Oncogenesis* **11**, 1–12 (2022).
24. Cheng, W. *et al.* USP53 activated by H3K27 acetylation regulates cell viability, apoptosis and metabolism in esophageal carcinoma via the AMPK signaling pathway. *Carcinogenesis* **43**, 349–359 (2022).
25. Xu, D., Farmer, A., Collett, G., Grishin, N. V. & Chook, Y. M. Sequence and structural analyses of nuclear export signals in the NESdb database. *Mol. Biol. Cell* **23**, 3677–3693 (2012).
26. Azmi, A. S., Uddin, M. H. & Mohammad, R. M. The nuclear export protein XPO1 — from biology to targeted therapy. *Nat. Rev. Clin. Oncol.* **18**, 152–169 (2021).
27. Liu, S. *et al.* The XPO1 Inhibitor KPT-8602 Ameliorates Parkinson's Disease by Inhibiting the NF-κB/NLRP3 Pathway. *Front. Pharmacol.* **13**, 1–14 (2022).
28. Lui, T. T. H. *et al.* The Ubiquitin-Specific Protease USP34 Regulates Axin Stability and Wnt/β-Catenin Signaling. *Mol. Cell. Biol.* **31**, 2053–2065 (2011).
29. Pascual, V. *et al.* Different gene expression signatures in children and adults with celiac disease. *PLoS One* **11**, 1–10 (2016).
30. Gabrielsen, I. S. M. *et al.* Genetic risk variants for autoimmune diseases that influence gene expression in thymus. *Hum. Mol. Genet.* **25**, 3117–3124 (2016).
